# *bHLH142* regulates various metabolic pathway-related genes to affect pollen development and anther dehiscence in rice

**DOI:** 10.1038/srep43397

**Published:** 2017-03-06

**Authors:** Rajeev Ranjan, Reema Khurana, Naveen Malik, Saurabh Badoni, Swarup K. Parida, Sanjay Kapoor, Akhilesh K. Tyagi

**Affiliations:** 1 National Institute of Plant Genome Research (NIPGR), Aruna Asaf Ali Marg, New Delhi 110067, India; 2 Department of Plant Molecular Biology, University of Delhi South Campus, Benito Juarez Marg, New Delhi 110021, India

## Abstract

Apposite development of anther and its dehiscence are important for the reproductive success of the flowering plants. Recently, *bHLH142*, a bHLH transcription factor encoding gene of rice has been found to show anther-specific expression and mutant analyses suggest its functions in regulating tapetum differentiation and degeneration during anther development. However, our study on protein level expression and gain-of-function phenotype revealed novel aspects of its regulation and function during anther development. Temporally dissimilar pattern of *bHLH142* transcript and polypeptide accumulation suggested regulation of its expression beyond transcriptional level. Overexpression of *bHLH142* in transgenic rice resulted in indehiscent anthers and aborted pollen grains. Defects in septum and stomium rupture caused anther indehiscence while pollen abortion phenotype attributed to abnormal degeneration of the tapetum. Furthermore, RNA-Seq-based transcriptome analysis of tetrad and mature pollen stage anthers of wild type and *bHLH142*^*OE*^plants suggested that it might regulate carbohydrate and lipid metabolism, cell wall modification, reactive oxygen species (ROS) homeostasis and cell death-related genes during rice anther development. Thus, *bHLH142* is an anther-specific gene whose expression is regulated at transcriptional and post-transcriptional/translational levels. It plays a role in pollen maturation and anther dehiscence by regulating expression of various metabolic pathways-related genes.

Major events in the anther development are differentiation of stamen primordia, development of microspore and then dehiscence of the anther. Typical anther has four locules and each locule consists of four-layered anther wall in which the developing microspore resides^1^,^2^. Each layer of the anther wall performs specialised function during the process of anther development^3^. Epidermis is the outer cover that protects anther from various environmental stresses and also forms specialised structures named as stomium and septum, which are involved in anther dehiscence process^4^,^5^. Endothecium is the second layer that develops secondary thickening in the form of lignin deposition, which assists the process of anther dehiscence. Middle layer is present between endothecium and tapetum, which undergoes degeneration during pollen maturation along with the tapetum. Tapetum is the innermost layer, which undergoes programmed cell death (PCD)-mediated degeneration to release nutritive components for developing pollen and sporopollenin as well as other pollen wall precursors^6^. Proper development and timely degeneration of specific cell types in the anther wall layer is essential for development and dispersal of pollen grains^7^,^8^,^9^. Various cellular and metabolic changes occur during the formation and degeneration of each layer. In recent years, role of bHLH transcription factors in various aspects of anther development has been elucidated. *DYSFUNCTIONAL TAPETUM1 (DYT1)* from Arabidopsis and its rice homolog *UNDEVELOPED TAPETUM1 (UDT1)* reportedly regulate the tapetum development process. Mutant plants of both the genes displayed hypertrophic growth and abnormal vacuolation of tapetum^10^,^11^,^12^. Various lipid metabolism, cell-wall modification and secondary metabolism-related genes were downregulated in the *dyt1* mutant^12^. Furthermore, bHLH10, bHLH89 and bHLH91 have been shown to interact with DYT1 and work redundantly during anther development^13^. Similarly, mutation in the *ABORTED MICROSPORE1 (AMS1)* gene in Arabidopsis, and its rice ortholog, *TAPETUM DEGENERATION RETARDATION (TDR)* result in pollen abortion and a hypertrophic tapetum^14^,^15^. AMS interacts with bHLH89 and bHLH91 and acts as a master regulator of pollen wall development by directly regulating expression of genes related to various metabolic processes^16^,^17^. TDR affects the metabolism of fatty acids and other aliphatic compounds besides regulating tapetum degeneration by directly regulating the expression of a cysteine protease gene *CP1*^14^,^18^. Furthermore, another bHLH transcription factor, ETERNAL TAPETUM1 (EAT1) has been shown to interact with TDR and regulates tapetal PCD in rice^19^. Recently, mutant analyses of *bHLH142* from rice revealed its role in tapetum differentiation and degeneration during post-meiotic anther development^20^,^21^.

Modified epidermal tissues including septum and stomium along with endothecium are involved in the process of anther dehiscence^22^. A number of genes that are implicated in the process of anther dehiscence have been identified from forward and reverse genetics studies in Arabidopsis and rice^23^. *DEFECTIVE IN ANTHER DEHISCENCE1 (DAD1)* gene encodes phospholipase enzyme required for jasmonic acid biosynthesis and regulates anther dehiscence in Arabidopsis. Knock-down/Knock-out of *SIZ1*, a SUMO E3 ligase gene, resulted in sterile rice plant due to lack of anther dehiscence^24^. *Pollen Semi-Sterility1 (PSS1)* encodes a kinesin-like protein that regulates both pollen development and anther dehiscence process in rice^25^. A rice MYB transcription factor encoding gene *ANTHER INDEHISCENCE1 (AID1)* has been shown to be involved in anther dehiscence process by regulating septum and stomium degradation^26^. A mutation in another MYB gene *MALE STERILE35 (MYB26)* from Arabidopsis leads to male sterility because of anther indehiscence^27^,^28^. Furthermore, *MYB26* has been found to regulate secondary thickening of endothecium by affecting the expression of genes related to lignin deposition in secondary walls^27^. Thickening of the endothecium secondary wall in Arabidopsis anther is known to be regulated by NAC transcription factors NST1 and NST2^29^.

Previously, we had characterised the promoter of *bHLH142* through transgenic approaches and shown its capability to impart anther-specific expression to the reporter gene^30^. In this study, we show that although the *bHLH142* transcript accumulation peaks during early stages of anther development the resultant protein accumulates in a biphasic manner; once at the tetrad stage of the anther and then in mature anther, in spite of relatively low levels of its transcript being present in the mature anther. Phenotypic as well as transcriptome analysis of *bHLH142*^*OE*^ transgenic plants revealed that *bHLH142* regulates anther dehiscence and tapetum degeneration process by affecting cell wall degradation and ROS signalling-related genes and it controls pollen maturation by affecting carbohydrate and lipid metabolism-related genes.

## Results

### *bHLH142* shows biphasic expression pattern at protein level

**Figure 1:**
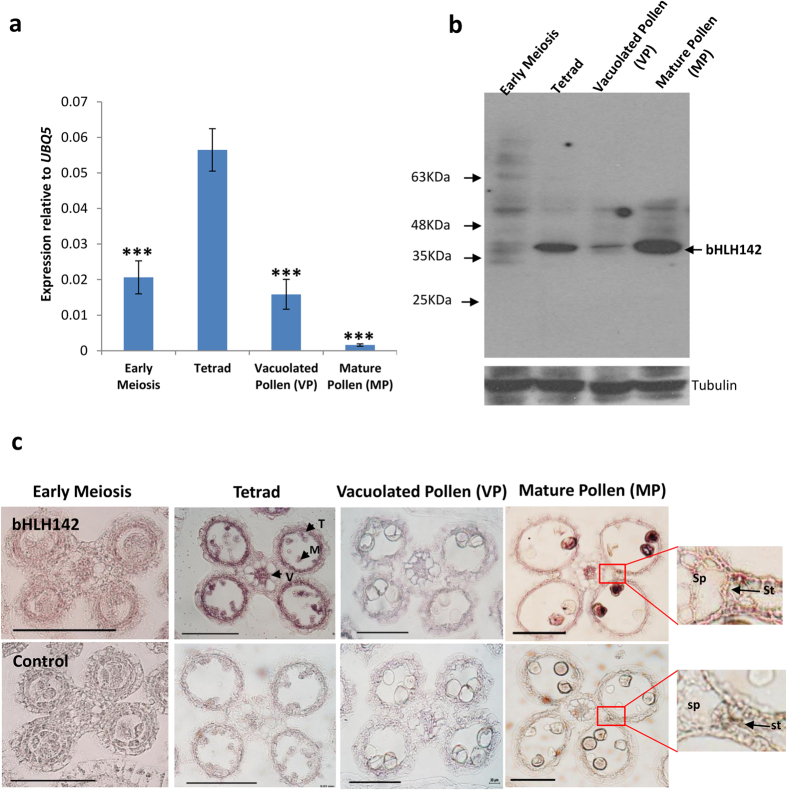
Temporal expression pattern of transcript and protein for *bHLH142* are different. **(a)** qRT-PCR expression analysis of *bHLH142* transcript in different stages of rice anthers. Error bars indicate standard deviation (SD). The data are presented as the mean ± SD (n = 3). Asterisks indicate significant difference with respect to Tetrad (‘***’ indicates *t*-test, p-value ≤ 0.001). **(b)** Immunoblot showing accumulation of bHLH142 polypeptide in different stages of rice spikelets. **(c)** *In situ* immunolocalization showing spatio-temporal presence of bHLH142 polypeptide during different stages of rice anthers. Right most panels show enlarged view of the selected region (shown as rectangle) of MP stage of the anther. Control panel shows samples without bHLH142 primary antibody treatment. M, Microspore; sp, Septum; st, Stomium; T, Tapetum; V, Vascular tissue. Scales: 100 μm.

In a previous report, we showed that *bHLH142* is an anther-specific gene in rice and its promoter displayed maximum activity in meiotic anther^30^. Later, RNA-*in situ* hybridisation studies by Fu *et al*.^21^ and Ko *et al*.^20^ also confirmed that *bHLH142* transcripts accumulate predominantly at the meiotic anther stage. The accumulation of *bHLH142* mRNA and its protein during different stages of anther development was analysed to gain more insight into its expression pattern by qRT-PCR (Quantitative Real Time-PCR) and immunoblotting techniques, respectively. Consistent with the previous reports, *bHLH142* mRNA was detected in early meiosis stage and showed maximum abundance in tetrad stage. After these stages, the transcript levels declined in the vacuolated pollen (VP) stage and were barely detectable in the mature pollen (MP) stage of anther development (Fig. 1a). Furthermore, bHLH142 protein sequence analysis showed two bipartite and three monopartite nuclear localization signals, which suggested its possible nuclear localization (Figure S1). To confirm this, subcellular localization study was performed and result showed that bHLH142-YFP fusion protein localised exclusively into the nucleus (Figure S1). Furthermore, accumulation of bHLH142 protein during different stages of rice anther development was examined through immunoblotting. A faint band of bHLH142 protein was detected at early meiosis stage and vacuolated pollen stage but a rather strong expression was observed at tetrad and MP stage (Fig. 1b). This suggests that bHLH142 protein accumulated in tetrad as well as in MP stage of the anther. Detection of bHLH142 protein in MP stage was surprising as neither a significant promoter activity nor the transcript was reported at this stage^20^,^21^,^30^. Consistent results from three independent experiments confirmed the presence of bHLH142 polypeptide in the MP stage of rice anther. To rule out that the hybridisation with other bHLH proteins could give false signals in mature anthers, the anti-bHLH142 antibody was tested for cross reactivity with two of its closest homologues, EAT1 and TDR. The absence of any cross-reactivity proved accumulation of bHLH142 in mature anthers (Figure S1). To investigate distribution of bHLH142 protein in different cell types of anther during different stages and to substantiate its detection at MP stage, *in situ* immunolocalization study was performed. Outcome supported the immunoblot data, as a faint signal was detected in anther cross-sections from early meiosis stage and vacuolated pollen stage compared to strong signal in tetrad and MP stage (Fig. 1c). Furthermore, bHLH142 polypeptide was detected in the tapetum and microspores during tetrad stage and in epidermis and pollen grains at MP stage. Enlarged view of the wall layers of MP anther showed presence of immune signal in the septum and stomium regions. In addition, expression signal was also detected in vascular tissue of tetrad and MP stage of the anther. Control samples showed only background signals (Fig. 1c). Therefore, cell type-specific distribution of *bHLH142* mRNA^20^,^21^ and the polypeptide appeared similar, as both were detected in tapetum, microspores and vascular tissues. However, the levels of the transcript and the polypeptide varied during different anther stages, as although the mRNA was present in negligible amounts at the MP stage, the protein accumulated in high amounts. This suggests that there is temporal difference in expression pattern of *bHLH142* transcript and polypeptide while spatial pattern is similar.

### Overexpression of *bHLH142* causes defects in degeneration of septum and stomium that leads to indehisced anther

**Figure 2:**
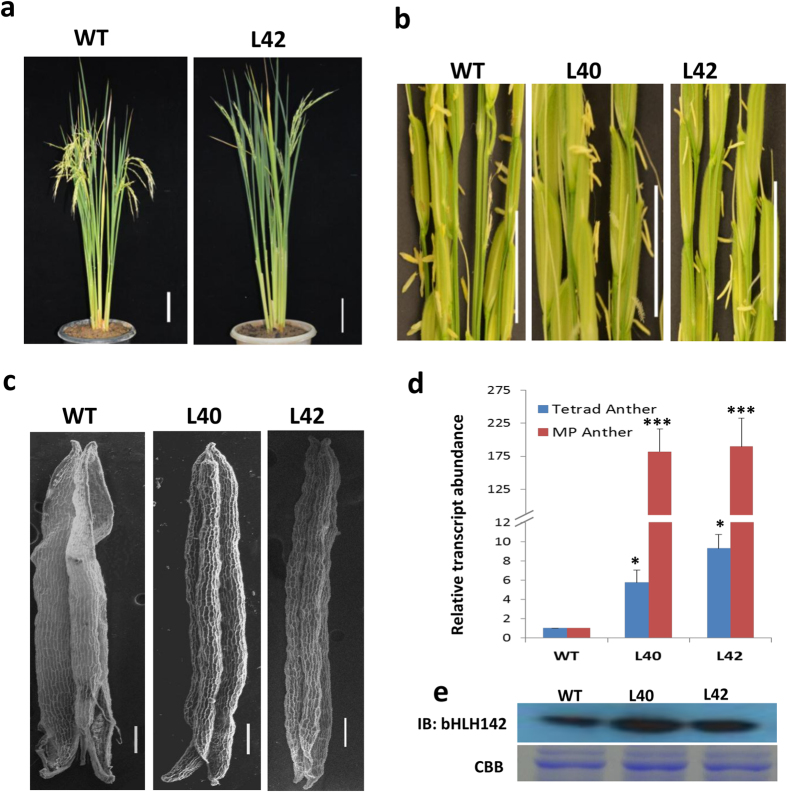
Overexpression of *bHLH142* causes anther indehiscence in rice. (**a)** *bHLH142*^*OE*^plant (L42) showing growth compared to WT. **(b)** Post-anthesis panicles of WT and *bHLH142*^*OE*^ transgenics (L40 and L42) showing dehisced anthers in WT and indehisced anther in transgenic plants. **(c)** SEM images of WT and transgenic anthers showing that apical and basal part of WT anther is ruptured while that of transgenics is intact. **(d)** qRT-PCR expression analysis showing high accumulation of *bHLH142* transcripts in transgenic anthers at tetrad and MP stages. Error bars indicate standard deviation (SD). The data are presented as the mean ± SD (n = 3). Asterisks indicate significant difference with respect to WT (‘*’ indicates *t*-test p-value ≤ 0.05 while ‘***’ indicates p-value ≤ 0.001). **(e)** Immunoblot showing higher accumulation of bHLH142 polypeptide in transgenic plants in MP anther. WT, Wild Type.

Detection of bHLH142 protein in biphasic manner at tetrad and MP stage anthers raised the possibility of its function during both the stages. However, recent studies of *bHLH142* mutants suggested its role only during tetrad stage in controlling tapetum development/degeneration^20^,^21^. Functional characterisation of a gene by gain-of-function approach helps in exploring its novel functions during plant growth and development^31^,^32^,^33^,^34^. Therefore, we used gain-of-function approach to get more insight into biological functions of *bHLH142* during rice anther development. Rice transgenics overexpressing *bHLH142* under the control of a maize ubiquitin promoter were raised (Figure S2 and Table S1). At least seven independent positive transgenic plants showed high level of *bHLH142* transcript accumulation (Figure S2). All of them showed normal vegetative growth, but none of them was able to set seeds. However, manual pollination of transgenic plants with wild type (WT) pollen resulted in seed formation (Figure S3). This suggested that defects in the male reproductive development affected the seed set. Seeds obtained from cross pollination were grown and resultant plants showed segregation of hygromycin-resistant marker gene into 1:1 ratio (Figure S3). Positive T_1_ plants were again sterile and not able to form seeds despite having normal vegetative growth (Fig. 2a). Observation of the male reproductive organs in WT and transgenic plants revealed that anthers of the transgenic plants did not dehisce even after the complete maturation (Fig. 2b). All analyses were performed in these T1 transgenic plants. However, to look at the stability of phenotype, we observed one more generation and each time similar results were obtained, suggesting that the phenotype was stably inherited in transgenic plants (Figure S3).

**Figure 3:**
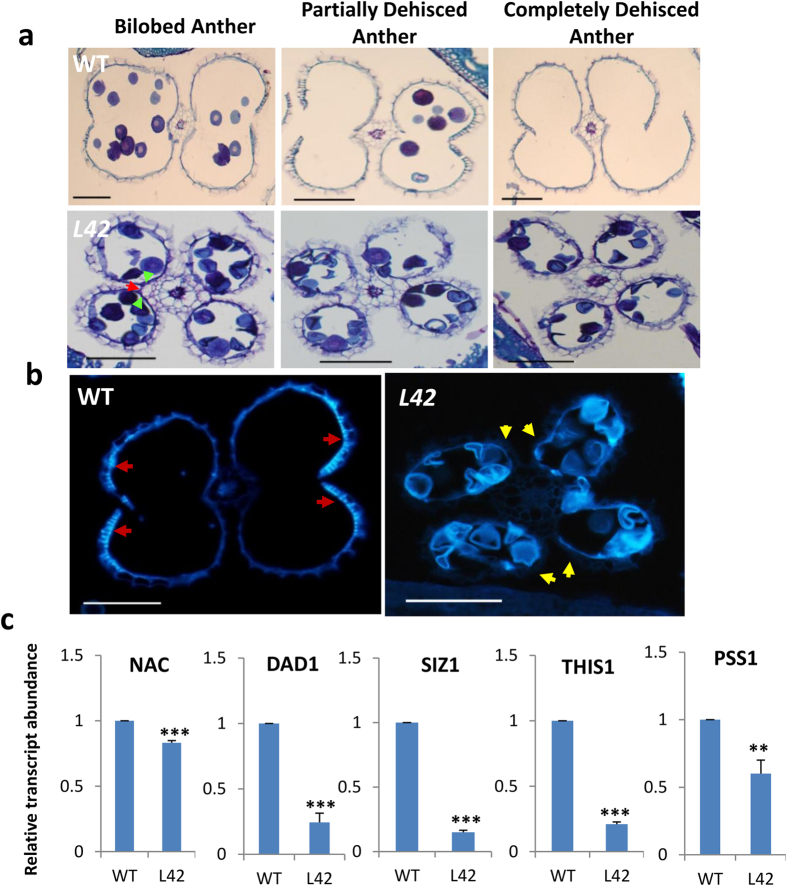
*bHLH142* overexpressing anthers are defective in septum and stomium degeneration. **(a)** Transverse sections of pre-anthesis staged WT and transgenic anthers (L42) undergoing dehiscence. Green and red arrows indicate intact septum and stomium in transgenic anthers, respectively. **(b)** WT and transgenic anthers analyzed under UV light to visualize lignin autofluorescence of endothecium regions. Red and yellow arrows indicate presence and absence secondary thickening of endothecium, respectively. **(c)** qRT-PCR expression data of anther dehiscence-related protein genes. Error bars indicate standard deviation (SD). The data are presented as the mean ± SD (n = 3). Asterisks indicate significant difference with respect to WT (‘***’ indicates *t*-test p-value ≤ 0.001, while ‘**’ indicates p-value ≤ 0.01). WT, Wild Type. Scales: A, 50 μm; B, 100 μm.

We compared the anther dehiscence phenomenon in the transgenics with that in the WT. Scanning electron microscopic observations of the post-anthesis-staged anthers revealed that the apical and basal parts of the transgenic anthers remained intact while they were found to be ruptured in WT (Fig. 2c). *bHLH142* transcripts as well as polypeptide were highly overexpressed in *bHLH142*^*OE*^transgenic plants compared to WT as analysed by qRT-PCR and immunoblotting (Fig. 2d,e). Furthermore, observation of transverse sections of WT and transgenic spikelets at mature stage showed that anther dehiscence in WT was initiated by degradation of septum tissue, which causes two anther locules to fuse together. Then another set of specialised cells forming stomium at the junction of two locules ruptured to complete the process of dehiscence and allow the release of pollen grains (Fig. 3a). However, *bHLH142*^*OE*^ anther locules did not fuse together owing to intact septum and also stomium did not rupture, preventing the release of pollen grains from the anther (Fig. 3a). Moreover, in WT plants, thickening of endothecium as band-like structure near the junction of the two locules was observed in mature anther undergoing dehiscence but such structure was absent in transgenic anthers (Fig. 3a). To confirm this, mature anther sections were analysed under UV light in fluorescence microscope and endothecium thickenings due to lignin deposition were found to be absent in transgenic anthers (Fig. 3b). Furthermore, we checked the expression of genes associated with anther dehiscence in rice and found that *DAD1, SIZ1* and *THIS1* were downregulated in the *bHLH142*^*OE*^ anthers (Fig. 3c).

### *bHLH142* overexpressing anther produces defective and aborted pollen grains

**Figure 4:**
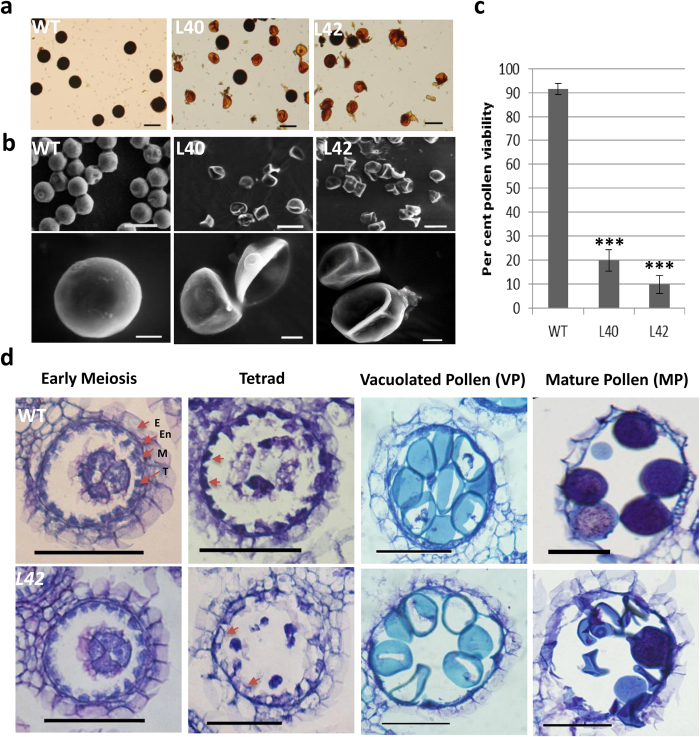
*bHLH142* overexpressing anthers produce defective pollen grains. **(a)** I_2_-KI staining of pollen grains from WT and transgenic plants (L40 and L42). **(b)** SEM images of WT and transgenic pollen grains (L40 and L42). **(c)** Quantitative estimation of pollen viability through I_2_-KI staining. Error bars indicate standard deviation (SD). The data are presented as the mean ± SD (n = 3). Asterisks indicate significant difference with respect to WT (‘***’ indicates *t*-test, p-value ≤ 0.001). **(d)** Transverse sections of WT and transgenic anthers (L42) during various stages of development. Arrows in the tetrad panel indicate well developed and poorly developed tapetum in WT and transgenic anthers, respectively. WT, Wild Type; E, Epidermis; En, Endothecium; M, Middle layer; T, Tapetum. Scales: (**a**), 50 μm; (**b**), upper panel 50 μm, lower panel 10 μm; (**d**), 50 μm.

**Figure 5:**
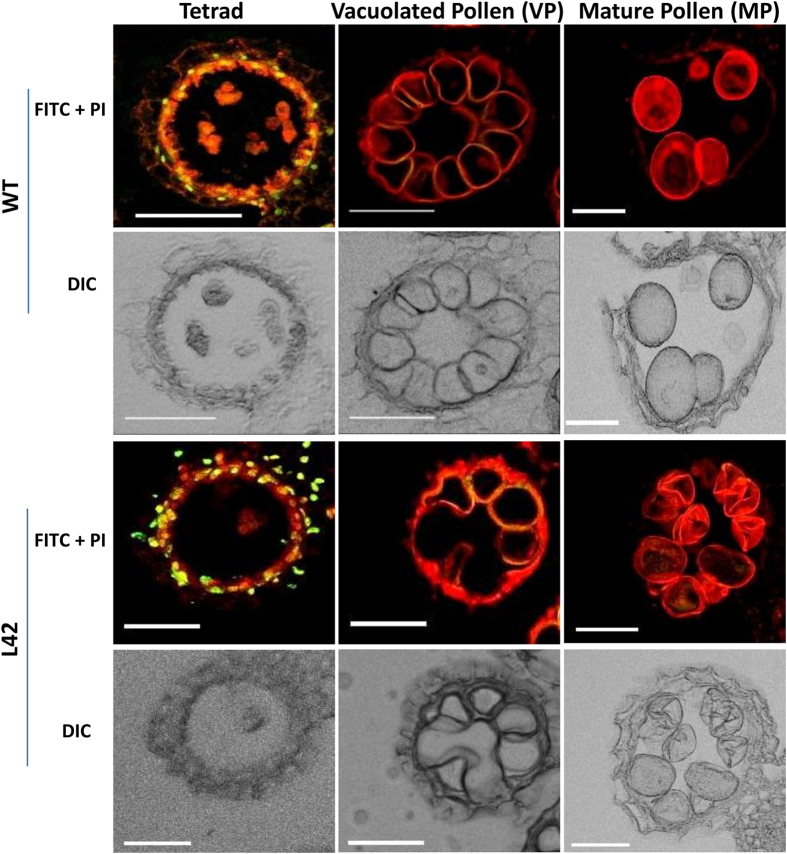
*bHLH142* overexpressing anther shows abnormal programmed cell death in tapetum. TUNEL assay showing PCD undergoing cells as greenish-yellow signal due to merging of FITC signals and propidium iodide (PI) stain and normal cells appear red due to propidium iodide stain only. Images were taken from confocal microscope in FITC (Fluorescein isothiocyanate), PI and DIC (Differential interference contrast) channels. Merge image of FITC and PI are shown in fluorescent panel while DIC images are shown separately. Scales: 50 μm.

To explore the male reproductive development in *bHLH142*^*OE*^ plant we analysed its pollen development process. Most of the pollen grains of transgenic plants were found to be non-viable as they did not take up I_2_-KI stain (Fig. 4a). Electron microscopic analysis showed that transgenic pollen grains were shrunken and abnormal in shape when compared to WT (Fig. 4b). We tested pollen viability in several transgenic lines in T_0_, T_1_ and T_2_ generations and all showed high reduction in the viability (Figs 4c and S3). However, viable pollen grains showed normal pollen germination similar to WT (Figure S3) and manually dehisced anthers were able to cause set seed. This suggests that overexpression of *bHLH142* affects pollen development but surviving pollen grains have ability to fertilize the female gametes. To explore the pollen development process in transgenics, paraffin sections of the WT and *bHLH142*^*OE*^ anthers at different stages of development were analysed. No developmental differences were observed during early meiosis stage. All the four anther layers namely; epidermis, endothecium, middle layer and tapetum appeared to be differentiated and enlarged, nucleated microspore mother cells were also seen. At tetrad stage, WT anther had well organised deeply stained tapetal cell layer while transgenic anther tapetal cells appeared distorted and poorly stained (Fig. 4d). It also appeared that tapetum had loosely filled cytoplasm compared to that in the WT. However, no difference in microspore development was observed till vacuolated pollen stage (Fig. 4d). However, at MP stage most of the transgenic microspores failed to mature and eventually aborted, while WT pollen were filled with starch and attained maturity. These observations suggested some alteration in tapetum development in transgenic plants that probably restricted the pollen maturation process. It is known that tapetum undergoes PCD-mediated degeneration after the meiosis stage to nourish the developing microspore/pollen grain^6^,^35^. To identify the reason behind appearance of the abnormal tapetal cells in transgenic anther, tapetum degeneration process in WT and transgenic anthers was studied through TUNEL (Terminal deoxynucleotidyl transferase dUTP Nick End Labeling) assay. In the WT anthers, positive TUNEL signal was observed only in tapetal cells of tetrad stage anther and not from vacuolated and mature pollen anther (Fig. 5). In *bHLH142*^*OE*^ anther also, TUNEL signal was detected only at tetrad stage but, intensity of the signal as well as the number of tapetal cells showing the signal was much more as compared to the WT. This suggested that *bHLH142* overexpressing transgenic anthers probably undergo faster tapetal degeneration compared to wild type anthers (Fig. 5). Hence, abnormal tapetum in transgenic anther may have appeared because of aberrant degeneration, which affected maturation of vacuolated microspores into mature pollen grains due to the lack of proper nutrition.

### *bHLH142* regulates cell wall degradation, carbohydrate and lipid metabolism and ROS homeostasis-related genes during rice anther development

**Figure 6:**
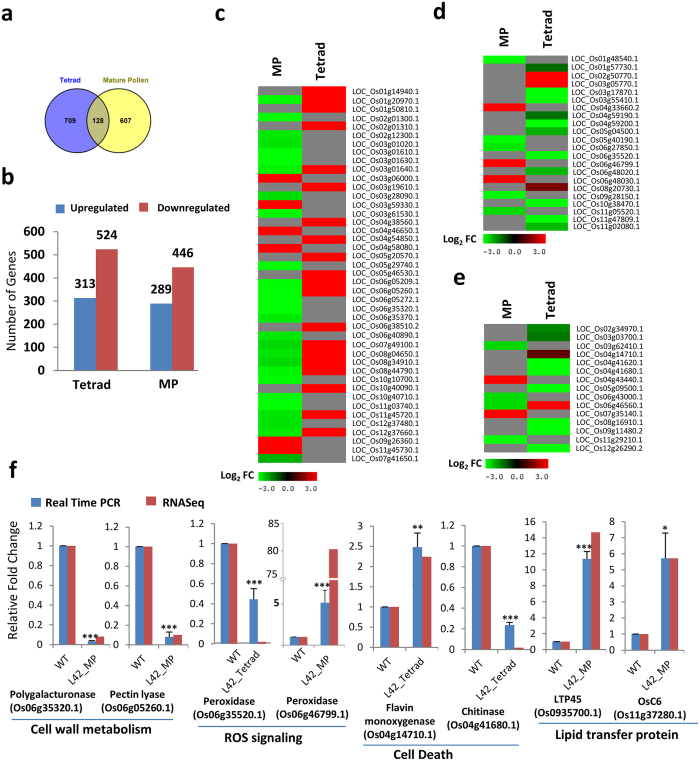
*bHLH142*^*OE*^ anthers show changed in expression of various metabolic pathway-related genes. **(a)** Venn diagram showing common and unique genes differentially regulated in tetrad and MP anther. **(b)** Histogram showing number of genes upregulated and downregulated in tetrad and MP stage of transgenic anthers compared to WT. Heat map showing log_2_ fold changed in expression of cell wall modification **(c),** ROS signaling **(d)** and cell death **(e)**-related genes. **(f)** Validation of changed in expression of different metabolism-related genes through qRT-PCR. The data are presented as the mean ± SD (n = 3). Asterisks indicate significant difference with respect to WT (‘***’ indicates *t*-test p-value ≤ 0.001, ‘**’ p-value ≤ 0.01 and ‘*’ p-value ≤ 0.05).

Transcription factors perform their biological functions through regulating expression of various signalling and metabolic pathways-related genes. Maximum expression of bHLH142 protein was detected at tetrad and MP stage of the rice anthers. Therefore, to identify the downstream gene regulatory network of *bHLH142,* RNA-Seq-based transcriptome analysis of tetrad and MP stage of WT and *bHLH142*^*OE*^ anthers was performed. Differentially expressed genes in both the stages of transgenic and WT anthers were identified. Total 837 genes in tetrad and 735 in MP anther were found to be differentially expressed in *bHLH142*^*OE*^ compared to WT. However, only 128 genes were found to be common in both the stages (Fig. 6a). In tetrad anther, 313 genes were upregulated and 524 were downregulated. In MP anther, 289 upregulated and 446 downregulated genes were identified (Fig. 6b). In order to classify these genes into functional categories, Gene Ontology (GO) terms were identified and their enrichment was performed. Furthermore, putative functions of each gene were retrieved from RGAP (Rice Genome Annotation Project). Regulation of anatomical structure, cell growth, cell size and cellular component size-related GO terms were enriched in differentially expressed genes at tetrad stage. Carbohydrate metabolic process, reproduction, enzyme and hydrolytic activity terms were enriched in MP stage (Figure S4).

Furthermore, few of the key genes already known for anther development were also found to be differentially expressed in *bHLH142*^*OE*^ plants (Table 1). Among these, *OsC4, OsC6, CYP704B2* and *CYP703A3* are known to be involved in pollen wall formation^36^,^37^,^38^,^39^ and *MST8, INV4, SUT3, UGP2* and *AGPL1* are known for sugar partitioning in reproductive tissues^13^,^40^. Moreover, genes belonging to different molecular and biological categories were found to be differentially expressed in *bHLH142*^*OE*^ plants in tetrad and MP anthers (Tables S2 and S3). By considering GO and putative function obtained through RGAP, it was observed that genes related to carbohydrate metabolism, lipid metabolism, cell wall modification, ROS homeostasis and cell death represent major categories among differentially expressed genes in *bHLH142* overexpressing anthers. Thirty-two genes in tetrad and thirty-eight genes in MP stage were found to be related with carbohydrate metabolism. Glycerophosphoryl diester phosphodiesterase, glycosyl transferase, polygalacturonase, glucan endo-1,3-beta-glucosidase and glycosyl hydrolase encoding genes were found to represent major carbohydrate metabolism-related differentially expressed genes (Table S4). Similarly, 23 genes in tetrad and 27 in MP were related to lipid metabolism. Among the lipid metabolism-related genes glycerophosphoryl diester phosphodiesterase, GDSL-like lipase/acylhydrolase genes were overrepresented (Table S5). Heat map showing expression of carbohydrate and lipid metabolism suggests that most of the genes are downregulated in both stages of anther (Figure S5). Furthermore, we obtained eight downregulated and two upregulated lipid transfer protein (LTP) encoding genes in tetrad stage and ten upregulated LTP genes in MP anther (Table S5). *OsC4, OsC6, LTPL45* and *CYP703A3* are notable LTP and lipid metabolism-related genes upregulated in MP stage, whose function in pollen development are already reported^36^,^37^,^38^. Changed expression of carbohydrate and lipid metabolism-related genes suggests that deposition of carbohydrate and lipid during pollen development is affected, which leads to formation of shrunken pollen in *bHLH142*^*OE*^ plants. Besides carbohydrate and lipid metabolism, genes involved in cell wall modification, ROS signalling and cell deaths were found to be other overrepresented classes in transcriptome data. Thirty genes in MP stage were found to be related to cell wall modification and majority of them were downregulated. Also, 23 genes from this category were found in tetrad stage and most of them were upregulated (Fig. 6c). Genes encoding for enzymes related to pectin degradation and modification like, pectate lyase, pectinesterase, pectin methylesterase, polygalacturonase and expansin were found to be overrepresented among cell wall modification-related genes. (Table S6). Defect in stomium and septum lysis and formation of aborted pollen in *bHLH142*^*OE*^ anther may be associated with down regulation of cell wall-related genes. Reactive oxygen species (ROS) signalling has been reported to play an important role during various growth and development processes. In the *bHLH142*^*OE*^ anthers, fourteen ROS homeostasis-related genes in tetrad and eight in MP anther were found to be differentially expressed (Fig. 6d). ROS signalling-related genes showing differential expression mainly include peroxidases. However, metallothionin, thioredoxin monodehyrdroscorbate and gultathione-s-transferase were also found to be differentially expressed (Table S7). Furthermore, we obtained ten genes in tetrad and six genes in MP stage that were assigned GO-term ‘cell death’ (Fig. 6e and Table S7). Changed expression of cell death-related genes in tetrad stage may be associated with the defective tapetum degeneration, while in MP, it may have altered septum and stomuim degenerations. Changes in expression of several genes from different categories were validated through qRT-PCR (Fig. 6f). Histological studies of *bHLH142*^*OE*^ plants showed that deposition of lignin was affected in transgenic anthers. We observed downregulation of some lignin biosynthesis-related genes in tetrad anther, which includes cinnamoyl CoA reductase, laccase and phenylalanine ammonia lyase (PAL) encoding genes. Furthermore, few water transporter protein aquaporin encoding genes were also downregulated in MP anthers (Table S8). Therefore, transcriptome analysis of *bHLH142*^*OE*^ tetrad and MP anthers revealed that overexpression of bHLH142 transcription factor may lead to disturbance of metabolic processes like lipid and carbohydrate metabolism, cell wall modification, ROS homeostasis and cell death-related processes.

## Discussion

Spatio-temporal regulation of gene expression determines many crucial developmental changes in plants. Gene regulation may occur at transcriptional, post-transcriptional, translational and post-translational levels. In the previous report, we have shown transcriptional level regulation of *bHLH142* expression. Expression of *GUS* reporter gene driven by *bHLH142* promoter was restricted to anther tissue in transgenic rice. However, in the Arabidopsis transgenic plants, *bHLH142* promoter: *GUS* construct showed ubiquitous expression pattern^30^. This suggests that *bHLH142* expression is transcriptionally regulated through its promoter and anther-specific activity is restricted only to rice or monocots. In this study, we have identified other aspects related to regulation of bHLH142 expression through protein level expression. We showed that there is a temporal difference between transcript and polypeptide accumulation of bHLH142, as its polypeptide was detected at high level in anthers at tetrad and MP stage, while negligible amount of the transcript was detected in anthers at MP stage. Previously, enhanced protein accumulation in germinating pollen compared to mature pollen was observed in case of *LAT52* gene in tobacco and tomato^41^. Delayed detection of bHLH142 polypeptide in comparison to its transcripts suggested regulation of its expression beyond transcriptional level. We hypothesise that stage-specific translation enhancement might be the reason for higher accumulation of bHLH142 protein in MP anther. Such kind of translation enhancement has previously been observed during tobacco pollen development and germination^41^,^42^. Transient as well as stable expression studies of sequence present within 5′ UTR region of *LAT52* gene caused increase in translational yield in developmentally regulated manner during pollen maturation^41^. Similarly, 5′ UTR region of *NTP303* gene also enhanced translation in pollen tube but not in mature pollen^42^. We found sequence similarity between 5′ UTR of *bHLH142, ntp303* and *LAT52* genes suggesting similar type of translation enhancement in case of bHLH142 in MP anther (Figure S6). Apart from this, the other possible reason behind biphasic protein accumulation of bHLH142 may lie in the enhancement of its protein stability. In the recent report, proteomic and phospho-proteomic analysis of rice meiotic anthers showed presence of bHLH142 protein in phospho-proteome^43^. Detection of bHLH142 protein in anther phospho-proteome suggested its post-translational level regulation that may change its stability. Although these hypotheses need more experimental validations to reach any specific conclusion, above-mentioned findings suggest that expression of *bHLH142* is regulated at transcriptional, post-transcriptional/translational and/or post-translational levels during rice anther development.

Previous reports showed that transgenic knock-down/mutant knock-out of *bHLH142* resulted in male sterile plants because of no pollen formation^20^,^21^. Mutants were defective in tapetum development and degeneration process that led to the pollen abortion. Our experiments have shown that overexpression of *bHLH142* also leads to completely male sterile plants, although compromised pollen grains were formed in these plants. Ko *et al*.^20^ have shown that mutation in *bHLH142* results in failure of tapetal PCD and overexpression in our study results in faster tapetal PCD. This reflects a role of *bHLH142* in tapetum degeneration process. Furthermore, overexpression of *bHLH142* also affected the anther dehiscence process, which caused complete male sterility. Thus, *bHLH142* has other functions besides regulating tapteum degeneration during anther development in rice. The phenotype in overexpression transgenic rice is due to *bHLH142* as transgenics with vector alone or transgenics made using the same vector but containing other genes have been found to be fertile^44^ (our unpublished work).

During the pollen development, anther wall not only forms protective covering for developing microspore but the innermost layer of the wall, tapetum also provides nutrition to them^8^. During the release of pollen, anther wall again plays an important role and is involved in dehiscence process. Tapetum development and its degeneration have been well studied in rice^35^. However, mechanism of development and degeneration of other anther wall layers is still not clear. From this study, it is evident that *bHLH142* regulates development and degeneration of different anther wall layers. Poorly developed and fast degenerating tapetum was seen in developing anther, whereas lack of endothecium lignification, septum and stomium (modifications of epidermal cells) degeneration was observed in mature anther of *bHLH142*^*OE*^ transgenic plants.

Role of bHLH142 in the development and degeneration of anther wall layers, including endothecium and modified epidermis (stomium and septum) was identified by this study. We have shown that bHLH142 polypeptide accumulated in the epidermis, pollen and vascular tissue of the mature anther and its overexpression caused defects in secondary endothecium thickening and degeneration of the septum and stomium. Lignification of endothecium appears after the vacuolated pollen stage but the genes required for the process express a little earlier^45^. Downregulation of the various lignin metabolism-related genes in tetrad anther might be the reason for absence of lignification in *bHLH142*^*OE*^ anthers. Furthermore, defect in septum and stomium lysis may have occurred because of various reasons. In most of the plants, enzymatic lysis of septum occurs with the help of cell wall degrading enzymes like polygalacturonases (PGs), pectinases and expansins^5^,^46^,^47^. Several cell wall modification-related genes like PGs, expansins and genes encoding pectin-degrading enzymes were found to be downregulated in transgenic anthers. Furthermore, role of PCD is also suggested in septum and stomium lysis^48^ and downregulation of cell death-related genes in *bHLH142*^*OE*^ MP anther may have contributed to this phenotype. Moreover, water status in anther plays crucial role in pollen development and anther dehiscence^49^. Role of aquaporins and sugar transporters in inducing dehydration during anther dehiscence has been reported earlier^50^. We detected expression of bHLH142 protein in vascular bundle of MP anther. Four aquaporin genes and one sucrose transporter gene were found to be downregulated in MP stage of transgenic anther, which suggests that defects in water conduction might have occurred that affected anther dehiscence.

Besides regulating the anther wall functions, *bHLH142* also appeared to regulate pollen maturation process as most of the pollen grains of *bHLH142*^*OE*^plants were shrunken and non-viable. Several metabolic processes, like carbohydrate and lipid metabolism affect the pollen maturation process^39^. Complete maturation of pollen grains requires synthesis and transport of carbohydrate and lipids. Change in expression of various carbohydrate-related genes might have altered the synthesis and transport of sugars to the pollen grain, which resulted in their shrunken shape. Change in expression of sugar partitioning-related genes *MST8, INV4, UGP2, SUT3* and *AGPL1*^40^,^51^ suggests that defect in sugar loading/unloading may cause the formation of defective pollen in *bHLH142* overexpressing plants. Lipid metabolism during the pollen maturation process is an important process as deposition of lipid is required for proper pollen wall formation. Lipid transfer proteins (LTPs) are known to be involved in pollen maturation process^36^,^39^. Change in expression of various lipid metabolism-related genes and several LTPs in *bHLH142* overexpressing anther suggests that deposition of lipid on pollen wall might be altered, which contributed to the pollen abortion. *CYP704B2* and *CYP703A3*, genes encoding cytochrome P450 enzymes, catalyse hydroxylation of fatty acid to assist the pollen wall development^36^,^38^. Both of these genes were found to have altered expression in *bHLH142*^*OE*^ transgenic anther. Furthermore, *OsC4* and *OsC6*, important LTPs already known for anther development, were also differentially expressed in the *bHLH142*^*OE*^ anthers. ROS signalling has been observed to play an important role during anther cell differentiation and pollen development. Rice *MADS3* has been shown to regulate ROS homeostasis during late anther development by directly regulating expression of metallothionin gene^52^. We detected main phenotypic difference in *bHLH142*^*OE*^ plants during later stage of pollen development. Downregulation of metallothionin, peroxidases and glutathione-s-transferase genes in *bHLH142* overexpressing anthers suggests that alteration in ROS homeostasis may have contributed to defect in pollen maturation process. Therefore, it appears that *bHLH142* affects metabolic processes like carbohydrate and lipid metabolism, cell wall modification, ROS homeostasis and cell death in rice anther to control both pollen development and anther dehiscence.

**Figure 7:**
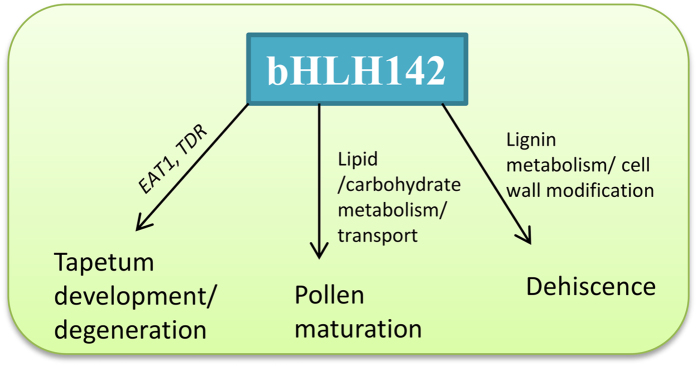
Proposed model of *bHLH142* functions during rice anther development. bHLH142 regulated various aspects of anther development in rice. It directly regulates the expression of *EAT1* and *TDR* to control tapetum development and degeneration. It affects the lipid and carbohydrate metabolism-related genes to control pollen maturation and affects lignin and cell wall modification-related genes to control anther dehiscence.

A putative model describing various functions of bHLH142 during anther development process in rice is given in Fig. 7. bHLH142 directly or indirectly affects various metabolic pathways-related genes to ensure proper anther development. It directly regulates expression of EAT1 and TDR^20^ required for the tapetum development and degeneration. Furthermore, it directly or indirectly affects the various lipid and carbohydrate metabolism-related genes required for pollen wall development and accumulation of starch during pollen maturation. It also affects the selective deposition of lignin and cell-wall degeneration-related genes required for the anther dehiscence. Therefore, bHLH142 acts in different stages of rice anther development to control different processes required for proper development and release of the pollen grains. Thus, this study has explored the novel functions of *bHLH142* in rice anther, which will contribute to the understanding of anther development process in crops to control male fertility for the hybrid seed production.

## Methods

### Plant material and growth conditions

For preparation of *bHLH142*^*OE*^ transgenic plants, ORF of the gene including flanking region of 5′ UTR was amplified from rice (variety IR 64) anther cDNA using specific primer pairs (Table S1) and by using *BamH*I and *Kpn*I restriction sites, fragment was cloned into plant binary vector pB4NU under maize ubiquitin promoter (Figure S2). Construct was then used for *Agrobacterium*-mediated rice transformation (variety Pusa Basmati1) as described by Mohanty *et al*.^53^. Transgenic plants were screened through PCR using *hygromycin phosphotransferase (hpt)* gene-specific primers (Table S1). WT and transgenic rice plants were grown in growth chamber (Conviron) maintained at 27 °C temperature, 70% relative humidity and 12 h dark/12 h light photoperiod with light intensity 220–350 μmol/m^2^s.

### Phenotypic analysis

For all the phenotypic analyses, stages of WT and transgenic spikelets/anthers were characterised as mentioned by Deveshwar *et al*.^54^ and Zhang *et al*.^1^. For histological observations, WT and transgenic spikelets of different stages were fixed in FAA solution (3.7% formaldehyde, 50% ethanol, 5% glacial acetic acid) overnight at 4 °C followed by dehydration through ethanol series and embedded into Paraplast plus X-tra (Sigma-Aldrich). Paraffin sections (8–10 μm) were cut using rotary microtome (Leica Biosystems) and stained with 0.25% toluidine blue (Sigma-Aldrich) and analysed under light microscope. In figures, cross-sections of only anther parts are shown. Endothecial lignification in anthers was studied by observing WT and transgenic anther sections in UV light under florescence microscope (Nikon-80i)^45^. For scanning electron microscopy, post-anthesis anthers were fixed into FAA solution and treated overnight with 0.2% osmium tetroxide followed by serial ethanol dehydration. The dehydrated samples were palladium coated and examined with scanning electron microscope (Evo LS25, Zeiss)^25^. Pollen viability was examined by crushing WT and transgenic anthers into I_2_-KI solution (3:1) and pollen grains were counted under light microscope^25^. Images of plants and panicles were taken with Nikon-5200 DSLR camera. All the images were processed with ImageJ software (NIH, USA).

### Immunoblotting and *in situ* immunolocalization

For immunoblotting and immunolocalization WT spikelets of different anther development stages were used. bHLH142-specific antibody was raised in rabbit against the peptide (GPFESSPTPRSGGGRKRSR). Immunoblotting and *in situ* immunolocalization was performed as previously described^32^. To check the specificity of antibody coding sequence of *EAT1, TDR* and *bHLH142* were cloned in pET28 vector and protein was expressed in *E. coli* (BL21). Immunoblotting was performed using anti-bHLH142 and anti-HIS antibody against these three proteins. To check the protein level expression of *bHLH142*, total protein from different stages of rice spikelet were used. Total protein was extracted using protein extraction buffer (20 mM Tris-HCl pH 8.0, 150 mM NaCl, 1 mM EDTA, 50 μM PMSF, 1 mM DTT, 1 mM benzimide, and 2 μg/ml aprotenin-A). It was quantified by Bradford Reagent (Sigma-Aldrich) and equal amount of the protein from different samples was electrophoresed in 10% polyacrylamide gel and blotted onto the PVDF membranes (Mdi Membrane Technologies). Membranes were first incubated either with anti-bHLH142 (1:3000 dilutions) or anti-tubulin (Sigma-Aldrich) (1:8000 dilution) primary antibody and then with HRP-conjugated anti-rabbit secondary antibody (Sigma-Aldrich) (1:5000 dilutions). Signals were detected using the ECL Plus kit (Amersham, GE Healthcare) as per manufacturer’s instructions.

Immunolocalization was performed as described by Nayar *et al*.^32^. Briefly, rice spikelets of different stages were fixed in 4% paraformaldehyde. Paraffin sections (8 μm) of the tissue were incubated in blocking buffer (1X PBS, 5% fat-free milk) for 2 h. Sections were incubated with anti-bHLH142 primary antibody (1:100 diluted in 1X PBS, 0.1% Tween20, 3% fat-free milk) overnight and with AP-conjugated anti-rabbit secondary antibody (Sigma-Aldrich) for 2 h (1:300 dilutions). NBT-BCIP solution (Amresco Inc.) was added to the samples and kept in dark condition till appearance of visible colour. Slides were mounted into DPX mounting medium (Sigma-Aldrich) and photographed under NIKON 80i microscope.

### qRT-PCR analysis

Total RNA was isolated using TRI-Reagent (Sigma-Aldrich) and purity and concentration of the RNA samples were checked by NANODROP 2000 Spectrophotometer (Thermo Scientific). cDNA for different samples were prepared with 1 μg of RNA using High-Capacity cDNA Reverse Transcription kit (Applied Biosystems). qRT-PCR analysis was performed with three biological and three technical replicates of each sample using ABI 7500 fast qRT-PCR system with the ‘Fast SYBR Green master mix’ (Applied Biosystems). Specific gene expression was normalised to *ubiquitin5 (UBQ5*) as internal control gene.

### TUNEL assay

TUNEL assay was performed as previously described^14^. Briefly, FAA-fixed tissues were used to perform TUNEL assay. Paraffin sections of WT and transgenic rice spikelets were prepared as described earlier. Sections were deparaffinised and treated with 20 mg/ml proteinase K enzyme in humid chamber for 10 min followed by PBS wash. After brief fixation of 10 min with 4% (w/v) paraformaldehyde and PBS wash, nick-end labelling of the fragmented DNA was performed using Dead End Fluorometric TUNEL system (Promega) kit according to the manufacturer’s instructions. Samples were counter stained with propidium iodide and analysed under confocal laser scanning microscope (Leica TCS SP5) with 488-nm/510-nm excitation/emission spectrum for fluorescein and a 530-nm/640-nm excitation/emission spectrum for propidium iodide.

### Subcellular localization

Nuclear localization signal (NLS) in bHLH142 protein sequence was predicted by cNLS Mapper programme. *bHLH142* CDS (Coding DNA Sequence) was fused to C-terminal sequence of YFP by cloning into pSITE3CA vector using gateway technology (Invitrogen). Empty vector and fusion constructs were transiently expressed in onion epidermal cells through biolistic bombardment-based transformation as described previously^55^. YFP fluorescence was analysed under confocal microscope (Leica TCS SP2).

### RNA-Seq analysis

Total RNA was extracted from tetrad and MP stage of WT and transgenic anthers using Tri-Reagent (Sigma-Aldrich) and purified using RNeasy MinElute CleanUp Kit (Qiagen) after DNAse I treatment. Quality and integrity of RNA was checked by NANODROP 2000 Spectrophotometer (Thermo Scientific) and Bioanalyser 2100 (Agilent Technologies). RNA sample of each genotype (WT and *bHLH142*^*OE*^) were sequenced on Illumina HiSeq platform as per manufacturer instructions. More than 60 million raw reads giving more than 12 Gb sequence data for each sample were obtained by paired-end sequencing. High quality reads were filtered and then aligned to rice gene model downloaded from RGAP ver. 7.0. Uniquely mapped reads were used for expression analysis. DESeq program^56^ was used to perform differential gene expression analysis. Count data given as input in DESeq programme were obtained using HTSeq of Python package from the alignment files. Genes showing log_2_ fold change ≥1 and p-value ≤0.05 compared to WT were treated as differentially expressed genes. GO enrichment was performed using BiNGO tool^57^ and map was visualised in Cytoscape programme. Putative function of each differentially expressed gene was obtained from RGAP Ver. 7.0.

## Additional Information

**How to cite this article:** Ranjan, R. *et al. bHLH142* regulates various metabolic pathway-related genes to affect pollen development and anther dehiscence in rice. *Sci. Rep.* **7**, 43397; doi: 10.1038/srep43397 (2017).

**Publisher's note:** Springer Nature remains neutral with regard to jurisdictional claims in published maps and institutional affiliations.

## Supplementary Material

Supplementary Information

## Figures and Tables

**Table 1:** List of previously known anther development and dehiscence-related genes showing differential expression in *bHLH142* ^*OE*^ anthers compared to WT.

Locus ID	Name	Putative Function	Log_2_ FC	p-Value	Regulation
LOC_Os01g38670.1	*MST8*	transporter family protein, putative, expressed	4.08	0.003139	Up
LOC_Os02g02560.1	*UGP2*	UTP–glucose-1-phosphate uridylyltransferase, putative	1.25	0.001296	Up
LOC_Os02g36924.1	*ZmMADS2*	OsMADS27 - MADS-box family gene with MIKCc type	−2.65	0.042858	Down
LOC_Os03g07250.1	*CYP704B2*	cytochrome P450, putative, expressed	−1.04	9.99E-11	Down
LOC_Os04g33720.1	*INV4*	glycosyl hydrolases, putative, expressed	3.71	0.00555	Up
LOC_Os04g57490.1	*CP1*	cysteine protease, putative, expressed	−3.77	0.003025	Down
LOC_Os05g50380.1	*AGPL1*	glucose-1-phosphate adenylyltransferase large subunit	−2.68	0.035567	Down
LOC_Os08g03682.1	*CYP703A3*	cytochrome P450, putative, expressed	3.22	0.039267	Up
LOC_Os08g43290.1	*OsC4*	LTPL44 - Protease inhibitor/seed storage/LTP family protein	4.57	0.011692	Up
LOC_Os09g35700.1	*LTP45*	LTPL45 - Protease inhibitor/seed storage/LTP family protein	3.88	0.010751	Up
LOC_Os10g26470.1	*SUT3*	sucrose transporter, putative, expressed	−2.60	0.040685	Down
LOC_Os10g34360.1	*YY2*	stilbene synthase, putative, expressed	4.67	0.002466	Up
LOC_Os11g37280.1	*OsC6*	LTPL68 - Protease inhibitor/seed storage/LTP family protein	2.52	0.048468	Up
