# Supplementary material for: bHLH142 regulates various metabolic pathway-related genes to affect pollen development and anther dehiscence in rice
Source: Sci Rep. 2017 Mar 6;7:43397. doi: 10.1038/srep43397 (PMC5338287; doi:10.1038/srep43397)
Supplement: Supplementary Information [file srep43397-s1.pdf]

bHLH142 regulates various metabolic pathway-related genes to affect pollen development and anther dehiscence in rice

Rajeev Ranjan[1], Reema Khurana[2], Naveen Malik[1], Saurabh Badoni[1], Swarup K. Parida[1], Sanjay Kapoor[2], Akhilesh K. Tyagi*[1, 2]

[1]National Institute of Plant Genome Research (NIPGR), Aruna Asaf Ali Marg, New Delhi 110067, India

[2]Department of Plant Molecular Biology, University of Delhi South Campus, Benito Juarez Marg, New Delhi 110021, India

***Corresponding author**

Supplementary Figures.

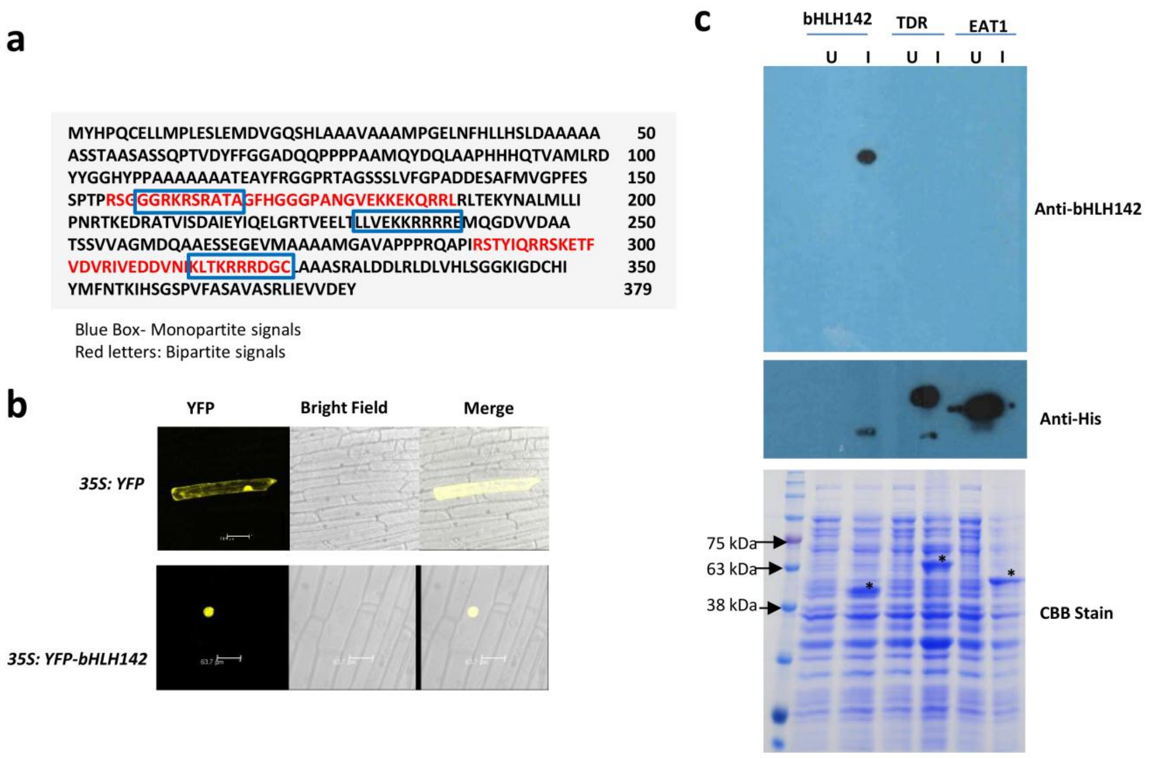

Figure S1. Nuclear localization of the bHLH142 protein and specificity of anti-bHLH142 antibody.

(a) Predicted nuclear localization signals (NLS) in the amino acid sequence. At least three monopartite and two bipartite NLS were predicated from cNLS Mapper program. **(b)** YFP fused bHLH142 protein localized exclusively into the nucleus while YFP alone distributed throughout the cell. **(c)** Immunoblot with anti-His and anti-bHLH142 antibody from cell lysate overexpressing His tagged EAT1, TDR and bHLH142 proteins. Anti-bHLH142 antibody only recognizes bHLH142 protein while anti-His antibody recognize all three proteins. Position of expressed proteins in CBB stained gel are marked (*). U, Uninduced; I Induced

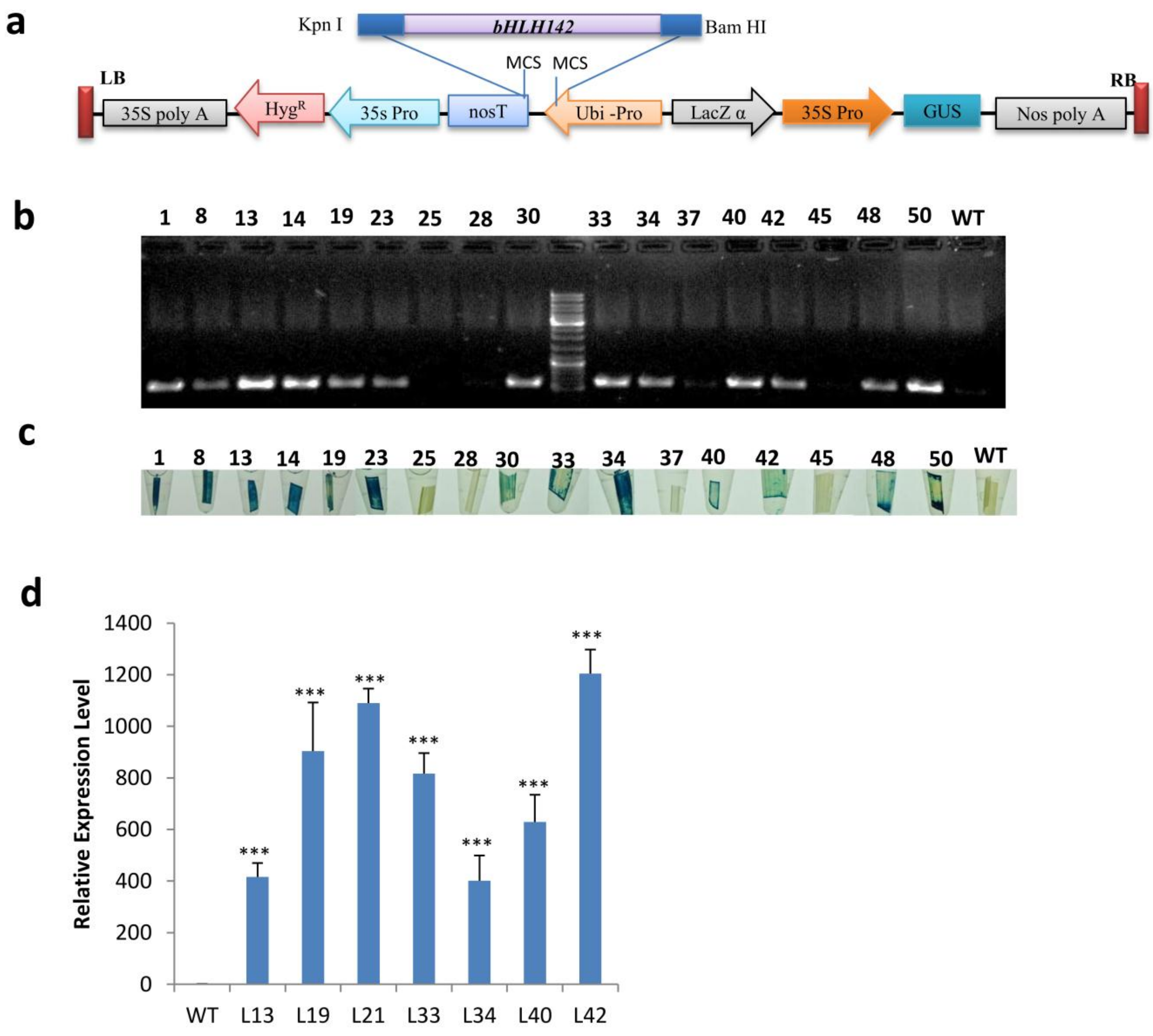

Figure S2. Generation of *bHLH142*^{OE} transgenic rice plants.

(a) Schematic representation of T-DNA region of plasmid construct used for overexpressing *bHLH142* in rice. **(b)** PCR based screening of positive transgenic plants (hygromycin resistance gene was used). **(c)** GUS reporter assay to screen positive transgenic lines. (**d**) qRT-PCR showing high accumulation of *bHLH142* transcripts in MP stage anther of positive transgenic lines. Error bars indicate standard deviation (SD). The data are presented as the mean ± SD (n=3). Asterisks indicate significant difference with respect to WT ('***' indicates *t*-test p-value ≤ 0.001).

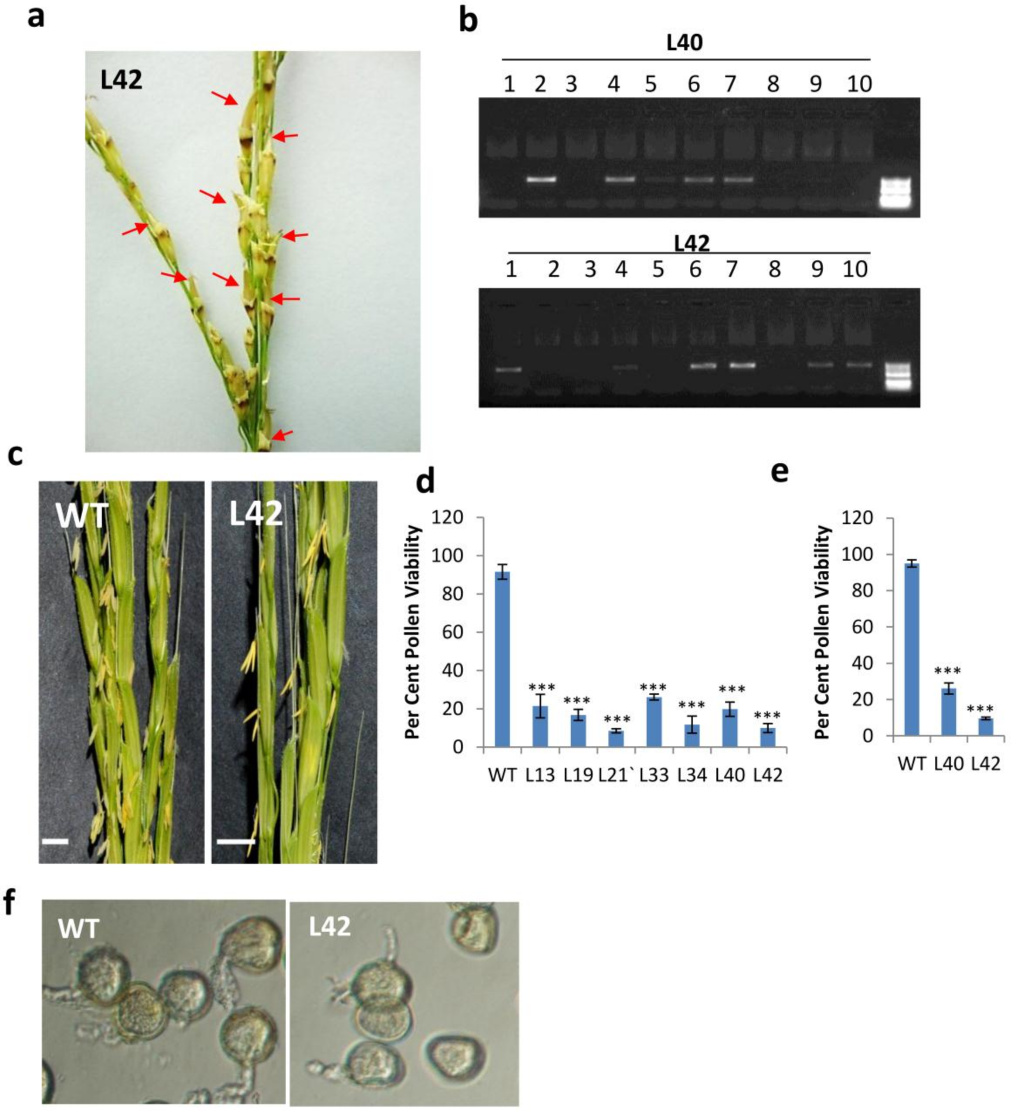

Figure S3. ***bHLH142^{OE}* plants show stable phenotype across generations. (a)** Formation of seeds when *bHLH142^{OE}* T_0 plant was hand pollinated with WT pollen. Arrows indicate formed seeds **(b)** Segregation of *bHLH142^{OE}* transgenic plant in T_1 generation. **(c)** *bHLH142^{OE}* T_2 transgenic plant showing anther indehiscence. **(d)** Per cent pollen viability of the positive T_0 transgenic lines. **(e)** Per cent pollen viability of *bHLH142^{OE}* T_2 transgenic plants. Error bars indicate standard deviation (SD). The data are presented as the mean ± SD (n=3). Asterisks indicate significant difference with respect to WT ('***' indicates *t*-test p-value ≤0.001). **(f)** *In vitro* pollen germination assay showing all viable pollen germinated normally in WT and *bHLH142^{OE}* plants. WT, Wild Type

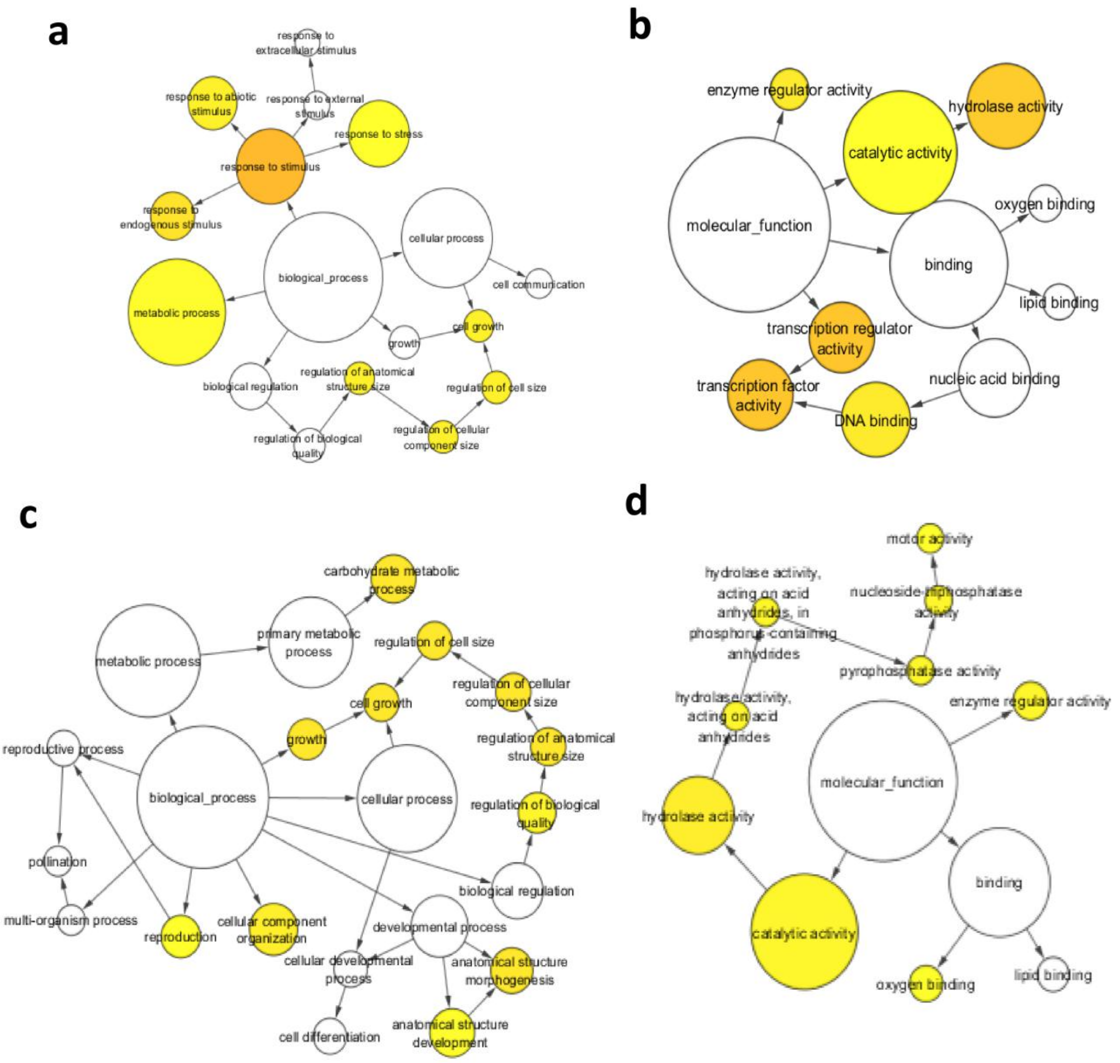

Figure S4. **Gene Ontology (GO) analysis of differentially expressed genes in *bHLH142*^{OE} anthers.** Gene ontology analysis of differentially expressed genes in tetrad **(a & b)** and mature pollen **(c & g)** stage anthers of *bHLH142*^{OE} plants. **(a)** and **(c)** are showing biological function network, while **(b)** and **(d)** show molecular function network.

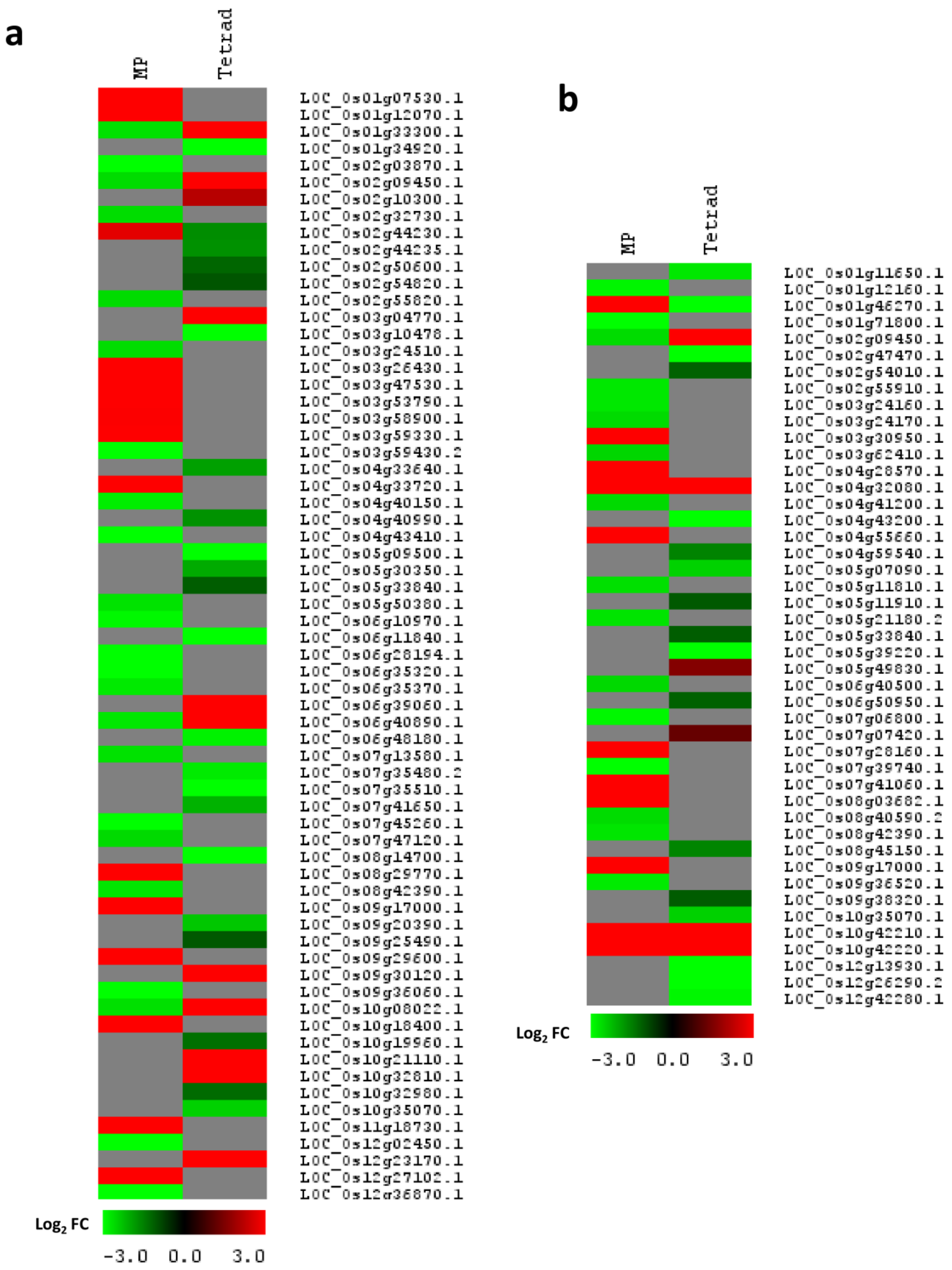

Figure S5. **Heat map showing log_2 fold change in expression of carbohydrate (a) and lipid (b) metabolism related genes in tetrad and mature pollen stages of *OsbHLH142*^{OE} anther compared to WT**

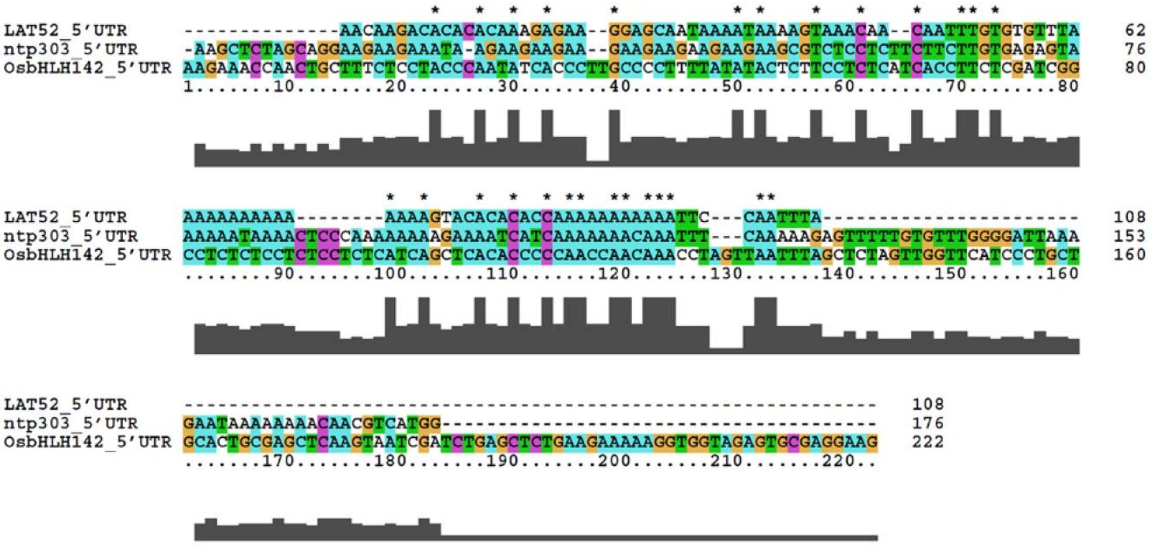

Figure S6. Sequence alignment of 5' UTR region of the *LAT52, NTP303* and *bHLH142* genes.

Supplementary table S1: List of primers used in the study			
**Primer Name**	**Forward Sequence 5'-3'**	**Reverse Sequence 5'-3'**	**Purpose**
bHLH142_cloning	GTAAGGATCCCGATCTGAGCTCTGAAGAAAAAG	CATTGGTACCTTAGTACTCATCCACCACTTC	Preparation of *OsbHLH142* ^{OE} construct
Hygro	TCTACACAGCCATGCGTCCAG	GATGTAGGAGGGCGTGGATATG	Screening of positive transgenic plants
UBQ5 RT	ACCACTTCGACCGCCACTAC	ACGCCTAAGCCTGCTGGTT	qRT-PCR
bHLH142 RT	TCGGCGACTGCCACATCTA	GCACTTGCAAACACTGGAGATC	qRT-PCR
bHLH142 Topo	CACCATGTATCACCCGCAGTGC	TTAGTACTCATCCACCACTTC	Subcellular localization
bHLH142_Bact	CACCCATATGATGTATCACCCGCAGTGC	CACCGGATCCTTAGTACTCATCCACCACTTC	Bacterial expression
TDR_Bact	CACCCATATGATGGGAAGAGGAGACCACCT	CACCGGATCCTCAATCAAACGCGAGGTAATGC	Bacterial expression
EAT1_Bact	CACCCATATGATGATTGTTGGGGCTGGTTA	CACCGAATTCTTA GTTGAATATGTCGAGGGCCTGG	Bacterial expression
LTP45_RT	TCACCTGCCAGTGATGGAGAT	CATCCTCGCACGAGCAAAC	qRT-PCR
OsC6_RT	CTTGCCCGGCTCCATCT	TGAAATCCCTCCTTTGGTAACATT	qRT-PCR
Os06g35320_RT	CGAGAAGGATGTGACGGATGT	GCCATTGGTGGACTTCTTGAG	qRT-PCR
Os06g5260_RT	GCGACGACATGAAGCTCAAG	CTTGTACTCGTCGTAGCTCGTGTAC	qRT-PCR
Os06g35520_RT	GCCCTGGGAACCAATGC	TCACGTTTCGTCTCTTCTTGTGTT	qRT-PCR
Os06g46799_RT	CCTCGTCAACGACATCCACTAC	AAGCACCGAGGAGCTGAAGA	qRT-PCR
Os04g14710_RT	CCCGAGGAGGAAGAATGGAT	ATCAACAGGGCCATGAACGT	qRT-PCR
Os0441680q_RT	GACCGCACCAACGATGACT	TGAGTGACGTGGGCAAAGAA	qRT-PCR

Supplementary table S2: Differentially expressed genes in *bHLH142* ^{OE} tetrad anthers compared to wild type (log_2 fold change ≥ 1.0 and P value ≥ 0.05).

Gene_ID	Putative Functions	log_2 fold change	P-value	Regulation
LOC_Os01g37000.1	carboxyl-terminal peptidase, putative, expressed	-11.37966593	2.13E-81	Down
LOC_Os08g08970.1	Cupin domain containing protein, expressed	-11.03522039	1.22E-66	Down
LOC_Os11g37280.1	LTPL68 - Protease inhibitor/seed storage/LTP family protein precursor, expressed	-10.0801786	1.55E-166	Down
LOC_Os09g17560.1	O-methyltransferase, putative, expressed	-8.86107461	1.36E-16	Down
LOC_Os07g45250.1	transposon protein, putative, unclassified, expressed	-8.36064562	4.57E-41	Down
LOC_Os01g24710.2	jacalin-like lectin domain containing protein, expressed	-8.312948878	3.69E-56	Down
LOC_Os04g48210.1	cytochrome P450, putative, expressed	-8.041130347	4.60E-97	Down
LOC_Os12g44010.1	purple acid phosphatase precursor, putative, expressed	-7.800310091	8.22E-22	Down
LOC_Os01g41170.1	THION27 - Plant thionin family protein precursor, expressed	-7.478827046	1.14E-72	Down
LOC_Os08g07100.1	terpene synthase, putative, expressed	-7.144449465	2.74E-05	Down
LOC_Os12g13930.1	3-oxoacyl-reductase, chloroplast precursor, putative, expressed	-7.082212332	1.97E-53	Down
LOC_Os05g30580.1	OsSub46 - Putative Subtilisin homologue, expressed	-7.065377894	5.03E-05	Down
LOC_Os01g03390.1	BBTI7 - Bowman-Birk type bran trypsin inhibitor precursor, expressed	-6.768160283	1.66E-45	Down
LOC_Os09g23620.1	MYB family transcription factor, putative, expressed	-6.721374646	2.38E-24	Down
LOC_Os01g48570.1	expressed protein	-6.68759179	3.94E-21	Down
LOC_Os04g27670.1	terpene synthase family, metal binding domain containing protein, expressed	-6.680028495	5.14E-21	Down
LOC_Os12g44270.1	glycine-rich protein, putative, expressed	-6.643551229	0.00075841	Down
LOC_Os07g37920.1	no apical meristem protein, putative, expressed	-6.559486964	0.00118784	Down
LOC_Os04g16736.1	photosystem I assembly protein ycf3, putative, expressed	-6.535683939	4.22E-28	Down
LOC_Os04g56430.1	cysteine-rich receptor-like protein kinase, putative, expressed	-6.515543616	0.0014858	Down
LOC_Os11g24060.1	permease domain containing protein, putative, expressed	-6.454789585	0.00200132	Down
LOC_Os02g42800.1	expressed protein	-6.35857427	0.00312437	Down
LOC_Os05g39220.1	GDSL-like lipase/acylhydrolase, putative, expressed	-6.32502171	0.00362305	Down
LOC_Os11g47809.1	metallothionein, putative, expressed	-6.312235555	4.60E-18	Down
LOC_Os11g45560.1	MBTB68 - Bric-a-Brac, Tramtrack, Broad Complex BTB domain with Meprin and TRAF H	-6.290670205	0.00420044	Down
LOC_Os06g13460.1	SAM dependent carboxyl methyltransferase family protein, putative, expressed	-6.191755179	5.05E-05	Down
LOC_Os01g50330.1	strictosidine synthase 1 precursor, putative, expressed	-6.188649268	4.99E-07	Down
LOC_Os03g05900.1	expressed protein	-6.163558287	6.26E-05	Down
LOC_Os04g49748.1	purine permease, putative, expressed	-6.163558287	0.00703537	Down
LOC_Os04g41680.1	CHIT3 - Chitinase family protein precursor, expressed	-5.892910698	0.01817455	Down

Supplementary table S2 continued..

Gene_ID	Putative Functions	log_2 fold change	P-value	Regulation
LOC_Os11g32650.1	chalcone synthase, putative, expressed	-5.856468702	1.89E-11	Down
LOC_Os10g19160.1	receptor kinase, putative, expressed	-5.846368112	0.02100651	Down
LOC_Os12g03040.1	no apical meristem protein, putative, expressed	-5.818142929	7.76E-42	Down
LOC_Os03g62840.1	expressed protein	-5.798273823	0.02427084	Down
LOC_Os04g40470.1	cytochrome P450, putative, expressed	-5.773611769	0.02608481	Down
LOC_Os09g10054.1	disease resistance protein RPS2, putative, expressed	-5.773611769	0.02608481	Down
LOC_Os04g19750.1	OsFBL11 - F-box domain and LRR containing protein, expressed	-5.740059209	3.80E-05	Down
LOC_Os06g14350.1	caleosin related protein, putative, expressed	-5.699087428	1.76E-30	Down
LOC_Os03g51350.1	expressed protein	-5.696990488	0.03236184	Down
LOC_Os01g52200.1	mitochondrial ATP synthase g subunit family protein, putative, expressed	-5.643551229	0.00163228	Down
LOC_Os06g35520.1	peroxidase precursor, putative, expressed	-5.632164468	1.97E-15	Down
LOC_Os07g47410.1	WD40-like domain containing protein, putative, expressed	-5.602131301	0.00201167	Down
LOC_Os01g62430.1	C2 domain containing protein, putative, expressed	-5.584009283	2.44E-09	Down
LOC_Os08g33150.1	MYB family transcription factor, putative, expressed	-5.573161901	6.81E-24	Down
LOC_Os09g21660.1	expressed protein	-5.559486964	0.04625464	Down
LOC_Os04g27430.1	terpene synthase, putative, expressed	-5.559486964	0.00247779	Down
LOC_Os07g39640.1	LTPL64 - Protease inhibitor/seed storage/LTP family protein precursor, expressed	-5.549836794	1.42E-17	Down
LOC_Os08g24140.1	OsFBL47 - F-box domain and LRR containing protein, expressed	-5.32502171	0.00695674	Down
LOC_Os11g03300.1	NAC domain transcription factor, putative, expressed	-5.259161123	3.39E-43	Down
LOC_Os07g14750.1	60S acidic ribosomal protein, putative, expressed	-5.163558287	0.0128083	Down
LOC_Os09g21670.1	expressed protein	-5.144449465	0.00027005	Down
LOC_Os09g11480.2	AP2 domain containing protein, expressed	-5.12508414	0.01465333	Down
LOC_Os03g57000.1	DNA topoisomerase IV subunit A, putative, expressed	-5.12508414	0.00030763	Down
LOC_Os09g29700.1	retrotransposon protein, putative, unclassified, expressed	-5.109402517	5.43E-05	Down
LOC_Os04g53606.1	expressed protein	-5.085555775	0.00251371	Down
LOC_Os02g18670.1	ABC transporter family protein, putative, expressed	-5.065377894	0.01791676	Down
LOC_Os10g38470.1	glutathione S-transferase, putative, expressed	-5.058588728	0.00286652	Down
LOC_Os05g35400.1	DnaK family protein, putative, expressed	-5.003093615	0.00372409	Down
LOC_Os05g11580.1	expressed protein	-4.981719965	0.02338894	Down
LOC_Os06g41800.1	dihydroflavonol-4-reductase, putative, expressed	-4.960024893	0.00017131	Down
LOC_Os08g03070.1	lectin-like receptor kinase 1, putative, expressed	-4.937998587	0.00098322	Down
LOC_Os11g10910.1	chloroplast nucleoid DNA-binding protein, putative, expressed	-4.902041709	3.96E-29	Down
LOC_Os03g61290.1	ATCHX, putative, expressed	-4.892910698	0.00126928	Down

Supplementary table S2 continued..

Gene_ID	Putative Functions	log_2 fold change	P-value	Regulation
LOC_Os01g02190.1	aquaporin protein, putative, expressed	-4.869827084	4.80E-26	Down
LOC_Os02g54690.1	RNA recognition motif containing protein, putative, expressed	-4.869827084	0.00667742	Down
LOC_Os10g40859.1	matrixin family protein, expressed	-4.82252137	0.03711289	Down
LOC_Os01g06360.1	transposon protein, putative, unclassified, expressed	-4.798273823	0.03962221	Down
LOC_Os07g48550.1	no apical meristem protein, putative, expressed	-4.77005395	2.56E-34	Down
LOC_Os06g11840.1	trehalose phosphatase, putative, expressed	-4.728699347	4.80E-06	Down
LOC_Os02g10450.1	expressed protein	-4.722985696	0.04817323	Down
LOC_Os02g36020.1	transposon protein, putative, CACTA, En/Spm sub-class, expressed	-4.702227136	0.00092903	Down
LOC_Os01g45400.1	ubiquitin family protein, putative, expressed	-4.662864704	2.98E-08	Down
LOC_Os09g31019.1	ubiquitin fusion protein, putative, expressed	-4.616070492	1.76E-07	Down
LOC_Os11g14900.4	thiol protease SEN102 precursor, putative, expressed	-4.587247657	9.09E-17	Down
LOC_Os05g49540.1	retrotransposon protein, putative, Ty1-copia subclass, expressed	-4.559486964	0.0209568	Down
LOC_Os03g55410.1	peroxidase precursor, putative, expressed	-4.546605673	2.84E-05	Down
LOC_Os07g39320.1	homeobox domain containing protein, expressed	-4.526128367	0.00028132	Down
LOC_Os05g15770.1	glycosyl hydrolase, putative, expressed	-4.435267027	3.15E-19	Down
LOC_Os04g51796.1	DNA repair ATPase-related, putative, expressed	-4.402896601	0.00064255	Down
LOC_Os06g20960.1	SAM dependent carboxyl methyltransferase, putative, expressed	-4.396757464	0.03441291	Down
LOC_Os04g19800.1	F-box domain containing protein, expressed	-4.391364205	0.01266431	Down
LOC_Os12g24020.1	rhodanese-like domain containing protein, putative, expressed	-4.307948197	0.04395025	Down
LOC_Os12g37519.2	retrotransposon protein, putative, unclassified, expressed	-4.296824818	6.98E-12	Down
LOC_Os08g34890.1	OsFBDUF46 - F-box and DUF domain containing protein, expressed	-4.280203206	0.0076872	Down
LOC_Os06g24990.1	xylanase inhibitor protein 1 precursor, putative, expressed	-4.280203206	0.0076872	Down
LOC_Os03g17870.1	metallothionein, putative, expressed	-4.253500413	0.00030252	Down
LOC_Os04g45130.1	heavy-metal-associated domain-containing protein, putative, expressed	-4.219411522	0.02302734	Down
LOC_Os06g02360.1	Skp1 family, dimerisation domain containing protein, expressed	-4.217750388	8.16E-05	Down
LOC_Os01g18110.1	cinnamoyl CoA reductase, putative, expressed	-4.17866518	0.01157207	Down
LOC_Os07g35510.1	glucan endo-1,3-beta-glucosidase precursor, putative, expressed	-4.152669971	1.60E-05	Down
LOC_Os08g14700.1	glucan endo-1,3-beta-glucosidase precursor, putative, expressed	-4.085555775	0.00784121	Down
LOC_Os04g30030.1	cysteine-rich receptor-like protein kinase 12 precursor, putative, expressed	-4.056111998	0.00028875	Down
LOC_Os03g61150.1	expressed protein	-4.045829497	4.43E-13	Down
LOC_Os04g13040.1	OsFBX124 - F-box domain containing protein, expressed	-4.024155231	0.04132669	Down
LOC_Os03g12700.1	expressed protein	-3.933552682	0.02727694	Down
LOC_Os03g20870.1	zinc finger, C3HC4 type domain containing protein, expressed	-3.928454814	0.00783932	Down

Supplementary table S2 continued..

Gene_ID	Putative Functions	log_2 fold change	P-value	Regulation
LOC_Os08g34860.1	OsFBX293 - F-box domain containing protein, expressed	-3.880526973	0.0003102	Down
LOC_Os01g10400.1	expressed protein	-3.87118194	2.65E-48	Down
LOC_Os01g59640.1	no apical meristem protein, putative, expressed	-3.86048922	0.03411667	Down
LOC_Os01g14430.1	DNA binding protein, putative, expressed	-3.854230229	0.01915443	Down
LOC_Os04g32200.1	expressed protein	-3.851090522	0.00198468	Down
LOC_Os05g46020.1	WRKY7, expressed	-3.849742838	0.01087769	Down
LOC_Os02g44990.1	OsFBDUF13 - F-box and DUF domain containing protein, expressed	-3.80871557	0.01279492	Down
LOC_Os01g71670.1	glycosyl hydrolases family 17, putative, expressed	-3.803156045	0.04028982	Down
LOC_Os10g33760.1	No apical meristem protein, putative, expressed	-3.790099892	6.33E-07	Down
LOC_Os04g57490.1	cysteine protease, putative, expressed	-3.773611769	0.00302483	Down
LOC_Os02g48870.1	aspartic proteinase nepenthesin-2 precursor, putative, expressed	-3.729034741	6.57E-08	Down
LOC_Os01g64470.1	harpin-induced protein 1 domain containing protein, expressed	-3.722985696	0.02959503	Down
LOC_Os04g32620.1	ethylene-responsive transcription factor ERF114, putative, expressed	-3.700732889	0.00066369	Down
LOC_Os08g30510.1	expressed protein	-3.667971594	6.78E-09	Down
LOC_Os05g33400.1	basic 7S globulin precursor, putative, expressed	-3.662864704	0.02181735	Down
LOC_Os04g42550.1	expressed protein	-3.65471642	0.00024105	Down
LOC_Os05g02760.1	expressed protein	-3.593702679	0.00011321	Down
LOC_Os01g03330.1	BBTI3 - Bowman-Birk type bran trypsin inhibitor precursor, expressed	-3.592763119	2.32E-05	Down
LOC_Os10g28810.1	MBTB37 - Bric-a-Brac, Tramtrack, Broad Complex BTB domain with Meprin and TRAF H	-3.591195824	0.01137465	Down
LOC_Os11g37970.1	WIP5 - Wound-induced protein precursor, expressed	-3.559486964	0.00846743	Down
LOC_Os12g26290.2	alpha-DOX2, putative, expressed	-3.55233685	9.94E-15	Down
LOC_Os02g52930.1	integral membrane protein DUF6 containing protein, expressed	-3.552255395	0.00076673	Down
LOC_Os02g45160.1	aluminum-activated malate transporter, putative, expressed	-3.540121639	0.01394808	Down
LOC_Os08g41780.1	triacylglycerol lipase precursor, putative, expressed	-3.535081651	3.16E-06	Down
LOC_Os03g10478.1	glycosyl hydrolase family 10 protein, putative, expressed	-3.521351835	6.77E-05	Down
LOC_Os01g66120.1	No apical meristem protein, putative, expressed	-3.475892834	3.46E-09	Down
LOC_Os09g35030.1	dehydration-responsive element-binding protein, putative, expressed	-3.45328756	8.25E-06	Down
LOC_Os04g59200.1	peroxidase precursor, putative, expressed	-3.430203947	0.04508264	Down
LOC_Os12g03790.1	rhamnogalacturonate lyase, putative, expressed	-3.426592693	0.00042923	Down
LOC_Os03g09830.2	expressed protein	-3.42198344	0.01036391	Down
LOC_Os09g22530.1	expressed protein	-3.393678071	0.04989583	Down
LOC_Os10g36210.1	valyl-tRNA synthetase, putative, expressed	-3.388118546	0.01700805	Down
LOC_Os03g49630.1	expressed protein	-3.366841886	0.00242438	Down

Supplementary table S2 continued..

Gene_ID	Putative Functions	log_2 fold change	P-value	Regulation
LOC_Os02g49860.1	AWPM-19-like membrane family protein, putative, expressed	-3.361887004	0.01875761	Down
LOC_Os09g07450.1	flavonol synthase, putative, expressed	-3.341895529	0.04023451	Down
LOC_Os05g09500.1	hexokinase, putative, expressed	-3.339515934	4.75E-16	Down
LOC_Os04g43200.1	caleosin related protein, putative, expressed	-3.323715513	0.00804504	Down
LOC_Os06g10750.1	integral membrane protein DUF6 containing protein, expressed	-3.258022971	0.00014365	Down
LOC_Os11g47600.1	glycosyl hydrolase, putative, expressed	-3.227216925	0.00914552	Down
LOC_Os10g05720.1	LTPL37 - Protease inhibitor/seed storage/LTP family protein precursor, expressed	-3.219411522	0.00058376	Down
LOC_Os04g34050.1	VQ domain containing protein, putative, expressed	-3.211086658	0.02347763	Down
LOC_Os02g21009.2	sodium/calcium exchanger protein, putative, expressed	-3.191755179	0.00612709	Down
LOC_Os08g09770.1	DnaK family protein, putative, expressed	-3.188649268	0.00360229	Down
LOC_Os05g50210.1	expressed protein	-3.188649268	0.04558586	Down
LOC_Os03g49440.1	phosphatase, putative, expressed	-3.144002048	1.02E-05	Down
LOC_Os02g45450.1	dehydration-responsive element-binding protein, putative, expressed	-3.135679255	6.63E-08	Down
LOC_Os10g04800.1	expressed protein	-3.12508414	0.0004264	Down
LOC_Os03g10210.1	homeobox domain containing protein, expressed	-3.100967834	8.04E-37	Down
LOC_Os05g49900.1	3-ketoacyl-CoA synthase, putative, expressed	-3.097834105	4.22E-05	Down
LOC_Os10g35460.1	COBRA, putative, expressed	-3.076095446	0.00821754	Down
LOC_Os09g35020.1	AP2 domain containing protein, expressed	-2.980858397	1.84E-09	Down
LOC_Os01g72530.1	OsCML31 - Calmodulin-related calcium sensor protein, expressed	-2.958122856	4.18E-05	Down
LOC_Os08g40680.1	glycosyl hydrolase, putative, expressed	-2.945378117	0.04329426	Down
LOC_Os03g61500.1	uncharacterized Cys-rich domain containing protein, putative, expressed	-2.920631455	0.00038953	Down
LOC_Os12g42280.1	9-cis-epoxycarotenoid dioxygenase 1, chloroplast precursor, putative, expressed	-2.910999325	0.00024145	Down
LOC_Os09g22460.1	OsFBX328 - F-box domain containing protein, expressed	-2.907410267	0.00625724	Down
LOC_Os11g10920.1	carboxyl-terminal proteinase, putative, expressed	-2.905261801	1.49E-14	Down
LOC_Os02g42585.1	AP2 domain containing protein, expressed	-2.898352783	0.0079681	Down
LOC_Os12g26510.1	expressed protein	-2.890827383	0.00680145	Down
LOC_Os02g53440.1	expressed protein	-2.889635566	0.03364889	Down
LOC_Os04g32850.1	basic proline-rich protein, putative, expressed	-2.887616398	0.04164664	Down
LOC_Os08g37660.1	plastocyanin-like domain containing protein, putative, expressed	-2.86118299	2.84E-05	Down
LOC_Os06g48180.1	glycosyl hydrolases family 16, putative, expressed	-2.856468702	6.89E-11	Down
LOC_Os03g21030.1	no apical meristem protein, putative, expressed	-2.855209988	5.90E-06	Down
LOC_Os09g10260.1	Skp1 family, dimerisation domain containing protein, expressed	-2.826079189	0.00381518	Down
LOC_Os02g08440.1	WRKY71, expressed	-2.82322324	2.32E-10	Down

Supplementary table S2 continued..

Gene_ID	Putative Functions	log_2 fold change	P-value	Regulation
LOC_Os07g05180.1	Skp1 family, dimerisation domain containing protein, expressed	-2.811794963	0.00089493	Down
LOC_Os06g12320.1	transmembrane amino acid transporter protein, putative, expressed	-2.796331846	1.42E-05	Down
LOC_Os07g35480.2	glucan endo-1,3-beta-glucosidase precursor, putative, expressed	-2.767379815	0.00233064	Down
LOC_Os01g14440.1	WRKY1, expressed	-2.763627681	0.00748962	Down
LOC_Os01g45430.1	expressed protein	-2.756933028	0.02519295	Down
LOC_Os01g52130.1	sulfate transporter, putative, expressed	-2.752132042	6.59E-05	Down
LOC_Os11g45740.1	MYB family transcription factor, putative, expressed	-2.736511061	5.98E-07	Down
LOC_Os01g11650.1	GDSL-like lipase/acylhydrolase, putative, expressed	-2.735237957	1.63E-05	Down
LOC_Os03g46060.1	thaumatin family domain containing protein, expressed	-2.722985696	0.0398603	Down
LOC_Os02g52934.1	expressed protein	-2.71004664	0.00330586	Down
LOC_Os01g50410.1	STE_MEKK_ste11_MAP3K.6 - STE kinases include homologs to sterile 7, sterile 11 and	-2.701612045	2.11E-05	Down
LOC_Os02g12700.1	interferon-related developmental regulator family protein, putative, expressed	-2.698532652	0.03655012	Down
LOC_Os02g41840.1	DUF584 domain containing protein, putative, expressed	-2.651060769	0.00058511	Down
LOC_Os01g41140.1	THION18 - Plant thionin family protein precursor, expressed	-2.613100149	1.24E-17	Down
LOC_Os03g50960.1	LTPL118 - Protease inhibitor/seed storage/LTP family protein precursor, expressed	-2.603686768	0.04263895	Down
LOC_Os08g28820.1	Skp1 family, dimerisation domain containing protein, expressed	-2.587008027	0.01285437	Down
LOC_Os03g05610.1	inorganic phosphate transporter, putative, expressed	-2.570982603	0.03572134	Down
LOC_Os05g37250.1	cytochrome P450, putative, expressed	-2.55792645	5.05E-05	Down
LOC_Os11g02290.1	expressed protein	-2.554532082	0.00533671	Down
LOC_Os07g27300.2	RNA-binding protein Luc7-like, putative, expressed	-2.537236757	8.74E-19	Down
LOC_Os04g52440.1	aminotransferase, putative, expressed	-2.530292271	2.29E-22	Down
LOC_Os04g23550.1	basic helix-loop-helix family protein, putative, expressed	-2.524977433	1.60E-05	Down
LOC_Os04g46940.1	copper-transporting ATPase 3, putative, expressed	-2.521174152	0.00043889	Down
LOC_Os04g35860.1	T-complex protein 11, putative, expressed	-2.515860031	0.00160338	Down
LOC_Os01g49820.2	lipid phosphatase protein, putative, expressed	-2.510577363	0.02614947	Down
LOC_Os01g17330.2	eukaryotic translation initiation factor 6, putative, expressed	-2.481977597	0.02282293	Down
LOC_Os07g05365.1	photosystem II 10 kDa polypeptide, chloroplast precursor, putative, expressed	-2.477229282	4.69E-05	Down
LOC_Os05g07090.1	acyl-coenzyme A dehydrogenase, mitochondrial precursor, putative, expressed	-2.476942007	5.21E-05	Down
LOC_Os10g35070.1	alpha-galactosidase precursor, putative, expressed	-2.470219626	0.01181937	Down
LOC_Os02g45420.1	AP2 domain containing protein, expressed	-2.466804961	0.00760529	Down
LOC_Os01g47760.1	OsGrx_I1 - glutaredoxin subgroup III, expressed	-2.461912807	0.00042403	Down
LOC_Os07g39210.1	expressed protein	-2.461064911	0.00700425	Down
LOC_Os05g38040.1	expressed protein	-2.447978649	7.64E-11	Down

Supplementary table S2 continued..

Gene_ID	Putative Functions	log_2 fold change	P-value	Regulation
LOC_Os03g47420.1	OsFBX107 - F-box domain containing protein, expressed	-2.445250241	0.0148677	Down
LOC_Os02g33070.1	expressed protein	-2.444009746	0.02646675	Down
LOC_Os04g48290.1	MATE efflux family protein, putative, expressed	-2.431754398	9.08E-09	Down
LOC_Os01g08800.1	cytochrome P450, putative, expressed	-2.427383428	0.00051432	Down
LOC_Os06g21240.1	glycine rich protein family protein, putative, expressed	-2.42549182	0.0400253	Down
LOC_Os02g52010.1	phosphate-induced protein 1 conserved region domain containing protein, expressed	-2.414311933	2.48E-08	Down
LOC_Os01g21250.1	late embryogenesis abundant protein, putative, expressed	-2.4120566	0.0239151	Down
LOC_Os03g10950.1	protein phosphatase 2C, putative, expressed	-2.40748387	0.00133252	Down
LOC_Os07g30150.1	phosphoribosyl transferase, putative, expressed	-2.406377933	0.02192532	Down
LOC_Os08g34820.1	OsFBX292 - F-box domain containing protein, expressed	-2.385950709	0.00340749	Down
LOC_Os11g39780.1	expressed protein	-2.375062393	0.01478925	Down
LOC_Os01g58140.1	expressed protein	-2.366841886	3.26E-06	Down
LOC_Os09g20390.1	uncharacterized glycosyl hydrolase Rv2006/MT2062, putative, expressed	-2.34222123	3.58E-14	Down
LOC_Os06g48500.1	expressed protein	-2.311759811	0.00065206	Down
LOC_Os05g46760.1	STE_MEKK_ste11_MAP3K.19 - STE kinases include homologs to sterile 7, sterile 11 an[illegible]	-2.311215911	8.37E-06	Down
LOC_Os05g27730.1	WRKY53, expressed	-2.309464429	2.28E-11	Down
LOC_Os02g10520.1	OsSub12 - Putative Subtilisin homologue, expressed	-2.308315528	1.00E-07	Down
LOC_Os03g15270.1	gibberellin receptor GID1L2, putative, expressed	-2.278200853	0.03940436	Down
LOC_Os08g04140.1	X8 domain containing protein, expressed	-2.235403984	1.30E-05	Down
LOC_Os08g10500.1	expressed protein	-2.234550119	0.0001984	Down
LOC_Os01g62490.1	laccase precursor protein, putative, expressed	-2.212023777	0.00456445	Down
LOC_Os01g31370.1	glycosyltransferase, putative, expressed	-2.207985754	0.0018675	Down
LOC_Os04g26550.1	expressed protein	-2.204200272	0.02791357	Down
LOC_Os03g12890.1	aminotransferase domain containing protein, putative, expressed	-2.194490147	0.03150648	Down
LOC_Os01g09220.1	transposon protein, putative, CACTA, En/Spm sub-class, expressed	-2.184315646	7.07E-28	Down
LOC_Os09g35010.1	dehydration-responsive element-binding protein, putative, expressed	-2.181771091	1.51E-10	Down
LOC_Os12g02080.1	peroxidase precursor, putative, expressed	-2.179843371	0.00874151	Down
LOC_Os05g09020.1	WRKY67, expressed	-2.158487298	0.04972876	Down
LOC_Os06g48020.1	peroxidase precursor, putative, expressed	-2.14736106	0.04066334	Down
LOC_Os01g50420.1	STE_MEKK_ste11_MAP3K.7 - STE kinases include homologs to sterile 7, sterile 11 and	-2.142496672	0.0029634	Down
LOC_Os03g55540.1	ZOS3-18 - C2H2 zinc finger protein, expressed	-2.116639325	7.63E-09	Down
LOC_Os04g48350.1	dehydration-responsive element-binding protein, putative, expressed	-2.113900401	0.00022736	Down
LOC_Os07g41650.1	pectinesterase, putative, expressed	-2.103257777	0.00018695	Down

Supplementary table S2 continued..

Gene_ID	Putative Functions	log_2 fold change	P-value	Regulation
LOC_Os01g09640.1	Myb transcription factor, putative, expressed	-2.102716327	0.0050435	Down
LOC_Os06g02490.1	acyl CoA binding protein, putative, expressed	-2.093316392	0.00048203	Down
LOC_Os05g04500.1	peroxidase precursor, putative, expressed	-2.09107278	0.0013215	Down
LOC_Os08g38170.1	methyladenine glycosylase, putative, expressed	-2.085555775	0.02004547	Down
LOC_Os07g46210.1	LTPL2 - Protease inhibitor/seed storage/LTP family protein precursor, expressed	-2.0663783	5.45E-25	Down
LOC_Os04g51460.1	glycosyl hydrolases family 16, putative, expressed	-2.063004542	8.45E-11	Down
LOC_Os12g08130.1	amino acid transporter, putative, expressed	-2.058065932	2.43E-14	Down
LOC_Os03g08500.2	AP2 domain containing protein, expressed	-2.051883346	3.09E-11	Down
LOC_Os10g25290.1	ZIM domain containing protein, putative, expressed	-2.044259359	0.0002059	Down
LOC_Os01g40070.1	expressed protein	-2.033551167	9.24E-13	Down
LOC_Os07g46852.1	sex determination protein tasselseed-2, putative, expressed	-2.03320037	0.01612803	Down
LOC_Os06g46950.1	EF hand family protein, putative, expressed	-2.02369052	7.13E-15	Down
LOC_Os03g53020.1	helix-loop-helix DNA-binding domain containing protein, expressed	-2.023434064	0.01240485	Down
LOC_Os05g30350.1	Os5bglu22 - beta-glucosidase homologue, similar to G. max isohydroxyurate hydrolase	-2.019060364	0.04792078	Down
LOC_Os02g49030.1	tyrosyl-tRNA synthetase, putative, expressed	-2.007800618	0.00587546	Down
LOC_Os10g31320.1	retrotransposon protein, putative, unclassified, expressed	-1.994113784	1.69E-06	Down
LOC_Os08g24820.1	expressed protein	-1.992706997	0.00366892	Down
LOC_Os01g55510.1	dynein light chain type 1 domain containing protein, expressed	-1.979577691	4.36E-05	Down
LOC_Os01g21120.1	AP2 domain containing protein, expressed	-1.976293556	6.65E-05	Down
LOC_Os02g47470.1	cytochrome P450, putative, expressed	-1.968946589	7.12E-15	Down
LOC_Os04g33820.1	OsFBX132 - F-box domain containing protein, expressed	-1.955159138	0.00013696	Down
LOC_Os07g37100.1	nucleoside transporter, putative, expressed	-1.953153026	9.04E-09	Down
LOC_Os01g38980.1	calmodulin-binding protein, putative, expressed	-1.95302102	7.19E-06	Down
LOC_Os09g13650.1	microtubule-associated protein, putative, expressed	-1.950340428	0.00013797	Down
LOC_Os02g13510.1	receptor-like protein kinase 5 precursor, putative, expressed	-1.939166289	0.00716491	Down
LOC_Os11g02820.1	CRP10 - Cysteine-rich family protein precursor, expressed	-1.928230811	0.02098586	Down
LOC_Os04g45340.1	expressed protein	-1.926858029	0.01448887	Down
LOC_Os01g63690.1	hs1, putative, expressed	-1.922942979	3.74E-06	Down
LOC_Os07g48280.1	expressed protein	-1.919939473	1.82E-15	Down
LOC_Os10g28240.1	calcium-transporting ATPase, plasma membrane-type, putative, expressed	-1.907218534	0.00526083	Down
LOC_Os08g14360.1	expressed protein	-1.895822293	0.02702272	Down
LOC_Os04g33640.1	glycosyl hydrolases family 17, putative, expressed	-1.883596946	0.0003694	Down
LOC_Os05g31140.1	glycosyl hydrolases family 17, putative, expressed	-1.88246391	0.0056085	Down

Supplementary table S2 continued..

Gene_ID	Putative Functions	log_2 fold change	P-value	Regulation
LOC_Os03g43720.3	transporter family protein, putative, expressed	-1.879880749	1.68E-06	Down
LOC_Os06g04240.1	expressed protein	-1.876927245	0.00669995	Down
LOC_Os02g36070.1	cytochrome P450, putative, expressed	-1.872562052	0.00312697	Down
LOC_Os08g13740.1	expressed protein	-1.871687426	0.03874677	Down
LOC_Os05g35290.1	phenylalanine ammonia-lyase, putative, expressed	-1.867190419	0.03382199	Down
LOC_Os03g16610.1	laccase precursor protein, putative, expressed	-1.863163354	0.0029251	Down
LOC_Os01g17050.1	VQ domain containing protein, putative, expressed	-1.858079751	0.00013478	Down
LOC_Os02g53750.1	tyrosine protein kinase domain containing protein, putative, expressed	-1.854230229	0.01066685	Down
LOC_Os05g36290.1	actin, putative, expressed	-1.853046237	5.60E-06	Down
LOC_Os03g62830.1	nuclear antigen, putative, expressed	-1.820196317	7.31E-05	Down
LOC_Os04g52090.1	AP2 domain containing protein, expressed	-1.819936357	3.98E-13	Down
LOC_Os02g45780.1	zinc finger, C3HC4 type domain containing protein, expressed	-1.804477194	0.00261943	Down
LOC_Os05g47960.1	expressed protein	-1.804209491	0.0034901	Down
LOC_Os01g43480.1	AAA-type ATPase family protein, putative, expressed	-1.800688053	1.86E-13	Down
LOC_Os02g37300.1	heavy metal associated domain containing protein, expressed	-1.794192647	0.00693425	Down
LOC_Os04g02530.1	expressed protein	-1.786244349	5.32E-09	Down
LOC_Os05g36010.1	OsSub47 - Putative Subtilisin homologue, expressed	-1.784386241	0.02299971	Down
LOC_Os01g06580.1	fasciclin domain containing protein, expressed	-1.781584928	7.42E-08	Down
LOC_Os02g04130.1	DUF1645 domain containing protein, putative, expressed	-1.776756953	0.01559463	Down
LOC_Os12g22284.1	white-brown complex homolog protein 11, putative, expressed	-1.774211019	0.01338193	Down
LOC_Os10g36360.1	expressed protein	-1.769436899	0.00036075	Down
LOC_Os11g34720.1	Ser/Thr protein phosphatase family protein, putative, expressed	-1.760656114	0.0002798	Down
LOC_Os09g24580.1	EF hand family protein, putative, expressed	-1.751460865	0.01343147	Down
LOC_Os01g64110.1	glycosyl hydrolase, putative, expressed	-1.743449799	0.00045746	Down
LOC_Os06g38980.1	polyadenylate-binding protein, putative, expressed	-1.742566065	0.00464749	Down
LOC_Os02g45530.1	HOTHEAD precursor, putative, expressed	-1.734141367	1.21E-12	Down
LOC_Os03g55776.1	expressed protein	-1.731389027	3.08E-05	Down
LOC_Os04g40990.1	malate synthase, glyoxysomal, putative, expressed	-1.720439014	0.0192721	Down
LOC_Os11g03420.1	ZF-HD protein dimerisation region containing protein, expressed	-1.709579038	0.00252809	Down
LOC_Os04g47620.1	protein kinase APK1B, chloroplast precursor, putative, expressed	-1.709179897	0.04629073	Down
LOC_Os05g03760.1	zinc finger family protein, putative, expressed	-1.707715267	3.80E-13	Down
LOC_Os02g44235.1	expressed protein	-1.707044152	0.03826314	Down
LOC_Os05g28740.1	universal stress protein domain containing protein, putative, expressed	-1.704126669	0.01610346	Down

Supplementary table S2 continued..

Gene_ID	Putative Functions	log_2 fold change	P-value	Regulation
LOC_Os02g50570.1	retrotransposon protein, putative, unclassified, expressed	-1.693238353	6.85E-06	Down
LOC_Os05g40010.1	LTPL17 - Protease inhibitor/seed storage/LTP family protein precursor, expressed	-1.690071074	0.01347809	Down
LOC_Os03g38210.1	MYB family transcription factor, putative, expressed	-1.682431598	0.0392271	Down
LOC_Os10g02880.1	O-methyltransferase, putative, expressed	-1.675187072	3.64E-09	Down
LOC_Os02g44230.1	CPuORF22 - conserved peptide uORF-containing transcript, expressed	-1.660215296	1.08E-08	Down
LOC_Os10g22050.1	expressed protein	-1.644328752	0.00406125	Down
LOC_Os01g28790.1	PRAS-rich protein, putative, expressed	-1.637679152	6.80E-13	Down
LOC_Os04g46220.1	ethylene-responsive transcription factor, putative, expressed	-1.622654053	0.0012819	Down
LOC_Os11g21804.1	expressed protein	-1.618016136	0.01091928	Down
LOC_Os01g42190.1	heat shock protein DnaJ, putative, expressed	-1.617888634	1.22E-09	Down
LOC_Os03g47830.1	argonaute, putative, expressed	-1.617632696	0.04435422	Down
LOC_Os02g29130.1	ankyrin, putative, expressed	-1.617326284	0.00015726	Down
LOC_Os04g52260.1	LTPL124 - Protease inhibitor/seed storage/LTP family protein precursor, expressed	-1.612896244	0.04678095	Down
LOC_Os03g19070.1	long cell-linked locus protein, putative, expressed	-1.611075747	4.91E-09	Down
LOC_Os07g42924.1	dehydrogenase, putative, expressed	-1.60130714	0.00016088	Down
LOC_Os02g32580.1	expressed protein	-1.588540974	0.00229487	Down
LOC_Os02g34970.1	no apical meristem protein, putative, expressed	-1.588347717	1.30E-05	Down
LOC_Os10g37760.1	OsRhmbd17 - Putative Rhomboid homologue, expressed	-1.584315101	3.49E-09	Down
LOC_Os04g57340.1	AP2 domain containing protein, expressed	-1.584009283	0.00016425	Down
LOC_Os03g60080.1	NAC domain-containing protein 67, putative, expressed	-1.583399754	1.07E-09	Down
LOC_Os06g04230.1	expressed protein	-1.579468297	9.90E-07	Down
LOC_Os05g43970.1	heat- and acid-stable phosphoprotein, putative, expressed	-1.577386182	1.48E-07	Down
LOC_Os09g36220.1	response regulator receiver domain containing protein, expressed	-1.573691254	2.22E-18	Down
LOC_Os09g16330.2	pleiotropic drug resistance protein, putative, expressed	-1.570047609	0.00154865	Down
LOC_Os08g45150.1	GDSL-like lipase/acylhydrolase, putative, expressed	-1.569270464	2.71E-11	Down
LOC_Os01g01870.1	helix-loop-helix DNA-binding domain containing protein, expressed	-1.562683279	4.27E-10	Down
LOC_Os10g39860.1	expressed protein	-1.561607553	7.68E-22	Down
LOC_Os06g40770.1	expressed protein	-1.5564916	0.00874218	Down
LOC_Os03g18130.1	asparagine synthetase, putative, expressed	-1.555595343	3.33E-13	Down
LOC_Os01g73940.1	expressed protein	-1.548274252	0.00053486	Down
LOC_Os04g59540.1	phosphatidylinositol-4-phosphate 5-Kinase, putative, expressed	-1.547387486	0.03204527	Down
LOC_Os01g67810.1	transposon protein, putative, unclassified, expressed	-1.54290408	0.01796696	Down
LOC_Os02g52000.1	phosphate-induced protein 1 conserved region domain containing protein, expressed	-1.540419255	0.01081898	Down

Supplementary table S2 continued..

Gene_ID	Putative Functions	log_2 fold change	P-value	Regulation
LOC_Os01g49830.1	B3 DNA binding domain containing protein, expressed	-1.525781607	3.45E-06	Down
LOC_Os10g20470.1	MATE efflux family protein, putative, expressed	-1.523584917	0.04496708	Down
LOC_Os04g32920.1	potassium transporter, putative, expressed	-1.513582257	0.00062114	Down
LOC_Os09g16510.1	WRKY74, expressed	-1.500593275	0.00019065	Down
LOC_Os06g12500.1	membrane associated DUF588 domain containing protein, putative, expressed	-1.49819876	0.00260649	Down
LOC_Os01g70660.1	potassium transporter, putative, expressed	-1.492555956	0.01920862	Down
LOC_Os12g35490.1	expressed protein	-1.491769424	0.00652395	Down
LOC_Os05g31620.1	OsCML15 - Calmodulin-related calcium sensor protein, expressed	-1.490151528	3.53E-05	Down
LOC_Os05g04700.1	OsRCI2-6 - Hydrophobic protein LTI6B, expressed	-1.486743296	0.00026124	Down
LOC_Os03g02040.1	remorin, putative, expressed	-1.469687233	5.99E-07	Down
LOC_Os02g52990.1	OsSAUR12 - Auxin-responsive SAUR gene family member, expressed	-1.468014906	0.0028323	Down
LOC_Os01g05810.1	gamma-glutamyltranspeptidase 1 precursor, putative, expressed	-1.465260202	0.02024562	Down
LOC_Os01g03340.1	BBTI4 - Bowman-Birk type bran trypsin inhibitor precursor, expressed	-1.463498387	9.52E-12	Down
LOC_Os11g08100.1	eukaryotic aspartyl protease domain containing protein, expressed	-1.458059231	0.00254856	Down
LOC_Os11g10590.1	expressed protein	-1.440574803	1.91E-05	Down
LOC_Os01g03360.1	BBTI5 - Bowman-Birk type bran trypsin inhibitor precursor, expressed	-1.435669157	4.14E-06	Down
LOC_Os02g44880.1	expressed protein	-1.433956082	0.04672875	Down
LOC_Os09g26780.1	zinc-finger protein, putative, expressed	-1.431680353	5.46E-09	Down
LOC_Os01g04050.1	BBTI12 - Bowman-Birk type bran trypsin inhibitor precursor, expressed	-1.429128367	7.77E-07	Down
LOC_Os03g03700.1	MLO domain containing protein, putative, expressed	-1.422059872	0.01405631	Down
LOC_Os01g70110.1	No apical meristem protein, putative, expressed	-1.420199495	0.00021944	Down
LOC_Os08g38220.1	dof zinc finger domain containing protein, putative, expressed	-1.418966975	0.00011975	Down
LOC_Os04g45810.1	homeobox associated leucine zipper, putative, expressed	-1.417511533	0.0196313	Down
LOC_Os03g07200.1	expressed protein	-1.416264756	0.00757854	Down
LOC_Os07g42510.1	AP2 domain containing protein, expressed	-1.406980224	0.00020959	Down
LOC_Os09g11460.1	AP2 domain containing protein, expressed	-1.406617127	0.00764699	Down
LOC_Os05g07740.1	receptor-like protein kinase 2 precursor, putative, expressed	-1.396004373	0.00123778	Down
LOC_Os03g08330.1	ZIM domain containing protein, putative, expressed	-1.395717576	0.00115635	Down
LOC_Os05g46340.1	expressed protein	-1.39465983	0.03762598	Down
LOC_Os04g45960.1	OsSub42 - Putative Subtilisin homologue, expressed	-1.393132082	1.25E-18	Down
LOC_Os08g35160.2	heat shock protein DnaJ, putative, expressed	-1.39031122	0.01606642	Down
LOC_Os01g15270.1	expressed protein	-1.388995394	4.52E-10	Down
LOC_Os04g57200.1	heavy metal transport/detoxification protein, putative, expressed	-1.380335658	1.85E-05	Down

Supplementary table S2 continued..

Gene_ID	Putative Functions	log_2 fold change	P-value	Regulation
LOC_Os03g08970.1	expressed protein	-1.378501477	0.00017044	Down
LOC_Os08g03350.1	amino acid transporter, putative, expressed	-1.364551819	0.00252287	Down
LOC_Os12g36110.1	calmodulin binding protein, putative, expressed	-1.358002339	0.00011698	Down
LOC_Os05g43390.1	signal recognition particle 54 kDa protein, putative, expressed	-1.357398639	0.0335487	Down
LOC_Os03g18910.1	COBRA-like protein 7 precursor, putative, expressed	-1.353977163	3.16E-11	Down
LOC_Os05g41760.1	AP2 domain containing protein, expressed	-1.352087257	0.00067912	Down
LOC_Os05g08860.1	expressed protein	-1.350911493	0.04424883	Down
LOC_Os04g59190.1	peroxidase precursor, putative, expressed	-1.348744159	0.00075095	Down
LOC_Os12g25830.1	retrotransposon protein, putative, unclassified, expressed	-1.348117244	0.04669902	Down
LOC_Os01g07990.1	retrotransposon protein, putative, unclassified, expressed	-1.344835605	0.04643719	Down
LOC_Os01g45900.1	OsFBX20 - F-box domain containing protein, expressed	-1.339886803	0.04469769	Down
LOC_Os01g70790.1	SRC2 protein, putative, expressed	-1.337674404	2.74E-06	Down
LOC_Os03g01740.1	expressed protein	-1.326216489	0.00255311	Down
LOC_Os10g19960.1	beta-galactosidase, putative, expressed	-1.323102996	0.0054408	Down
LOC_Os04g38790.1	expressed protein	-1.319966873	3.93E-05	Down
LOC_Os05g48010.1	MYB family transcription factor, putative, expressed	-1.317444578	0.03381746	Down
LOC_Os01g47730.1	ras-related protein, putative, expressed	-1.3131306	5.80E-05	Down
LOC_Os03g03510.1	CAMK_KIN1/SNF1/Nim1_like.15 - CAMK includes calcium/calmodulin depedent prote	-1.306925855	9.83E-11	Down
LOC_Os03g15320.1	glyoxal oxidase-related, putative, expressed	-1.303336291	0.0008597	Down
LOC_Os03g55430.1	expressed protein	-1.302179716	0.01738829	Down
LOC_Os03g21380.2	OsCML27 - Calmodulin-related calcium sensor protein, expressed	-1.289828341	1.32E-10	Down
LOC_Os03g13840.2	senescence-associated protein, putative, expressed	-1.283046396	1.62E-07	Down
LOC_Os04g58810.1	CAF1 family ribonuclease containing protein, putative, expressed	-1.2800422	0.00157301	Down
LOC_Os11g29500.1	expressed protein	-1.277138877	0.03248871	Down
LOC_Os07g40020.1	GRAS family transcription factor domain containing protein, expressed	-1.273182779	0.04941224	Down
LOC_Os05g02010.1	expressed protein	-1.27191755	0.000342	Down
LOC_Os06g14450.1	exo70 exocyst complex subunit family protein, putative, expressed	-1.268158337	3.99E-06	Down
LOC_Os10g32980.1	CESA7 - cellulose synthase, expressed	-1.264451443	0.00430002	Down
LOC_Os04g58890.1	expressed protein	-1.263750724	0.00620006	Down
LOC_Os10g30790.2	inorganic phosphate transporter, putative, expressed	-1.254799319	0.02182834	Down
LOC_Os04g16742.1	NADPH-dependent oxidoreductase, putative, expressed	-1.25333896	0.00035308	Down
LOC_Os01g04330.1	OsCML16 - Calmodulin-related calcium sensor protein, expressed	-1.252629894	1.00E-05	Down
LOC_Os01g56530.1	DUF260 domain containing protein, putative, expressed	-1.245491728	0.00117669	Down

Supplementary table S2 continued..

Gene_ID	Putative Functions	log_2 fold change	P-value	Regulation
LOC_Os02g50130.1	hydrolase, NUDIX family, domain containing protein, expressed	-1.242113553	0.0003454	Down
LOC_Os04g58200.1	protochlorophyllide reductase A, chloroplast precursor, putative, expressed	-1.238807415	5.07E-05	Down
LOC_Os01g60640.1	WRKY21, expressed	-1.238457464	0.00342633	Down
LOC_Os01g14880.1	expressed protein	-1.233737937	4.25E-06	Down
LOC_Os07g07040.1	erythrocyte binding protein 3, putative, expressed	-1.229673613	0.00056424	Down
LOC_Os01g12440.1	AP2 domain containing protein, expressed	-1.227698846	0.00369268	Down
LOC_Os01g57730.1	peroxidase precursor, putative, expressed	-1.224873609	0.00531126	Down
LOC_Os02g48900.1	aspartic proteinase nepenthesin-1 precursor, putative, expressed	-1.222673967	0.00010782	Down
LOC_Os11g02440.1	chalcone--flavonone isomerase, putative, expressed	-1.222001588	0.00011911	Down
LOC_Os04g16854.1	NADPH-dependent oxidoreductase, putative, expressed	-1.218654911	0.00046647	Down
LOC_Os01g03690.1	TKL_IRAK_DUF26-lg.1 - DUF26 kinases have homology to DUF26 containing loci, expre	-1.216800309	0.0103354	Down
LOC_Os03g19020.1	PHD-finger family protein, expressed	-1.210757099	1.02E-08	Down
LOC_Os02g50600.1	glycosyl transferase 8 domain containing protein, putative, expressed	-1.209827624	5.31E-06	Down
LOC_Os09g33550.1	CCT/B-box zinc finger protein, putative, expressed	-1.209361977	0.00350229	Down
LOC_Os09g17740.1	chlorophyll A-B binding protein, putative, expressed	-1.206120597	7.23E-10	Down
LOC_Os04g49370.1	expressed protein	-1.205963062	0.00186056	Down
LOC_Os08g35190.1	auxin-repressed protein, putative, expressed	-1.202388091	0.02651691	Down
LOC_Os09g04160.1	expressed protein	-1.190396546	1.45E-05	Down
LOC_Os01g26000.1	expressed protein	-1.189624392	7.62E-07	Down
LOC_Os01g61990.2	ankyrin repeat-containing protein, putative, expressed	-1.189500452	0.00193688	Down
LOC_Os04g49000.1	zinc finger, C3HC4 type domain containing protein, expressed	-1.187633644	0.00139216	Down
LOC_Os12g16240.1	nmrA-like family domain containing protein, expressed	-1.18717668	0.03946118	Down
LOC_Os09g08720.1	cinnamoyl CoA reductase, putative, expressed	-1.179630516	0.00084214	Down
LOC_Os07g38230.1	expressed protein	-1.173428532	0.01879327	Down
LOC_Os01g74040.1	zinc finger, RING-type, putative, expressed	-1.171970527	0.02273557	Down
LOC_Os08g39450.1	AN1-like zinc finger domain containing protein, expressed	-1.168968746	1.56E-07	Down
LOC_Os11g10710.1	protein kinase domain containing protein, expressed	-1.163959985	0.03400334	Down
LOC_Os02g54010.1	lipase class 3 family protein, putative, expressed	-1.155642763	4.90E-07	Down
LOC_Os01g58150.1	expressed protein	-1.155301187	0.01676581	Down
LOC_Os02g52040.1	phosphate-induced protein 1 conserved region domain containing protein, expressed	-1.155278379	7.27E-10	Down
LOC_Os05g08640.1	transferase family protein, putative, expressed	-1.154903822	0.0051275	Down
LOC_Os06g50950.1	GDSL-like lipase/acylhydrolase, putative, expressed	-1.150017958	0.00171323	Down
LOC_Os02g03410.1	CAMK_CAMK_like.12 - CAMK includes calcium/calmodulin depedent protein kinases, [illegible]	-1.148291531	2.69E-08	Down

Supplementary table S2 continued..

Gene_ID	Putative Functions	log_2 fold change	P-value	Regulation
LOC_Os03g52680.1	expressed protein	-1.139560431	0.01496277	Down
LOC_Os03g16170.1	protein phosphatase 2C, putative, expressed	-1.139507297	0.00024359	Down
LOC_Os04g43680.1	MYB family transcription factor, putative, expressed	-1.138023195	3.60E-05	Down
LOC_Os04g49520.1	pentatricopeptide repeat protein PPR868-14, putative, expressed	-1.135507597	0.00018172	Down
LOC_Os03g03164.2	homeobox protein knotted-1, putative, expressed	-1.131568173	0.01264843	Down
LOC_Os01g48190.1	drought induced 19 protein, putative, expressed	-1.129591346	4.71E-10	Down
LOC_Os03g60820.1	transporter, major facilitator superfamily domain containing protein, expressed	-1.128738302	1.99E-13	Down
LOC_Os01g53880.1	OsIAA6 - Auxin-responsive Aux/IAA gene family member, expressed	-1.124478819	6.46E-05	Down
LOC_Os12g36880.1	pathogenesis-related Bet v I family protein, putative, expressed	-1.12399631	0.02027859	Down
LOC_Os02g51970.1	phosphate-induced protein 1 conserved region domain containing protein, expressed	-1.123632103	0.00405323	Down
LOC_Os04g20164.1	amine oxidase precursor, putative, expressed	-1.122788682	0.01613108	Down
LOC_Os11g10770.1	disease resistance protein RGA3, putative, expressed	-1.118140778	3.70E-05	Down
LOC_Os04g46400.1	AP2 domain containing protein, expressed	-1.117264635	0.03407811	Down
LOC_Os03g48310.1	plasma membrane ATPase, putative, expressed	-1.109367788	8.08E-05	Down
LOC_Os07g48229.1	vacuolar-sorting receptor precursor, putative, expressed	-1.101354485	0.00342965	Down
LOC_Os05g33840.1	transketolase, putative, expressed	-1.096269215	0.00068759	Down
LOC_Os06g11660.1	phosphate-induced protein 1 conserved region domain containing protein, expressed	-1.093103781	0.03024668	Down
LOC_Os04g30420.1	dehydrogenase, putative, expressed	-1.091860352	0.00052089	Down
LOC_Os09g38320.1	phytoene synthase, chloroplast precursor, putative, expressed	-1.088823489	0.00626508	Down
LOC_Os09g31200.1	AN1-like zinc finger domain containing protein, expressed	-1.088382542	7.70E-08	Down
LOC_Os04g33450.1	expressed protein	-1.081949036	0.00934308	Down
LOC_Os02g06930.1	protein kinase, putative, expressed	-1.080366937	2.30E-05	Down
LOC_Os02g44710.1	expressed protein	-1.078739114	8.17E-05	Down
LOC_Os01g04409.1	OsWAK1 - OsWAK receptor-like cytoplasmic kinase OsWAK-RLCK, expressed	-1.077281386	0.00188664	Down
LOC_Os05g41550.2	expressed protein	-1.067656148	1.55E-12	Down
LOC_Os02g01520.1	IQ calmodulin-binding motif family protein, putative, expressed	-1.065066794	9.42E-07	Down
LOC_Os10g39500.1	expressed protein	-1.064668409	0.00011498	Down
LOC_Os10g36703.1	CPuORF40 - conserved peptide uORF-containing transcript, expressed	-1.063505982	0.01598162	Down
LOC_Os05g44900.1	expressed protein	-1.058588728	0.03458904	Down
LOC_Os03g05460.1	expressed protein	-1.0554606	0.00029497	Down
LOC_Os03g45370.1	sodium/calcium exchanger protein, putative, expressed	-1.055439186	0.008023	Down
LOC_Os01g54600.1	WRKY13, expressed	-1.054220801	0.01113856	Down
LOC_Os01g09770.1	expressed protein	-1.053134298	0.00447124	Down

Supplementary table S2 continued..

Gene_ID	Putative Functions	log_2 fold change	P-value	Regulation
LOC_Os01g19130.1	protein phosphatase 2C, putative, expressed	-1.053134298	0.00052989	Down
LOC_Os01g49614.1	Protein kinase domain containing protein, expressed	-1.051962804	0.00222846	Down
LOC_Os01g63480.1	transferase family protein, putative, expressed	-1.051407553	0.01937676	Down
LOC_Os10g33920.1	transporter-related, putative, expressed	-1.051218368	0.02583472	Down
LOC_Os05g11910.1	GDSL-like lipase/acylhydrolase, putative, expressed	-1.049360047	1.19E-07	Down
LOC_Os06g43810.1	expressed protein	-1.046942464	0.00487442	Down
LOC_Os04g55260.1	thiamine-repressible mitochondrial transport protein THI74, putative, expressed	-1.04691289	4.73E-08	Down
LOC_Os02g45710.1	zinc finger, C3HC4 type domain containing protein, expressed	-1.044044727	0.00033243	Down
LOC_Os03g07250.1	cytochrome P450, putative, expressed	-1.041521127	9.99E-11	Down
LOC_Os06g02210.1	bark storage protein A precursor, putative, expressed	-1.03693707	0.01527026	Down
LOC_Os09g25490.1	CESA9 - cellulose synthase, expressed	-1.03575222	0.01653698	Down
LOC_Os12g29570.1	expressed protein	-1.030810437	0.00079408	Down
LOC_Os09g28340.1	expressed protein	-1.029887953	0.02995441	Down
LOC_Os03g15460.1	expressed protein	-1.029617604	0.00221577	Down
LOC_Os07g48500.1	stress responsive protein, putative, expressed	-1.026562765	0.03175087	Down
LOC_Os02g01190.1	POEI25 - Pollen Ole e I allergen and extensin family protein precursor, expressed	-1.023921653	2.42E-06	Down
LOC_Os12g06660.1	actin, putative, expressed	-1.021440323	0.00023054	Down
LOC_Os03g58850.1	uncharacterized PE-PGRS family protein PE_PGRS3 precursor, putative, expressed	-1.017433055	0.00906987	Down
LOC_Os06g30970.1	ubiquitin-conjugating enzyme, putative, expressed	-1.015560573	0.04280476	Down
LOC_Os03g38950.1	chloroplast post-illumination chlorophyll fluorescence increase protein, putative, expr	-1.015373493	0.00033444	Down
LOC_Os10g42410.3	zinc-binding protein, putative, expressed	-1.015166448	0.02136299	Down
LOC_Os01g01340.1	light-induced protein 1-like, putative, expressed	-1.011605115	0.0016031	Down
LOC_Os01g36950.1	N-rich protein, putative, expressed	-1.002745054	1.47E-09	Down
LOC_Os02g54820.1	trehalose-6-phosphate synthase, putative, expressed	-1.002526986	2.37E-06	Down
LOC_Os04g38840.1	LTPL81 - Protease inhibitor/seed storage/LTP family protein precursor, expressed	1.005218279	0.00127512	Up
LOC_Os06g03930.1	cytochrome P450 86A1, putative, expressed	1.012507319	1.8432E-06	Up
LOC_Os03g29864.1	retrotransposon protein, putative, unclassified, expressed	1.026765249	0.01258287	Up
LOC_Os03g48225.1	expressed protein	1.032878911	0.02097743	Up
LOC_Os08g41800.1	regulatory protein, putative, expressed	1.04254905	0.02997586	Up
LOC_Os01g19940.1	expressed protein	1.061505118	0.00483885	Up
LOC_Os06g43020.1	expressed protein	1.063074448	0.02808191	Up
LOC_Os04g47360.1	OsPOP9 - Putative Prolyl Oligopeptidase homologue, expressed	1.10171647	0.00826947	Up
LOC_Os02g24430.1	expressed protein	1.160318079	0.02652084	Up

Supplementary table S2 continued..

Gene_ID	Putative Functions	log_2 fold change	P-value	Regulation
LOC_Os01g11990.1	expressed protein	1.164096418	0.0461927	Up
LOC_Os04g14710.1	flavin-containing monooxygenase family protein, putative, expressed	1.164336054	2.3407E-08	Up
LOC_Os07g36370.1	OsFBX247 - F-box domain containing protein, expressed	1.179087883	0.01167463	Up
LOC_Os04g56995.1	expressed protein	1.181571385	1.7763E-05	Up
LOC_Os01g74020.1	MYB family transcription factor, putative, expressed	1.187390514	0.00017262	Up
LOC_Os03g38640.1	expressed protein	1.194507851	0.00113738	Up
LOC_Os06g02230.1	B3 DNA binding domain containing protein, expressed	1.220346895	0.0364827	Up
LOC_Os07g07420.1	gibberellin 20 oxidase 1-B, putative, expressed	1.221127363	0.03430207	Up
LOC_Os05g23950.1	TRAF-type zinc finger family protein, expressed	1.224937424	0.03612608	Up
LOC_Os02g02560.1	UTP--glucose-1-phosphate uridylyltransferase, putative, expressed	1.245750652	0.00129617	Up
LOC_Os02g02450.1	transposon protein, putative, unclassified, expressed	1.256083264	4.1171E-06	Up
LOC_Os06g37680.1	expressed protein	1.25896138	1.3074E-12	Up
LOC_Os03g07480.1	sucrose transporter, putativ, expressed	1.261147898	0.00611226	Up
LOC_Os10g22820.1	expressed protein	1.304356153	0.00171991	Up
LOC_Os05g14160.1	expressed protein	1.322732604	0.00772924	Up
LOC_Os10g12300.1	expressed protein	1.344071771	0.0025797	Up
LOC_Os07g13230.1	eukaryotic initiation factor 5A hypusine, DNA-binding OB fold family protein, expresse	1.368089001	0.00075918	Up
LOC_Os04g01320.1	serine/threonine-protein kinase receptor precursor, putative, expressed	1.432292529	0.04998089	Up
LOC_Os01g07660.1	expressed protein	1.463862608	0.03119322	Up
LOC_Os08g20730.1	peroxidase precursor, putative, expressed	1.471669657	4.5886E-18	Up
LOC_Os11g44600.1	calmodulin binding protein, putative, expressed	1.487252525	0.01179312	Up
LOC_Os08g13990.1	pentatricopeptide, putative, expressed	1.521679276	0.00135026	Up
LOC_Os05g49830.1	lipase class 3 family protein, putative, expressed	1.569796053	0.00050117	Up
LOC_Os12g35430.1	transposon protein, putative, unclassified, expressed	1.589438926	0.02419335	Up
LOC_Os05g06240.1	LRR receptor kinase, putative, expressed	1.664365474	0.01746339	Up
LOC_Os11g39350.1	expressed protein	1.676599727	0.04785837	Up
LOC_Os11g10640.1	protein kinase domain containing protein, expressed	1.695937083	0.00060812	Up
LOC_Os02g07900.1	ANTH/ENTH domain containing protein, putative, expressed	1.72296969	0.00022812	Up
LOC_Os06g19010.1	cadmium tolerance factor, putative, expressed	1.733259053	0.00959805	Up
LOC_Os05g38980.1	respiratory burst oxidase, putative, expressed	1.737270555	0.00135523	Up
LOC_Os07g01904.2	expressed protein	1.764718871	4.8455E-10	Up
LOC_Os03g45150.1	LTPL93 - Protease inhibitor/seed storage/LTP family protein precursor, expressed	1.822214244	2.0108E-06	Up
LOC_Os04g21340.1	expressed protein	1.899337332	0.00255429	Up

Supplementary table S2 continued..

Gene_ID	Putative Functions	log_2 fold change	P-value	Regulation
LOC_Os02g27540.1	NB-ARC domain containing protein, expressed	1.928464695	0.00040101	Up
LOC_Os02g27690.1	Leucine Rich Repeat family protein, expressed	1.957257308	0.00561928	Up
LOC_Os03g27110.1	hydrolase protein, putative, expressed	2.04784335	0.01182455	Up
LOC_Os03g07410.3	expressed protein	2.108215968	0.02312328	Up
LOC_Os02g18515.1	retrotransposon protein, putative, unclassified, expressed	2.141400171	3.5254E-06	Up
LOC_Os02g10300.1	polygalacturonase, putative, expressed	2.173269731	0.00631385	Up
LOC_Os10g41838.1	F-box protein interaction domain containing protein, expressed	2.202455296	0.0008377	Up
LOC_Os02g27592.1	expressed protein	2.22485045	0.01469903	Up
LOC_Os02g04030.1	phosphatidylinositol transfer, putative, expressed	2.235533299	0.01513359	Up
LOC_Os12g32390.1	expressed protein	2.251574726	0.00392679	Up
LOC_Os08g34300.1	retrotransposon protein, putative, unclassified, expressed	2.254294227	0.01084678	Up
LOC_Os07g03840.1	lectin-like receptor kinase, putative, expressed	2.279716824	0.00683005	Up
LOC_Os12g36410.1	transposon protein, putative, Pong sub-class, expressed	2.309242637	8.4395E-07	Up
LOC_Os01g18870.1	helix-loop-helix DNA-binding domain containing protein, expressed	2.337699231	7.124E-33	Up
LOC_Os10g42150.1	transposon protein, putative, unclassified, expressed	2.406297321	4.3936E-07	Up
LOC_Os09g33530.1	expressed protein	2.41343939	1.7021E-14	Up
LOC_Os04g48870.1	nitrilase-associated protein, putative, expressed	2.450018698	0.00751117	Up
LOC_Os01g09270.1	expressed protein	2.592710445	0.00034389	Up
LOC_Os01g08530.1	retrotransposon protein, putative, unclassified, expressed	2.594230271	0.02565799	Up
LOC_Os06g08310.1	plasma membrane ATPase, putative, expressed	2.605201389	0.02461482	Up
LOC_Os02g05365.1	expressed protein	2.628689742	0.0001966	Up
LOC_Os02g22760.1	retrotransposon protein, putative, LINE subclass, expressed	2.643626012	6.1209E-09	Up
LOC_Os05g51090.1	nodulin MtN3 family protein, putative, expressed	2.660561517	0.00027419	Up
LOC_Os05g01850.1	transposon protein, putative, unclassified, expressed	2.680220043	0.00098206	Up
LOC_Os02g06090.1	phytosulfokine receptor precursor, putative, expressed	2.692268538	0.00030711	Up
LOC_Os10g37170.1	retrotransposon protein, putative, unclassified, expressed	2.749950187	0.00537874	Up
LOC_Os10g07160.1	retrotransposon, putative, centromere-specific, expressed	2.765810842	6.6804E-10	Up
LOC_Os04g26230.1	pollen allergen, putative, expressed	2.797087274	0.02576078	Up
LOC_Os05g46690.1	expressed protein	2.825522617	4.2821E-16	Up
LOC_Os04g57350.1	EH domain-containing protein 1, putative, expressed	2.843582654	2.6266E-06	Up
LOC_Os02g52450.2	regulator of ribonuclease, putative, expressed	2.936812038	2.0321E-05	Up
LOC_Os02g34030.1	retrotransposon protein, putative, unclassified, expressed	2.942350221	0.02192933	Up
LOC_Os09g06910.1	retrotransposon protein, putative, unclassified, expressed	2.954459903	8.2791E-05	Up

Supplementary table S2 continued..

Gene_ID	Putative Functions	log_2 fold change	P-value	Regulation
LOC_Os04g39360.1	heavy metal transport/detoxification protein, putative, expressed	3.038565536	0.00261946	Up
LOC_Os04g26220.1	pollen allergen, putative, expressed	3.05522288	0.00191025	Up
LOC_Os02g01310.1	invertase/pectin methylesterase inhibitor family protein, putative, expressed	3.069262334	0.0341962	Up
LOC_Os08g34910.1	pectinesterase, putative, expressed	3.096341868	0.00048215	Up
LOC_Os01g06620.1	expressed protein	3.171832067	0.00306794	Up
LOC_Os11g08918.1	expressed protein	3.194793216	0.02939009	Up
LOC_Os09g12390.1	expressed protein	3.195877541	0.01292252	Up
LOC_Os10g42160.1	transposon protein, putative, unclassified, expressed	3.202366435	0.00154061	Up
LOC_Os05g07420.1	S-domain receptor-like protein kinase, putative, expressed	3.203310299	0.00012532	Up
LOC_Os07g05850.1	transposon protein, putative, CACTA, En/Spm sub-class, expressed	3.213652243	0.00697611	Up
LOC_Os08g38280.1	mucin-associated surface protein, putative, expressed	3.270235771	0.03064035	Up
LOC_Os05g33580.1	expressed protein	3.299382117	0.00824989	Up
LOC_Os09g30120.1	CSLE1 - cellulose synthase-like family E, expressed	3.321570333	7.2672E-16	Up
LOC_Os06g38510.2	pectate lyase precursor, putative, expressed	3.325955213	0.00037908	Up
LOC_Os03g19610.1	pectinesterase, putative, expressed	3.352858062	0.01674846	Up
LOC_Os11g01990.1	expressed protein	3.355671248	6.9596E-05	Up
LOC_Os05g03500.1	expressed protein	3.37855233	0.0006286	Up
LOC_Os03g04770.1	beta-amylase, putative, expressed	3.379330273	3.7142E-11	Up
LOC_Os01g10720.1	retrotransposon protein, putative, unclassified, expressed	3.420652614	0.01818127	Up
LOC_Os11g34710.1	Ser/Thr protein phosphatase family protein, putative, expressed	3.420652614	0.01818127	Up
LOC_Os10g19898.1	expressed protein	3.466078549	6.2782E-05	Up
LOC_Os02g06180.1	phytosulfokine receptor precursor, putative, expressed	3.480722162	0.00117298	Up
LOC_Os02g17292.1	retrotransposon protein, putative, unclassified, expressed	3.484864378	2.3064E-46	Up
LOC_Os07g05840.1	expressed protein	3.609722238	5.3388E-07	Up
LOC_Os09g39930.1	tyrosine protein kinase domain containing protein, putative, expressed	3.628689742	0.04555912	Up
LOC_Os01g06852.1	transposon protein, putative, unclassified, expressed	3.638958078	0.01793729	Up
LOC_Os11g41720.1	TNP1, putative, expressed	3.650716049	0.00194683	Up
LOC_Os12g44350.1	actin, putative, expressed	3.713725846	0.01354878	Up
LOC_Os06g48980.1	protein kinase APK1B, chloroplast precursor, putative, expressed	3.7688674	0.00059198	Up
LOC_Os01g74670.1	expressed protein	3.870965588	0.00016997	Up
LOC_Os01g12750.1	cytochrome P450, putative, expressed	3.891724148	0.00367932	Up
LOC_Os12g43700.1	SCP-like extracellular protein, expressed	3.908797661	0.0190391	Up
LOC_Os10g35930.1	OsPLIM2c - LIM domain protein, putative actin-binding protein and transcription facto	3.951946898	5.0374E-15	Up

Supplementary table S2 continued..

Gene_ID	Putative Functions	log_2 fold change	P-value	Regulation
LOC_Os08g44790.1	expansin precursor, putative, expressed	3.958838344	0.01592552	Up
LOC_Os05g38770.1	protein kinase APK1B, chloroplast precursor, putative, expressed	3.979186989	0.00427734	Up
LOC_Os06g21410.1	arabinogalactan peptide 23 precursor, putative, expressed	4.104268783	0.00114188	Up
LOC_Os12g08060.1	universal stress protein family protein, expressed	4.161184823	6.3148E-06	Up
LOC_Os02g17240.1	ATROPGEF7/ROPGEF7, putative, expressed	4.178886825	8.4111E-05	Up
LOC_Os03g42230.1	B3 DNA binding domain containing protein, expressed	4.234116345	0.00107885	Up
LOC_Os11g45220.1	IWS1 homolog A, putative, expressed	4.277014304	0.00186243	Up
LOC_Os02g29774.1	expressed protein	4.332296739	0.01897354	Up
LOC_Os05g24770.1	reticulon domain containing protein, putative, expressed	4.369771445	0.01667797	Up
LOC_Os06g40520.1	TNP1, putative, expressed	4.383735166	1.4422E-11	Up
LOC_Os12g24330.1	expressed protein	4.476686649	0.03204539	Up
LOC_Os02g58370.1	expressed protein	4.482918602	1.1843E-05	Up
LOC_Os08g13905.1	expressed protein	4.537826944	6.784E-06	Up
LOC_Os05g40060.1	WRKY48, expressed	4.543800845	0.02622939	Up
LOC_Os03g08350.1	ubiquitin carboxyl-terminal hydrolase, family 1, putative, expressed	4.638958078	6.7176E-06	Up
LOC_Os07g33860.1	60S ribosomal protein L44, putative, expressed	4.69907907	0.00038747	Up
LOC_Os01g70280.1	expressed protein	4.795864133	0.00073844	Up
LOC_Os01g15740.1	expressed protein	4.820550534	1.2596E-13	Up
LOC_Os01g48410.1	ATROPGEF7/ROPGEF7, putative, expressed	4.904548188	5.1054E-06	Up
LOC_Os01g07980.1	ankyrin, putative, expressed	4.91725924	0.00150878	Up
LOC_Os03g06110.1	retrotransposon protein, putative, unclassified, expressed	4.91725924	0.03338534	Up
LOC_Os09g06540.1	retrotransposon protein, putative, unclassified, expressed	4.956494403	1.2892E-05	Up
LOC_Os02g32240.1	retrotransposon protein, putative, unclassified, expressed	4.991259822	0.02696849	Up
LOC_Os09g36470.1	retrotransposon protein, putative, unclassified, expressed	4.997258562	1.5714E-11	Up
LOC_Os02g49340.1	nitrate-induced NOI protein, putative, expressed	5.084369226	0.00331576	Up
LOC_Os07g26480.2	P21-Rho-binding domain containing protein, putative, expressed	5.128763345	7.2884E-05	Up
LOC_Os09g26360.1	pectinesterase, putative, expressed	5.234116345	0.01220031	Up
LOC_Os02g32740.1	SNARE domain containing protein, putative, expressed	5.274193785	0.01054493	Up
LOC_Os01g66930.2	transposon protein, putative, CACTA, En/Spm sub-class, expressed	5.452439102	5.3453E-11	Up
LOC_Os01g59360.1	CAMK_CAMK_like.10 - CAMK includes calcium/calmodulin depedent protein kinases,	5.503908117	1.9302E-11	Up
LOC_Os11g14160.1	transposon protein, putative, Pong sub-class, expressed	5.506794471	2.0071E-33	Up
LOC_Os06g44470.1	pollen allergen, putative, expressed	5.527312722	0.00375614	Up
LOC_Os01g23880.1	expressed protein	5.553138709	9.5331E-09	Up

Supplementary table S2 continued..

Gene_ID	Putative Functions	log_2 fold change	P-value	Regulation
LOC_Os05g03310.1	expressed protein	5.560102657	0.00323637	Up
LOC_Os10g27480.1	expressed protein	5.625742458	8.1847E-17	Up
LOC_Os12g37660.1	pectinesterase, putative, expressed	5.654224834	0.00206616	Up
LOC_Os02g03500.1	expressed protein	5.669331727	0.04060119	Up
LOC_Os02g09450.1	glycerophosphoryl diester phosphodiesterase family protein, putative, expressed	5.720993847	3.5517E-06	Up
LOC_Os01g65240.1	transposon protein, putative, Pong sub-class, expressed	5.749705143	2.6503E-06	Up
LOC_Os09g01134.1	expressed protein	5.756794568	0.03220935	Up
LOC_Os12g18450.1	expressed protein	5.777856183	1.9766E-06	Up
LOC_Os06g43150.1	retrotransposon protein, putative, unclassified, expressed	5.780840042	2.2691E-10	Up
LOC_Os08g44660.1	EF hand family protein, putative, expressed	5.825836212	0.00083558	Up
LOC_Os10g08022.1	fructose-bisphospate aldolase isozyme, putative, expressed	5.825836212	0.00083558	Up
LOC_Os02g21320.1	expressed protein	5.848134879	2.5011E-05	Up
LOC_Os06g46560.1	myb-like DNA-binding domain containing protein, expressed	5.891724148	0.02184374	Up
LOC_Os11g12590.1	expressed protein	5.952677359	7.1329E-19	Up
LOC_Os02g36950.1	uncharacterized Cys-rich domain containing protein, putative, expressed	6.003232463	0.00028739	Up
LOC_Os03g43100.1	expressed protein	6.117351643	2.41E-68	Up
LOC_Os02g42810.1	oxidoreductase, short chain dehydrogenase/reductase family domain containing prot	6.117792227	0.00013328	Up
LOC_Os06g45160.1	pollen allergen, putative, expressed	6.128763345	0.00996896	Up
LOC_Os10g32810.1	beta-amylase, putative, expressed	6.15189322	1.3636E-26	Up
LOC_Os02g55800.1	expressed protein	6.213652243	0.00726635	Up
LOC_Os06g05260.1	pectate lyase precursor, putative, expressed	6.369771445	1.9189E-05	Up
LOC_Os01g64949.1	expressed protein	6.375923672	1.0696E-07	Up
LOC_Os12g42650.1	pollen preferential protein, putative, expressed	6.397252181	1.5186E-05	Up
LOC_Os05g51900.1	expressed protein	6.406297321	0.00327949	Up
LOC_Os01g39970.1	protein kinase domain containing protein, putative, expressed	6.446939305	5.2954E-17	Up
LOC_Os04g43870.1	transposon protein, putative, CACTA, En/Spm sub-class, expressed	6.490361586	4.8219E-13	Up
LOC_Os04g41250.1	armadillo/beta-catenin repeat family protein, putative, expressed	6.493760162	9.564E-11	Up
LOC_Os11g45730.1	pectinesterase, putative, expressed	6.502221741	5.9429E-06	Up
LOC_Os11g20330.2	expressed protein	6.560102657	0.00159449	Up
LOC_Os06g05209.1	pectate lyase precursor, putative, expressed	6.592163866	0.00135763	Up
LOC_Os06g03610.1	TKL_IRAK_CrRLK1L-1.13 - The CrRLK1L-1 subfamily has homology to the CrRLK1L hom	6.59612188	1.4448E-11	Up
LOC_Os07g30090.2	actin-depolymerizing factor, putative, expressed	6.638958078	0.00106628	Up
LOC_Os03g01640.1	expansin precursor, putative, expressed	6.713725846	0.00071228	Up

Supplementary table S2 continued..

Gene_ID	Putative Functions	log_2 fold change	P-value	Regulation
LOC_Os03g29600.1	transposon protein, putative, CACTA, En/Spm sub-class, expressed	6.713725846	0.00071228	Up
LOC_Os02g03490.1	major ampullate spidroin 3, putative, expressed	6.728225416	0.00065698	Up
LOC_Os09g26440.1	expressed protein	6.756794568	0.00055887	Up
LOC_Os09g02270.1	cyclase family protein, putative, expressed	6.770869753	0.00051542	Up
LOC_Os05g20150.1	TKL_IRAK_CrRLK1L-1.11 - The CrRLK1L-1 subfamily has homology to the CrRLK1L hom(	6.845920458	1.5866E-07	Up
LOC_Os11g45720.1	pectinesterase, putative, expressed	6.85695873	8.2447E-11	Up
LOC_Os10g30620.1	expressed protein	6.872271785	1.1562E-07	Up
LOC_Os12g10470.1	expressed protein	6.878785092	0.00026944	Up
LOC_Os03g05770.1	peroxidase precursor, putative, expressed	6.904548188	0.00022904	Up
LOC_Os12g36400.1	transposon protein, putative, Pong sub-class, expressed	6.97108194	1.0752E-21	Up
LOC_Os06g45230.1	pollen allergen, putative, expressed	7.003232463	0.00011945	Up
LOC_Os06g17450.1	expressed protein	7.044371158	6.0722E-16	Up
LOC_Os02g26290.1	fasciclin-like arabinogalactan protein 8 precursor, putative, expressed	7.128763345	4.8678E-05	Up
LOC_Os02g03510.1	fibroin heavy chain precursor, putative, expressed	7.182401309	3.2343E-05	Up
LOC_Os10g35160.1	expressed protein	7.198111392	1.3577E-09	Up
LOC_Os06g16050.1	transposon protein, putative, CACTA, En/Spm sub-class, expressed	7.213652243	1.2705E-85	Up
LOC_Os07g32710.1	retrotransposon protein, putative, unclassified, expressed	7.274193785	4.1185E-10	Up
LOC_Os01g50810.1	invertase/pectin methylesterase inhibitor family protein, putative, expressed	7.322773965	1.0273E-05	Up
LOC_Os02g48940.1	expressed protein	7.388149974	5.7837E-06	Up
LOC_Os01g63380.1	transposon protein, putative, unclassified, expressed	7.397252181	5.2059E-11	Up
LOC_Os10g40090.1	expansin precursor, putative, expressed	7.447773957	2.3176E-30	Up
LOC_Os10g17680.1	profilin domain containing protein, expressed	7.468073518	2.7613E-06	Up
LOC_Os05g40740.3	monocopper oxidase, putative, expressed	7.568185003	2.1634E-12	Up
LOC_Os01g43400.1	expressed protein	7.623528037	5.786E-07	Up
LOC_Os11g14150.1	transposon protein, putative, Pong sub-class, expressed	7.708511908	1.7914E-41	Up
LOC_Os07g49100.1	pectinesterase, putative, expressed	7.777856183	1.0265E-07	Up
LOC_Os01g43980.1	retrotransposon protein, putative, unclassified, expressed	7.784808944	9.4536E-08	Up
LOC_Os07g12260.1	retrotransposon protein, putative, unclassified, expressed	7.802045649	4.0803E-27	Up
LOC_Os10g17660.1	profilin domain containing protein, expressed	7.805468415	7.3837E-08	Up
LOC_Os11g31850.1	expressed protein	7.872271785	3.2398E-08	Up
LOC_Os05g30940.1	vacuolar calcium binding protein, putative, expressed	7.948555371	1.2057E-08	Up
LOC_Os04g33710.1	expressed protein	8.044371158	3.2287E-09	Up
LOC_Os03g42235.1	expressed protein	8.223920578	2.1383E-10	Up

Supplementary table S2 continued..

Gene_ID	Putative Functions	log_2 fold change	P-value	Regulation
LOC_Os03g18779.1	expressed protein	8.263211891	1.127E-101	Up
LOC_Os11g35300.1	expressed protein	8.313187916	4.8752E-11	Up
LOC_Os07g06834.1	expressed protein	8.496303851	6.8965E-91	Up
LOC_Os02g05670.1	expressed protein	8.527312722	6.8287E-24	Up
LOC_Os04g13000.1	retrotransposon protein, putative, LINE subclass, expressed	8.551974776	2.7164E-24	Up
LOC_Os03g50670.1	retrotransposon protein, putative, Ty3-gypsy subclass, expressed	8.866821476	7.1262E-43	Up
LOC_Os05g46530.1	invertase/pectin methylesterase inhibitor family protein, putative, expressed	8.985236034	2.8429E-46	Up
LOC_Os01g11140.1	retrotransposon protein, putative, unclassified, expressed	9.174481646	1.1672E-19	Up
LOC_Os06g15430.1	expressed protein	9.378989975	1.6148E-22	Up
LOC_Os10g42220.1	enoyl-CoA hydratase/isomerase family protein, putative, expressed	9.417524576	4.2337E-23	Up
LOC_Os03g63870.1	expressed protein	9.627401046	4.7602E-49	Up
LOC_Os10g42210.1	enoyl-CoA-hydratase, putative, expressed	9.800331216	1.0659E-29	Up
LOC_Os03g02470.1	expressed protein	10.56010266	7.1839E-86	Up
LOC_Os11g10090.1	transposon protein, putative, CACTA, En/Spm sub-class, expressed	11.5250654	4.851E-185	Up
LOC_Os04g32080.1	11-beta-hydroxysteroid dehydrogenase, putative, expressed	Inf	0.00724933	Up
LOC_Os04g36030.1	AIG1, putative, expressed	Inf	0.00030363	Up
LOC_Os03g56940.1	app1, putative, expressed	Inf	0.00357826	Up
LOC_Os01g27190.1	C2 domain containing protein, putative, expressed	Inf	2.3785E-11	Up
LOC_Os06g19110.1	cadmium tolerance factor, putative, expressed	Inf	0.00033155	Up
LOC_Os11g11730.1	cell wall adhesin, putative, expressed	Inf	1.5386E-05	Up
LOC_Os06g47110.1	COBRA-like protein precursor, putative, expressed	Inf	2.4587E-06	Up
LOC_Os09g16030.1	cytokinin-O-glucosyltransferase 1, putative, expressed	Inf	0.02975589	Up
LOC_Os04g11130.1	DEF9 - Defensin and Defensin-like DEFL family, expressed	Inf	0.00079945	Up
LOC_Os08g31080.1	DUF260 domain containing protein, putative, expressed	Inf	0.0462362	Up
LOC_Os04g54600.1	DUF617 domain containing protein, expressed	Inf	0.00663687	Up
LOC_Os08g07500.1	DUF617 domain containing protein, expressed	Inf	0.00017918	Up
LOC_Os01g62740.1	expressed protein	Inf	2.315E-20	Up
LOC_Os02g10530.1	expressed protein	Inf	0.02090814	Up
LOC_Os02g24205.1	expressed protein	Inf	0.00010579	Up
LOC_Os03g27830.1	expressed protein	Inf	3.8494E-23	Up
LOC_Os04g10500.1	expressed protein	Inf	0.00039532	Up
LOC_Os04g43090.1	expressed protein	Inf	0.00087306	Up
LOC_Os04g57260.1	expressed protein	Inf	0.0462362	Up

Supplementary table S2 continued..

Gene_ID	Putative Functions	log_2 fold change	P-value	Regulation
LOC_Os04g57270.1	expressed protein	Inf	6.4199E-06	Up
LOC_Os04g57280.1	expressed protein	Inf	1.3362E-06	Up
LOC_Os05g18274.1	expressed protein	Inf	3.9838E-10	Up
LOC_Os05g34490.1	expressed protein	Inf	0.03249918	Up
LOC_Os06g03390.1	expressed protein	Inf	4.7291E-12	Up
LOC_Os06g42650.1	expressed protein	Inf	4.031E-05	Up
LOC_Os06g46090.1	expressed protein	Inf	0.0146891	Up
LOC_Os06g48190.1	expressed protein	Inf	0.04233699	Up
LOC_Os07g08940.1	expressed protein	Inf	0.00113718	Up
LOC_Os07g15530.1	expressed protein	Inf	4.8033E-05	Up
LOC_Os08g12160.1	expressed protein	Inf	4.5414E-14	Up
LOC_Os08g13430.1	expressed protein	Inf	3.2057E-08	Up
LOC_Os09g33540.1	expressed protein	Inf	4.4002E-05	Up
LOC_Os11g08680.1	expressed protein	Inf	0.02975589	Up
LOC_Os11g08820.1	expressed protein	Inf	0.02724375	Up
LOC_Os11g11710.1	expressed protein	Inf	0.00230207	Up
LOC_Os11g16350.1	expressed protein	Inf	0.02494335	Up
LOC_Os11g31400.1	expressed protein	Inf	6.1066E-07	Up
LOC_Os11g36230.1	expressed protein	Inf	0.00251433	Up
LOC_Os11g36770.1	expressed protein	Inf	3.2057E-08	Up
LOC_Os12g23400.1	expressed protein	Inf	0.01344806	Up
LOC_Os01g25470.1	flavin monooxygenase, putative, expressed	Inf	4.703E-11	Up
LOC_Os04g11195.1	gamma-thionin family domain containing protein, expressed	Inf	0.00791834	Up
LOC_Os06g39060.1	glucan endo-1,3-beta-glucosidase precursor, putative, expressed	Inf	0.00043168	Up
LOC_Os10g21110.1	glycosyl hydrolase family 10 protein, putative, expressed	Inf	0.00030363	Up
LOC_Os04g42210.1	GPI-anchored protein, putative, expressed	Inf	8.3018E-08	Up
LOC_Os07g15340.1	hAT dimerisation domain-containing protein, putative, expressed	Inf	0.00036203	Up
LOC_Os01g14940.1	invertase/pectin methylesterase inhibitor family protein, putative, expressed	Inf	0.00047139	Up
LOC_Os01g20970.1	invertase/pectin methylesterase inhibitor family protein, putative, expressed	Inf	0.00039532	Up
LOC_Os05g20570.1	invertase/pectin methylesterase inhibitor family protein, putative, expressed	Inf	9.2541E-18	Up
LOC_Os01g60080.1	monocopper oxidase, putative, expressed	Inf	0.00192985	Up
LOC_Os12g08050.1	nodulation receptor kinase precursor, putative, expressed	Inf	0.0462362	Up
LOC_Os12g23170.1	Os12bglu38 - beta-glucosidase/beta-mannosidase/exoglucanase homologue, express	Inf	0.00015031	Up

Supplementary table S2 continued..

Gene_ID	Putative Functions	log_2 fold change	P-value	Regulation
LOC_Os02g39380.1	OsCML17 - Calmodulin-related calcium sensor protein, expressed	Inf	0.00135637	Up
LOC_Os04g47150.1	OsSub43 - Putative Subtilisin homologue, expressed	Inf	0.04233699	Up
LOC_Os08g23740.1	OsSub55 - Putative Subtilisin homologue, expressed	Inf	0.03249918	Up
LOC_Os08g04650.1	pectinesterase inhibitor domain containing protein, expressed	Inf	0.03876563	Up
LOC_Os04g38560.1	pectinesterase, putative, expressed	Inf	8.6869E-13	Up
LOC_Os04g54850.1	pectinesterase, putative, expressed	Inf	0.03549474	Up
LOC_Os02g50770.1	peroxidase precursor, putative, expressed	Inf	0.00556285	Up
LOC_Os06g36240.1	POEI21 - Pollen Ole e I allergen and extensin family protein precursor, expressed	Inf	3.383E-05	Up
LOC_Os01g13440.1	pollen allergen Cyn d 23, putative, expressed	Inf	4.5276E-06	Up
LOC_Os06g45180.1	pollen allergen, putative, expressed	Inf	0.00012609	Up
LOC_Os06g45190.1	pollen allergen, putative, expressed	Inf	0.03876563	Up
LOC_Os06g45290.1	pollen allergen, putative, expressed	Inf	4.4002E-05	Up
LOC_Os03g17030.1	polyadenylate-binding protein, putative, expressed	Inf	0.03549474	Up
LOC_Os01g33300.1	polygalacturonase, putative, expressed	Inf	0.00148135	Up
LOC_Os06g40890.1	polygalacturonase, putative, expressed	Inf	2.6827E-06	Up
LOC_Os07g13440.1	RALFL12 - Rapid ALkalinization Factor RALF family protein precursor, putative, express	Inf	0.00025467	Up
LOC_Os01g10470.1	RALFL17 - Rapid ALkalinization Factor RALF family protein precursor, expressed	Inf	0.02090814	Up
LOC_Os12g35690.1	RALFL5 - Rapid ALkalinization Factor RALF family protein precursor, expressed	Inf	0.00607617	Up
LOC_Os06g35590.1	reticuline oxidase-like protein precursor, putative, expressed	Inf	5.8723E-08	Up
LOC_Os01g69020.1	retrotransposon protein, putative, unclassified, expressed	Inf	7.2665E-07	Up
LOC_Os03g31560.1	retrotransposon protein, putative, unclassified, expressed	Inf	5.6822E-20	Up
LOC_Os07g14340.1	retrotransposon protein, putative, unclassified, expressed	Inf	8.3018E-08	Up
LOC_Os08g23210.1	retrotransposon protein, putative, unclassified, expressed	Inf	1.3362E-06	Up
LOC_Os09g06550.1	retrotransposon protein, putative, unclassified, expressed	Inf	0.02090814	Up
LOC_Os09g07940.1	retrotransposon protein, putative, unclassified, expressed	Inf	0.01604461	Up
LOC_Os11g43640.1	retrotransposon protein, putative, unclassified, expressed	Inf	0.02494335	Up
LOC_Os01g66860.2	serine/threonine protein kinase, putative, expressed	Inf	0.02090814	Up
LOC_Os10g26600.1	soluble inorganic pyrophosphatase, putative, expressed	Inf	4.3401E-10	Up
LOC_Os12g44280.1	subtilase, putative, expressed	Inf	0.04233699	Up
LOC_Os10g26470.1	sucrose transporter, putativ, expressed	Inf	0.00466273	Up
LOC_Os07g34130.1	TBC domain containing protein, expressed	Inf	8.6471E-07	Up
LOC_Os02g03520.1	THION25 - Plant thionin family protein precursor, expressed	Inf	0.02494335	Up
LOC_Os02g37220.1	transposon protein, putative, CACTA, En/Spm sub-class, expressed	Inf	3.6928E-05	Up

Supplementary table S2 continued..

Gene_ID	Putative Functions	log_2 fold change	P-value	Regulation
LOC_Os03g21100.1	transposon protein, putative, CACTA, En/Spm sub-class, expressed	Inf	0.00607617	Up
LOC_Os06g13270.1	transposon protein, putative, CACTA, En/Spm sub-class, expressed	Inf	0.00161786	Up
LOC_Os10g30670.1	transposon protein, putative, CACTA, En/Spm sub-class, expressed	Inf	9.9885E-75	Up
LOC_Os01g60590.1	transposon protein, putative, Pong sub-class, expressed	Inf	0.02975589	Up
LOC_Os10g31120.1	transposon protein, putative, Pong sub-class, expressed	Inf	0.0146891	Up
LOC_Os05g13830.1	TsetseEP precursor, putative, expressed	Inf	1.2917E-05	Up
LOC_Os01g57940.1	tyrosine protein kinase domain containing protein, putative, expressed	Inf	3.9838E-10	Up
LOC_Os06g12350.1	amino acid transporter, putative, expressed	Inf	0.01351186	Down
LOC_Os08g36920.1	AP2 domain containing protein, expressed	Inf	7.25E-05	Down
LOC_Os06g40818.1	aspartic proteinase, putative, expressed	Inf	0.0203469	Down
LOC_Os12g44210.1	ATPase, AAA family domain containing protein, expressed	Inf	0.0005126	Down
LOC_Os01g34920.1	beta-galactosidase precursor, putative, expressed	Inf	7.25E-05	Down
LOC_Os08g31870.1	cell division cycle protein 48, putative, expressed	Inf	0.00160894	Down
LOC_Os07g09130.1	CHCH domain containing protein, expressed	Inf	0.02822754	Down
LOC_Os04g41620.1	CHIT2 - Chitinase family protein precursor, expressed	Inf	0.0461064	Down
LOC_Os01g72270.1	cytochrome P450, putative, expressed	Inf	5.24E-05	Down
LOC_Os04g48200.1	cytochrome P450, putative, expressed	Inf	0.02600972	Down
LOC_Os10g37100.1	cytochrome P450, putative, expressed	Inf	1.48E-06	Down
LOC_Os08g16910.1	dehydrogenase, putative, expressed	Inf	0.00060353	Down
LOC_Os01g18490.1	expressed protein	Inf	2.14E-05	Down
LOC_Os01g22090.1	expressed protein	Inf	0.00646651	Down
LOC_Os01g24500.1	expressed protein	Inf	0.02208248	Down
LOC_Os04g28860.1	expressed protein	Inf	0.04248803	Down
LOC_Os06g06700.1	expressed protein	Inf	0.0239659	Down
LOC_Os07g09120.1	expressed protein	Inf	0.01244957	Down
LOC_Os09g22430.1	expressed protein	Inf	0.03607869	Down
LOC_Os11g07330.1	expressed protein	Inf	0.02208248	Down
LOC_Os11g14780.1	expressed protein	Inf	0.01874761	Down
LOC_Os11g14940.1	expressed protein	Inf	0.01874761	Down
LOC_Os12g43080.1	expressed protein	Inf	0.01351186	Down
LOC_Os11g47580.1	glycosyl hydrolase, putative, expressed	Inf	3.22E-05	Down
LOC_Os02g43330.1	homeobox associated leucine zipper, putative, expressed	Inf	0.00015084	Down
LOC_Os07g34520.1	isocitrate lyase, putative, expressed	Inf	8.59E-11	Down

Supplementary table S2 continued..

Gene_ID	Putative Functions	log_2 fold change	P-value	Regulation
LOC_Os03g14654.1	LTPL108 - Protease inhibitor/seed storage/LTP family protein precursor, expressed	Inf	0.0306341	Down
LOC_Os04g09520.1	LTPL59 - Protease inhibitor/seed storage/LTP family protein precursor, putative, expre	Inf	5.24E-05	Down
LOC_Os01g51450.1	NAP domain containing protein, putative, expressed	Inf	0.00015084	Down
LOC_Os10g09820.1	no apical meristem protein, putative, expressed	Inf	0.01466477	Down
LOC_Os10g34150.1	nodulin, putative, expressed	Inf	1.93E-08	Down
LOC_Os11g20160.1	O-methyltransferase, putative, expressed	Inf	0.0005126	Down
LOC_Os04g19810.1	OsFBL12 - F-box domain and LRR containing protein, expressed	Inf	0.03324538	Down
LOC_Os04g56250.1	OsFBX152 - F-box domain containing protein, expressed	Inf	0.0203469	Down
LOC_Os11g08640.1	retrotransposon protein, putative, unclassified, expressed	Inf	0.01351186	Down
LOC_Os01g41180.1	THION19 - Plant thionin family protein precursor, putative, expressed	Inf	0.0203469	Down
LOC_Os01g38680.1	transporter family protein, putative, expressed	Inf	4.07E-07	Down
LOC_Os10g34960.1	ubiquitin family protein, putative, expressed	Inf	0.00065488	Down
LOC_Os01g46270.1	wax synthase isoform 3, putative, expressed	Inf	0.00024598	Down
LOC_Os04g41060.1	zinc finger C-x8-C-x5-C-x3-H type family protein, expressed	Inf	5.85E-06	Down
LOC_Os08g36910.1	alpha-amylase precursor, putative, expressed	Inf	9.25E-05	Down

Supplementary table S3: Differentially expressed genes in *bHLH142* ^{OE} mature pollen anthers compared to wild type (log_2 fold change ≥ 1 and P value ≥ 0.05).

Gene ID	Putative functions	Log_2 fold change	P-Value	Regulation
LOC_Os02g55810.1	CXXXC9 - Cysteine-rich protein with paired CXXXC motifs precursor, expressed	-8.833929176	4.6331E-07	Down
LOC_Os08g26310.1	expressed protein	-6.465974465	0.01506696	Down
LOC_Os04g43410.1	Os4bglu18 - monolignol beta-glucoside homologue, expressed	-6.325230488	0.00115341	Down
LOC_Os09g24100.1	pollen allergen Cyn d 23, putative, expressed	-6.274548394	0.00023804	Down
LOC_Os12g02450.1	WRKY64, expressed	-6.032321287	0.03883352	Down
LOC_Os01g70870.1	ZOS1-23 - C2H2 zinc finger protein, expressed	-5.94875657	0.01820372	Down
LOC_Os05g30320.1	6-phospho-beta-galactosidase, putative, expressed	-5.923964109	0.00244604	Down
LOC_Os10g40710.1	expansin precursor, putative, expressed	-5.799958577	0.00036672	Down
LOC_Os09g38090.1	expressed protein	-5.10433666	0.00347365	Down
LOC_Os02g03870.1	periplasmic beta-glucosidase precursor, putative, expressed	-5.02211413	0.00147213	Down
LOC_Os02g41940.1	retrotransposon protein, putative, unclassified, expressed	-5.020348645	0.00952005	Down
LOC_Os04g09380.1	retrotransposon protein, putative, Ty1-copia subclass, expressed	-4.980547637	0.025059	Down
LOC_Os02g35320.1	expressed protein	-4.8642687	0.00783174	Down
LOC_Os06g23910.1	expressed protein	-4.862396286	0.02007456	Down
LOC_Os04g44060.1	aquaporin protein, putative, expressed	-4.799804648	0.00223675	Down
LOC_Os03g29310.1	transposon protein, putative, CACTA, En/Spm sub-class, expressed	-4.713067326	0.04326149	Down
LOC_Os04g51980.1	transferase family domain containing protein, expressed	-4.672425342	0.00766035	Down
LOC_Os05g38940.1	expressed protein	-4.617977558	0.03366925	Down
LOC_Os06g08680.1	expressed protein	-4.532247684	0.00299801	Down
LOC_Os07g33997.1	60S ribosomal protein L44, putative, expressed	-4.513435818	0.00368732	Down
LOC_Os03g01610.1	expansin precursor, putative, expressed	-4.415581095	0.0012766	Down
LOC_Os09g23999.1	pollen allergen Cyn d 23, putative, expressed	-4.40112332	0.01328362	Down
LOC_Os04g59370.1	thaumatin, putative, expressed	-4.396011906	0.02857619	Down
LOC_Os02g02080.1	expressed protein	-4.350497247	0.0159972	Down
LOC_Os11g03770.1	expressed protein	-4.326249701	0.0499195	Down
LOC_Os03g27590.1	OsSCP18 - Putative Serine Carboxypeptidase homologue, expressed	-4.26910892	0.00261694	Down
LOC_Os03g01630.1	expansin precursor, putative, expressed	-4.268726	0.0017281	Down
LOC_Os01g72740.1	cytochrome P450, putative, expressed	-4.224966365	0.02674058	Down

Supplementary table S3 continued...

Gene ID	Putative functions	Log_2 fold change	P-Value	Regulation
LOC_Os09g15460.1	retrotransposon protein, putative, Ty3-gypsy subclass, expressed	-4.209984973	0.0077736	Down
LOC_Os04g41340.1	4-nitrophenylphosphatase, putative, expressed	-4.18294315	0.02472592	Down
LOC_Os02g02090.1	peptidyl-prolyl cis-trans isomerase, putative, expressed	-4.12628209	0.01312741	Down
LOC_Os02g52850.1	receptor-like protein kinase like protein, putative, expressed	-4.111673395	0.00658713	Down
LOC_Os06g07360.1	expressed protein	-4.065095028	0.02038475	Down
LOC_Os04g11200.1	expressed protein	-4.057381502	0.00763424	Down
LOC_Os12g12970.1	RWP-RK domain-containing protein, putative, expressed	-4.003302394	0.01927917	Down
LOC_Os03g30220.1	mitochondrial import inner membrane translocase subunit Tim17, putative, expressec	-3.906547056	0.02572085	Down
LOC_Os10g02480.1	oxidoreductase, aldo/keto reductase family protein, putative, expressed	-3.886550147	0.02457422	Down
LOC_Os11g29500.1	expressed protein	-3.86490108	0.00658369	Down
LOC_Os11g27550.1	expressed protein	-3.849505787	0.01055061	Down
LOC_Os09g25810.2	nodulin, putative, expressed	-3.813624176	0.00442895	Down
LOC_Os09g24110.1	pollen allergen Cyn d 23, putative, expressed	-3.805949015	0.04276056	Down
LOC_Os05g16315.1	translation initiation factor IF-2, putative, expressed	-3.804732634	0.01396627	Down
LOC_Os11g29260.1	expressed protein	-3.77986003	0.00633091	Down
LOC_Os03g50310.1	CCT/B-box zinc finger protein, putative, expressed	-3.773029283	0.01824216	Down
LOC_Os08g38250.1	skin secretory protein xP2 precursor, putative, expressed	-3.748866895	0.00477917	Down
LOC_Os11g08909.1	expressed protein	-3.706611357	0.00575219	Down
LOC_Os03g27200.1	expressed protein	-3.701123658	0.00602593	Down
LOC_Os09g24159.1	pollen allergen Cyn d 23, putative, expressed	-3.673213485	0.00781944	Down
LOC_Os02g38170.1	expressed protein	-3.640003864	0.02746774	Down
LOC_Os01g12210.1	aluminum-activated malate transporter, putative, expressed	-3.634705676	0.00841305	Down
LOC_Os06g35320.1	polygalacturonase, putative, expressed	-3.627510926	0.00647368	Down
LOC_Os07g36060.1	SMP-30/Gluconolaconase/LRE-like region containing protein, expressed	-3.624096342	0.00849687	Down
LOC_Os02g49880.1	CCT/B-box zinc finger protein, putative, expressed	-3.61968388	0.04919326	Down
LOC_Os02g57000.1	C2 domain containing protein, putative, expressed	-3.61369932	0.00889067	Down
LOC_Os08g19790.1	retrotransposon protein, putative, Ty3-gypsy subclass, expressed	-3.587536444	0.01883557	Down
LOC_Os06g12890.1	expressed protein	-3.577788468	0.01664406	Down
LOC_Os02g33740.1	GPI-anchored protein, putative, expressed	-3.572889668	0.03215091	Down
LOC_Os06g42870.1	transposon protein, putative, Pong sub-class, expressed	-3.569614536	0.02136428	Down
LOC_Os08g32060.1	spotted leaf 11, putative, expressed	-3.557585538	0.01123159	Down
LOC_Os07g08290.1	expressed protein	-3.553437306	0.02176931	Down

Supplementary table S3 continued...

Gene ID	Putative functions	Log_2 fold change	P-Value	Regulation
LOC_Os01g23440.1	expressed protein	-3.541923318	0.01973954	Down
LOC_Os08g38280.1	mucin-associated surface protein, putative, expressed	-3.524863582	0.00732531	Down
LOC_Os08g07450.1	glutaredoxin protein, putative, expressed	-3.474944618	0.04319587	Down
LOC_Os07g31620.1	transposon protein, putative, Pong sub-class, expressed	-3.474071932	0.03886398	Down
LOC_Os02g10250.1	expressed protein	-3.473353995	0.01620873	Down
LOC_Os01g71800.1	lecithin cholesterol acyltransferase, putative, expressed	-3.472403916	0.01414881	Down
LOC_Os01g41450.1	UDP-glucoronosyl and UDP-glucosyl transferase domain containing protein, expressed	-3.470307815	0.01329054	Down
LOC_Os09g28790.1	retrotransposon protein, putative, unclassified, expressed	-3.466458997	0.01214126	Down
LOC_Os10g13680.1	retrotransposon protein, putative, unclassified, expressed	-3.461858356	0.0215932	Down
LOC_Os06g20480.1	expressed protein	-3.454329636	0.00880716	Down
LOC_Os01g54520.2	DUF1264 domain containing protein, putative, expressed	-3.422023003	0.01641534	Down
LOC_Os03g28300.1	protein kinase domain containing protein, expressed	-3.412151765	0.01184733	Down
LOC_Os08g01330.1	no apical meristem protein, putative, expressed	-3.411038789	0.01071241	Down
LOC_Os05g29740.1	invertase/pectin methylesterase inhibitor family protein, putative, expressed	-3.403937767	0.00936971	Down
LOC_Os09g26490.1	alpha/beta hydrolase fold, putative, expressed	-3.382507642	0.03663118	Down
LOC_Os04g58480.1	EF hand family protein, putative, expressed	-3.357256121	0.01056959	Down
LOC_Os03g59430.2	uncharacterized glycosyltransferase, putative, expressed	-3.337076731	0.01658141	Down
LOC_Os02g01300.1	invertase/pectin methylesterase inhibitor family protein, putative, expressed	-3.325413136	0.01165332	Down
LOC_Os07g39740.1	GDSL-like lipase/acylhydrolase, putative, expressed	-3.323530199	0.03954106	Down
LOC_Os03g02550.1	OsFBX76 - F-box domain containing protein, expressed	-3.320123598	0.02525515	Down
LOC_Os06g14170.1	expressed protein	-3.319565388	0.03918431	Down
LOC_Os02g56150.1	expressed protein	-3.311401936	0.01303976	Down
LOC_Os02g01340.1	ferredoxin--NADP reductase, chloroplast precursor, putative, expressed	-3.305136714	0.03281803	Down
LOC_Os03g44750.1	pollen preferential protein, putative, expressed	-3.298467294	0.01147573	Down
LOC_Os02g43280.1	aldehyde dehydrogenase, putative, expressed	-3.295856111	0.01577038	Down
LOC_Os11g29210.1	resistance-gene-interacting protein, putative, expressed	-3.295849851	0.02119614	Down
LOC_Os06g05260.1	pectate lyase precursor, putative, expressed	-3.290744825	0.01152871	Down
LOC_Os06g05730.1	expressed protein	-3.275963653	0.01210102	Down
LOC_Os08g13310.1	expressed protein	-3.274548394	0.01623796	Down
LOC_Os11g47970.1	AAA-type ATPase family protein, putative, expressed	-3.267471094	0.01643014	Down
LOC_Os05g10800.1	expressed protein	-3.255107793	0.01275891	Down
LOC_Os09g03850.1	expressed protein	-3.247313797	0.02846346	Down

Supplementary table S3 continued...

Gene ID	Putative functions	Log_2 fold change	P-Value	Regulation
LOC_Os09g12510.1	expressed protein	-3.244407982	0.01277001	Down
LOC_Os04g10500.1	expressed protein	-3.242642999	0.01265673	Down
LOC_Os03g13120.1	expressed protein	-3.241149851	0.02374317	Down
LOC_Os11g22760.1	hypothetical protein	-3.233683582	0.04260266	Down
LOC_Os02g20530.1	expressed protein	-3.231957632	0.02475895	Down
LOC_Os01g09730.1	expressed protein	-3.229834405	0.01305864	Down
LOC_Os01g10320.1	class III HD-Zip protein 8, putative, expressed	-3.227814727	0.02114079	Down
LOC_Os03g19700.1	plant protein of unknown function domain containing protein, expressed	-3.220232333	0.01989696	Down
LOC_Os02g10530.1	expressed protein	-3.209364255	0.01353703	Down
LOC_Os03g61090.1	expressed protein	-3.19923609	0.02506687	Down
LOC_Os08g39530.1	expressed protein	-3.196883981	0.01407915	Down
LOC_Os08g12660.1	retrotransposon protein, putative, Ty3-gypsy subclass, expressed	-3.191799501	0.03227438	Down
LOC_Os06g28510.1	retrotransposon protein, putative, unclassified, expressed	-3.187906363	0.0207989	Down
LOC_Os07g01770.1	expressed protein	-3.187577057	0.01401998	Down
LOC_Os05g29930.1	late embryogenesis abundant protein, putative, expressed	-3.183008862	0.01443369	Down
LOC_Os01g48540.1	glyoxal oxidase-related, putative, expressed	-3.181895262	0.01456537	Down
LOC_Os06g35524.1	expressed protein	-3.17991712	0.01538459	Down
LOC_Os10g02880.1	O-methyltransferase, putative, expressed	-3.178672713	0.01980632	Down
LOC_Os01g52100.1	DUF581 domain containing protein, expressed	-3.157455006	0.02484953	Down
LOC_Os02g33700.1	expressed protein	-3.15452828	0.02111504	Down
LOC_Os08g25830.1	expressed protein	-3.150772233	0.03251329	Down
LOC_Os03g20410.1	aquaporin SIP2-1, putative, expressed	-3.148948498	0.01537637	Down
LOC_Os06g05272.1	pectate lyase precursor, putative, expressed	-3.147475661	0.01557922	Down
LOC_Os01g71040.1	expressed protein	-3.138422036	0.0155565	Down
LOC_Os09g36060.1	endoglucanase, putative, expressed	-3.133574766	0.0318164	Down
LOC_Os07g45260.1	glycosyl transferase 8 domain containing protein, putative, expressed	-3.124674768	0.0159386	Down
LOC_Os12g36870.1	uncharacterized glycosyltransferase, putative, expressed	-3.101716825	0.01958538	Down
LOC_Os06g03390.1	expressed protein	-3.097005487	0.01659089	Down
LOC_Os07g34130.1	TBC domain containing protein, expressed	-3.095842785	0.01663717	Down
LOC_Os01g10890.1	CAMK_KIN1/SNF1/Nim1_like.8 - CAMK includes calcium/calmodulin depedent protein	-3.095591684	0.01664988	Down
LOC_Os11g13860.1	cyclin-dependent kinase, putative, expressed	-3.094603654	0.01670623	Down
LOC_Os05g38290.2	protein phosphatase 2C, putative, expressed	-3.086065042	0.01731778	Down

Supplementary table S3 continued...

Gene ID	Putative functions	Log_2 fold change	P-Value	Regulation
LOC_Os07g30960.1	monooxygenase, putative, expressed	-3.085658344	0.02949487	Down
LOC_Os11g03740.1	pectinesterase inhibitor domain containing protein, expressed	-3.085060351	0.01999965	Down
LOC_Os02g44080.1	aquaporin protein, putative, expressed	-3.082498487	0.02056691	Down
LOC_Os12g23200.1	photosystem I reaction center subunit XI, chloroplast precursor, putative, expressed	-3.080964204	0.04171535	Down
LOC_Os10g11750.1	LTPL89 - Protease inhibitor/seed storage/LTP family protein precursor, expressed	-3.080263493	0.01789454	Down
LOC_Os04g21340.1	expressed protein	-3.07304546	0.01735238	Down
LOC_Os01g08340.1	zinc finger family protein, putative, expressed	-3.072896728	0.0177445	Down
LOC_Os10g32590.1	universal stress protein domain containing protein, putative, expressed	-3.070655359	0.01783723	Down
LOC_Os06g04790.1	HAD superfamily phosphatase, putative, expressed	-3.065957351	0.01791597	Down
LOC_Os06g17120.1	UDP-glucoronosyl and UDP-glucosyl transferase domain containing protein, expressed	-3.063659817	0.02686777	Down
LOC_Os11g31400.1	expressed protein	-3.05865822	0.01785245	Down
LOC_Os09g35890.1	kinesin motor domain containing protein, expressed	-3.057082012	0.01793719	Down
LOC_Os11g18919.1	expressed protein	-3.054900633	0.02674466	Down
LOC_Os08g44680.1	photosystem I reaction center subunit II, chloroplast precursor, putative, expressed	-3.052057505	0.03095305	Down
LOC_Os04g33710.1	expressed protein	-3.051626509	0.01806831	Down
LOC_Os04g41250.1	armadillo/beta-catenin repeat family protein, putative, expressed	-3.051542637	0.01805402	Down
LOC_Os06g28194.1	expressed protein	-3.046902478	0.04085012	Down
LOC_Os11g30350.1	PB1 domain containing protein, expressed	-3.046460535	0.02072686	Down
LOC_Os01g40410.1	transmembrane amino acid transporter protein, putative, expressed	-3.044250774	0.02238698	Down
LOC_Os01g67910.1	expressed protein	-3.038553241	0.03098443	Down
LOC_Os02g51600.1	endonuclease/exonuclease/phosphatase family domain containing protein, expressed	-3.037961638	0.01884706	Down
LOC_Os05g40190.1	thioredoxin, putative, expressed	-3.035232882	0.04498351	Down
LOC_Os04g31924.1	nodulin, putative, expressed	-3.027693965	0.01927145	Down
LOC_Os07g42834.1	retrotransposon protein, putative, unclassified, expressed	-3.014954422	0.01976128	Down
LOC_Os05g51660.1	SCP-like extracellular protein, expressed	-3.012471688	0.01943845	Down
LOC_Os01g53430.1	anthocyanidin 5,3-O-glucosyltransferase, putative, expressed	-3.011609087	0.03345285	Down
LOC_Os10g29610.1	DUF623 domain containing protein, expressed	-3.009460329	0.03148537	Down
LOC_Os08g07500.1	DUF617 domain containing protein, expressed	-3.001239809	0.01986723	Down
LOC_Os02g46180.1	DUF581 domain containing protein, expressed	-2.993399794	0.02034484	Down
LOC_Os08g36560.1	emp24/gp25L/p24 family protein, putative, expressed	-2.992983449	0.02192054	Down
LOC_Os07g08000.1	expressed protein	-2.992015839	0.02080796	Down
LOC_Os06g44810.1	expressed protein	-2.99021742	0.02117149	Down

Supplementary table S3 continued...

Gene ID	Putative functions	Log_2 fold change	P-Value	Regulation
LOC_Os02g39380.1	OsCML17 - Calmodulin-related calcium sensor protein, expressed	-2.989828612	0.02029235	Down
LOC_Os05g30940.1	vacuolar calcium binding protein, putative, expressed	-2.987281136	0.02034443	Down
LOC_Os06g05209.1	pectate lyase precursor, putative, expressed	-2.98372548	0.02053907	Down
LOC_Os01g57940.1	tyrosine protein kinase domain containing protein, putative, expressed	-2.972335468	0.02090896	Down
LOC_Os05g51090.1	nodulin MtN3 family protein, putative, expressed	-2.971641994	0.02094262	Down
LOC_Os01g51060.1	hydrolase, putative, expressed	-2.961889168	0.02144259	Down
LOC_Os09g29050.1	mitochondrial carrier protein, putative, expressed	-2.960083535	0.02690147	Down
LOC_Os01g20970.1	invertase/pectin methylesterase inhibitor family protein, putative, expressed	-2.95777164	0.02153571	Down
LOC_Os10g10700.1	invertase/pectin methylesterase inhibitor family protein, putative, expressed	-2.949245109	0.02224319	Down
LOC_Os06g45150.1	pollen allergen, putative, expressed	-2.944682242	0.02215786	Down
LOC_Os04g21570.1	fasciclin-like arabinogalactan precursor protein, putative, expressed	-2.943849409	0.0252085	Down
LOC_Os09g28150.1	bifunctional monodehydroascorbate reductase and carbonic anhydrasenectarin-3 pre	-2.936565685	0.02319641	Down
LOC_Os02g32740.1	SNARE domain containing protein, putative, expressed	-2.933246601	0.02253505	Down
LOC_Os08g07600.1	expressed protein	-2.927713716	0.02560915	Down
LOC_Os06g10970.1	xyloglucan fucosyltransferase, putative, expressed	-2.927415353	0.02336718	Down
LOC_Os03g12520.1	STRUBBELIG-RECEPTOR FAMILY 7 precursor, putative, expressed	-2.92699983	0.02275697	Down
LOC_Os05g33580.1	expressed protein	-2.925559822	0.02281305	Down
LOC_Os03g13110.1	transposon protein, putative, Mariner sub-class, expressed	-2.921016011	0.0418558	Down
LOC_Os08g23130.3	oligopeptide transporter, putative, expressed	-2.920530104	0.02332536	Down
LOC_Os07g49350.1	expressed protein	-2.92017829	0.03434088	Down
LOC_Os02g56580.1	P21-Rho-binding domain containing protein, putative, expressed	-2.917388175	0.03405863	Down
LOC_Os04g40570.1	ABC transporter, ATP-binding protein, putative, expressed	-2.916964944	0.02432341	Down
LOC_Os07g06800.1	3-oxo-5-alpha-steroid 4-dehydrogenase, putative, expressed	-2.916249144	0.02924331	Down
LOC_Os03g61530.1	invertase/pectin methylesterase inhibitor family protein, putative, expressed	-2.916086562	0.02387968	Down
LOC_Os06g17450.1	expressed protein	-2.914477385	0.02322209	Down
LOC_Os03g41070.1	metal transporter Nramp2, putative, expressed	-2.909581116	0.02520744	Down
LOC_Os02g44590.1	OsSub20 - Putative Subtilisin homologue, expressed	-2.906076217	0.0239098	Down
LOC_Os08g44790.1	expansin precursor, putative, expressed	-2.904572503	0.02373895	Down
LOC_Os03g09160.1	hydroxyproline-rich glycoprotein family protein, putative, expressed	-2.902548196	0.02529619	Down
LOC_Os08g30800.1	KED, putative, expressed	-2.901604878	0.02429939	Down
LOC_Os07g26480.2	P21-Rho-binding domain containing protein, putative, expressed	-2.899451532	0.02390905	Down
LOC_Os03g41064.1	natural resistance-associated macrophage protein, putative, expressed	-2.897117546	0.02502999	Down

Supplementary table S3 continued...

Gene ID	Putative functions	Log_2 fold change	P-Value	Regulation
LOC_Os06g06990.1	expressed protein	-2.894521187	0.04483386	Down
LOC_Os07g35970.1	SMP-30/Gluconolaconase/LRE-like region containing protein, expressed	-2.891033957	0.02697659	Down
LOC_Os12g42650.1	pollen preferential protein, putative, expressed	-2.890844196	0.02428606	Down
LOC_Os01g09670.1	pollen-specific protein SF21, putative, expressed	-2.890565127	0.02696436	Down
LOC_Os11g46230.1	tetratricopeptide repeat domain containing protein, expressed	-2.88813705	0.02485581	Down
LOC_Os09g16560.1	hypothetical protein	-2.888078239	0.04502504	Down
LOC_Os02g47220.1	CPuORF20 - conserved peptide uORF-containing transcript, expressed	-2.883944935	0.02457671	Down
LOC_Os01g63620.1	expressed protein	-2.883647097	0.02469673	Down
LOC_Os01g12160.1	OsGH3.3 - Probable indole-3-acetic acid-amido synthetase, expressed	-2.883417258	0.03044477	Down
LOC_Os01g48130.2	no apical meristem protein, putative, expressed	-2.883298327	0.03410975	Down
LOC_Os11g29780.1	plant-specific domain TIGR01627 family protein, expressed	-2.88287815	0.03114125	Down
LOC_Os03g49590.1	expressed protein	-2.878572267	0.02787553	Down
LOC_Os03g51010.1	hydrolase, alpha/beta fold family domain containing protein, expressed	-2.87762022	0.02487853	Down
LOC_Os01g23370.1	expressed protein	-2.876466737	0.02735913	Down
LOC_Os06g09860.1	expressed protein	-2.870657464	0.02533076	Down
LOC_Os05g50830.1	protein kinase family protein, putative, expressed	-2.868634693	0.02662014	Down
LOC_Os04g50216.1	SNARE associated Golgi protein, putative, expressed	-2.865908609	0.02556233	Down
LOC_Os01g55520.1	ATROPGEF7/ROPGEF7, putative, expressed	-2.864762362	0.02551969	Down
LOC_Os12g19381.1	ribulose bisphosphate carboxylase small chain, chloroplast precursor, putative, expres	-2.863389719	0.02904382	Down
LOC_Os10g40950.1	polyol transporter 5, putative, expressed	-2.86079057	0.02854683	Down
LOC_Os04g01150.1	ELMO/CED-12 family protein, putative, expressed	-2.853063132	0.02607915	Down
LOC_Os01g21580.1	esterase/lipase/thioesterase family protein, putative, expressed	-2.851868964	0.03018975	Down
LOC_Os09g23980.1	retrotransposon protein, putative, unclassified, expressed	-2.851550858	0.02706157	Down
LOC_Os10g27170.1	calmodulin-binding protein, putative, expressed	-2.849746609	0.02666216	Down
LOC_Os02g02450.1	transposon protein, putative, unclassified, expressed	-2.8496226	0.02610938	Down
LOC_Os06g09260.1	expressed protein	-2.847103873	0.03344174	Down
LOC_Os11g36230.1	expressed protein	-2.846448378	0.02638413	Down
LOC_Os06g10210.1	expressed protein	-2.846173629	0.02631706	Down
LOC_Os05g48640.1	ATROPGEF7/ROPGEF7, putative, expressed	-2.845261939	0.02761265	Down
LOC_Os08g39300.1	aminotransferase, putative, expressed	-2.843226031	0.03877675	Down
LOC_Os12g22630.1	retrotransposon protein, putative, unclassified, expressed	-2.842922921	0.02931017	Down
LOC_Os12g07700.1	nifU, putative, expressed	-2.839546425	0.02666899	Down

Supplementary table S3 continued...

Gene ID	Putative functions	Log_2 fold change	P-Value	Regulation
LOC_Os03g01640.1	expansin precursor, putative, expressed	-2.839292793	0.02668123	Down
LOC_Os01g59360.1	CAMK_CAMK_like.10 - CAMK includes calcium/calmodulin depedent protein kinases, e	-2.835405535	0.02680744	Down
LOC_Os01g44430.1	retrotransposon protein, putative, unclassified, expressed	-2.831559721	0.02719383	Down
LOC_Os07g33850.2	ras-related protein, putative, expressed	-2.828100125	0.0280644	Down
LOC_Os02g10440.1	protein with a conserved N-terminal region, putative, expressed	-2.827372936	0.02736986	Down
LOC_Os05g32960.1	expressed protein	-2.824000963	0.02752236	Down
LOC_Os02g24430.1	expressed protein	-2.822075459	0.02747269	Down
LOC_Os12g02640.1	cytochrome P450 72A1, putative, expressed	-2.815575429	0.04688054	Down
LOC_Os09g36280.1	glycosyl hydrolases family 17, putative, expressed	-2.808061751	0.02932749	Down
LOC_Os04g40150.1	glycosyl transferase family 17 protein, putative, expressed	-2.807915706	0.02828452	Down
LOC_Os01g05100.1	expressed protein	-2.805467256	0.0286113	Down
LOC_Os11g08930.1	expressed protein	-2.803817527	0.02869223	Down
LOC_Os01g10470.1	RALFL17 - Rapid ALkalinization Factor RALF family protein precursor, expressed	-2.799542496	0.02875733	Down
LOC_Os10g07970.1	anthocyanidin 5,3-O-glucosyltransferase, putative, expressed	-2.793343765	0.0323156	Down
LOC_Os09g16970.1	expressed protein	-2.791297842	0.03874952	Down
LOC_Os01g27190.1	C2 domain containing protein, putative, expressed	-2.789347268	0.02912761	Down
LOC_Os11g42440.1	expressed protein	-2.786766203	0.03178302	Down
LOC_Os01g13270.1	AGC_PVPK_like_kin82y.4 - ACG kinases include homologs to PKA, PKG and PKC, expres	-2.786704286	0.02995456	Down
LOC_Os02g07900.1	ANTH/ENTH domain containing protein, putative, expressed	-2.784908762	0.02934296	Down
LOC_Os09g12630.1	expressed protein	-2.78320316	0.03049842	Down
LOC_Os11g25560.1	BHLH transcription factor, putative, expressed	-2.781601315	0.03149299	Down
LOC_Os08g35440.1	Ser/Thr protein phosphatase family protein, putative, expressed	-2.775837018	0.03054915	Down
LOC_Os03g57500.1	zinc finger, C3HC4 type domain containing protein, expressed	-2.774803179	0.03004821	Down
LOC_Os10g35930.1	OsPLIM2c - LIM domain protein, putative actin-binding protein and transcription facto	-2.771331908	0.03005262	Down
LOC_Os01g70120.1	expressed protein	-2.768627799	0.03113643	Down
LOC_Os09g36520.1	ki1 protein, putative, expressed	-2.765834777	0.03305851	Down
LOC_Os04g52950.1	nitrate-induced NOI protein, expressed	-2.761826414	0.03089672	Down
LOC_Os06g35370.1	polygalacturonase, putative, expressed	-2.755123446	0.03139421	Down
LOC_Os09g28130.1	carbonic anhydrase family protein, putative, expressed	-2.753621425	0.04247427	Down
LOC_Os12g03510.1	PME/invertase inhibitor, putative, expressed	-2.752610892	0.03300758	Down
LOC_Os12g37660.1	pectinesterase, putative, expressed	-2.750176126	0.03129175	Down
LOC_Os01g55850.1	expressed protein	-2.749593202	0.0407932	Down

Supplementary table S3 continued...

Gene ID	Putative functions	Log_2 fold change	P-Value	Regulation
LOC_Os02g12300.1	pectate lyase precursor, putative, expressed	-2.749170016	0.03141712	Down
LOC_Os01g13370.1	seven in absentia protein family domain containing protein, expressed	-2.745667164	0.03155086	Down
LOC_Os02g26320.1	fasciclin-like arabinogalactan precursor protein, putative, expressed	-2.743828484	0.03182876	Down
LOC_Os09g15790.1	ras-related protein, putative, expressed	-2.741490728	0.03221433	Down
LOC_Os06g40890.1	polygalacturonase, putative, expressed	-2.739956505	0.03182862	Down
LOC_Os11g02580.1	esterase, putative, expressed	-2.738528435	0.03254108	Down
LOC_Os03g24160.1	phosphatidylinositol-4-phosphate 5-kinase, putative, expressed	-2.737543066	0.03244213	Down
LOC_Os04g32740.1	hydrolase, NUDIX family, domain containing protein, expressed	-2.735960338	0.03407098	Down
LOC_Os02g54100.1	expressed protein	-2.735256127	0.03380142	Down
LOC_Os01g16950.1	zinc finger, C3HC4 type domain containing protein, expressed	-2.734764431	0.03234373	Down
LOC_Os10g27480.1	expressed protein	-2.733882717	0.03212704	Down
LOC_Os02g55910.1	monogalactosyldiacylglycerol synthase, putative, expressed	-2.73313331	0.03237874	Down
LOC_Os06g10480.1	expressed protein	-2.731843296	0.03235935	Down
LOC_Os09g28450.1	paramyosin, putative, expressed	-2.729688111	0.03278008	Down
LOC_Os06g03830.1	retinol dehydrogenase, putative, expressed	-2.727410454	0.03443282	Down
LOC_Os01g23880.1	expressed protein	-2.727120024	0.03253121	Down
LOC_Os06g12560.1	zinc finger, C3HC4 type, domain containing protein, expressed	-2.726030437	0.03680403	Down
LOC_Os03g14610.1	HVA22, putative, expressed	-2.724702851	0.03271309	Down
LOC_Os10g11980.1	transferase family protein, putative, expressed	-2.723132304	0.03450735	Down
LOC_Os04g26834.1	eukaryotic aspartyl protease domain containing protein, expressed	-2.72296037	0.03297485	Down
LOC_Os03g64310.1	expressed protein	-2.722670778	0.04124467	Down
LOC_Os03g31210.1	UDP-glucose 6-dehydrogenase, putative, expressed	-2.719887744	0.04537225	Down
LOC_Os01g48410.1	ATROPGEF7/ROPGEF7, putative, expressed	-2.717028757	0.03313227	Down
LOC_Os06g27850.1	peroxidase precursor, putative, expressed	-2.716351038	0.03324285	Down
LOC_Os01g51270.1	THION44 - Plant thionin family protein precursor, putative, expressed	-2.715971386	0.03445236	Down
LOC_Os05g11790.1	CAMK_KIN1/SNF1/Nim1_like.19 - CAMK includes calcium/calmodulin depedent protei	-2.715943607	0.03349397	Down
LOC_Os02g16000.1	E1-BTB1 - Bric-a-Brac, Tramtrack, and Broad Complex domain with E1 subfamily conse	-2.713455606	0.0334277	Down
LOC_Os08g42390.1	glycerophosphoryl diester phosphodiesterase family protein, putative, expressed	-2.711949175	0.03546068	Down
LOC_Os05g20150.1	TKL_IRAK_CrRLK1L-1.11 - The CrRLK1L-1 subfamily has homology to the CrRLK1L hom	-2.709699876	0.03355876	Down
LOC_Os11g08860.1	expressed protein	-2.70828313	0.03409086	Down
LOC_Os02g42280.1	UDP-glucoronosyl/UDP-glucosyl transferase family protein, putative, expressed	-2.706692172	0.03996578	Down
LOC_Os05g38770.1	protein kinase APK1B, chloroplast precursor, putative, expressed	-2.706070009	0.03382442	Down

Supplementary table S3 continued...

Gene ID	Putative functions	Log_2 fold change	P-Value	Regulation
LOC_Os03g27110.1	hydrolase protein, putative, expressed	-2.704748372	0.03385852	Down
LOC_Os05g36260.1	soluble inorganic pyrophosphatase, putative, expressed	-2.704388508	0.03412095	Down
LOC_Os03g56060.1	CSLC9 - cellulose synthase-like family C, expressed	-2.702941156	0.03402401	Down
LOC_Os08g42640.1	zinc finger, C3HC4 type domain containing protein, expressed	-2.702565788	0.03420132	Down
LOC_Os02g58610.1	protein kinase, putative, expressed	-2.702498725	0.03692081	Down
LOC_Os11g45720.1	pectinesterase, putative, expressed	-2.702421284	0.03397546	Down
LOC_Os08g04650.1	pectinesterase inhibitor domain containing protein, expressed	-2.701103614	0.03421241	Down
LOC_Os10g37260.1	fringe-related protein, putative, expressed	-2.695528411	0.03592095	Down
LOC_Os07g29770.1	zinc finger protein, putative, expressed	-2.692740744	0.03592507	Down
LOC_Os05g21180.2	phosphatidic acid phosphatase-related, putative, expressed	-2.692176708	0.03472784	Down
LOC_Os09g01134.1	expressed protein	-2.691833729	0.03488889	Down
LOC_Os11g44880.1	kinesin-4, putative, expressed	-2.691630942	0.03492756	Down
LOC_Os11g04170.1	CAMK_CAMK_like.42 - CAMK includes calcium/calmodulin depedent protein kinases, e	-2.690141677	0.03522116	Down
LOC_Os12g03970.1	CAMK_CAMK_like.44 - CAMK includes calcium/calmodulin depedent protein kinases, e	-2.688204107	0.03543703	Down
LOC_Os08g39460.1	AGC_PVPK_like_CDK8.2 - ACG kinases include homologs to PKA, PKG and PKC, express	-2.687990211	0.03502196	Down
LOC_Os03g01020.1	pectinesterase inhibitor domain containing protein, putative, expressed	-2.686207276	0.03526395	Down
LOC_Os04g37520.1	extracellular ligand-gated ion channel, putative, expressed	-2.683398378	0.03550716	Down
LOC_Os10g11860.1	MATE efflux family protein, putative, expressed	-2.683221988	0.03732383	Down
LOC_Os05g05650.1	C2 domain containing protein, putative, expressed	-2.680989631	0.03625063	Down
LOC_Os05g50380.1	glucose-1-phosphate adenylyltransferase large subunit, chloroplast precursor, putative	-2.679884012	0.03556687	Down
LOC_Os02g25900.1	pollen signalling protein with adenylyl cyclase activity, putative, expressed	-2.67687366	0.0355464	Down
LOC_Os04g54600.1	DUF617 domain containing protein, expressed	-2.676373839	0.03574244	Down
LOC_Os09g03890.1	octicosapeptide/Phox/Bem1p, putative, expressed	-2.672440427	0.03597793	Down
LOC_Os02g06510.1	myosin heavy chain, putative, expressed	-2.67230059	0.04083825	Down
LOC_Os01g65680.1	4,5-DOPA dioxygenase extradiol, putative, expressed	-2.672149202	0.03950415	Down
LOC_Os12g37480.1	invertase/pectin methylesterase inhibitor family protein, putative, expressed	-2.671561306	0.03733389	Down
LOC_Os11g23220.1	myosin, putative, expressed	-2.670858371	0.03686995	Down
LOC_Os07g13580.1	glucan endo-1,3-beta-glucosidase precursor, putative, expressed	-2.670295436	0.03623573	Down
LOC_Os05g09380.2	AR791, putative, expressed	-2.66789863	0.03624835	Down
LOC_Os01g06620.1	expressed protein	-2.667803341	0.03617168	Down
LOC_Os06g25800.1	retrotransposon protein, putative, unclassified, expressed	-2.667448349	0.03758481	Down
LOC_Os01g62950.1	ras-related protein, putative, expressed	-2.667196327	0.03643124	Down

Supplementary table S3 continued...

Gene ID	Putative functions	Log_2 fold change	P-Value	Regulation
LOC_Os12g02510.1	esterase, putative, expressed	-2.665985267	0.03687647	Down
LOC_Os12g16180.1	lipase, putative, expressed	-2.662229429	0.03725915	Down
LOC_Os02g43840.1	ethylene-responsive element-binding protein, putative, expressed	-2.661228034	0.03756948	Down
LOC_Os07g13450.1	RALFL13 - Rapid ALkalinization Factor RALF family protein precursor, expressed	-2.661220751	0.03812886	Down
LOC_Os08g41650.1	expressed protein	-2.661203149	0.03869311	Down
LOC_Os12g12860.1	CAMK_CAMK_like.46 - CAMK includes calcium/calmodulin depedent protein kinases, e	-2.661042515	0.03659749	Down
LOC_Os06g03610.1	TKL_IRAK_CrRLK1L-1.13 - The CrRLK1L-1 subfamily has homology to the CrRLK1L homo	-2.660538937	0.03657275	Down
LOC_Os10g32900.1	CCT motif family protein, expressed	-2.66032573	0.03716236	Down
LOC_Os11g45220.1	IWS1 homolog A, putative, expressed	-2.657351413	0.03685788	Down
LOC_Os01g33300.1	polygalacturonase, putative, expressed	-2.655246829	0.03708023	Down
LOC_Os02g36924.1	OsMADS27 - MADS-box family gene with MIKCc type-box, expressed	-2.651076022	0.04285776	Down
LOC_Os06g47110.1	COBRA-like protein precursor, putative, expressed	-2.647958247	0.03743206	Down
LOC_Os10g08022.1	fructose-bisphospate aldolase isozyme, putative, expressed	-2.646373677	0.03754915	Down
LOC_Os05g11810.1	gibberellin 2-beta-dioxygenase 1, putative, expressed	-2.645552827	0.0395602	Down
LOC_Os08g35770.1	expressed protein	-2.644926063	0.04795358	Down
LOC_Os02g05670.1	expressed protein	-2.643994863	0.03762703	Down
LOC_Os05g49580.1	plastocyanin-like domain containing protein, putative, expressed	-2.64383292	0.03965191	Down
LOC_Os03g58190.1	expressed protein	-2.639228514	0.03871795	Down
LOC_Os12g16200.1	glutathione synthetase, chloroplast precursor, putative, expressed	-2.638842741	0.0471911	Down
LOC_Os01g69090.1	CBS domain-containing protein, putative, expressed	-2.636847672	0.03843277	Down
LOC_Os02g01290.1	expressed protein	-2.632048524	0.03983534	Down
LOC_Os05g38890.1	transposon protein, putative, CACTA, En/Spm sub-class, expressed	-2.631815435	0.04661008	Down
LOC_Os02g55800.1	expressed protein	-2.628770606	0.04013213	Down
LOC_Os04g42350.1	heavy metal-associated domain containing protein, expressed	-2.628735069	0.04493891	Down
LOC_Os04g49650.1	DUF581 domain containing protein, expressed	-2.627486813	0.03884422	Down
LOC_Os04g55510.1	zinc finger, C3HC4 type domain containing protein, expressed	-2.626359427	0.03894776	Down
LOC_Os04g55260.1	thiamine-repressible mitochondrial transport protein THI74, putative, expressed	-2.625963862	0.03956294	Down
LOC_Os09g32870.1	OsFBX336 - F-box domain containing protein, expressed	-2.623390426	0.04982874	Down
LOC_Os01g43910.1	CGMC_MAPKCMGC_2.4 - CGMC includes CDA, MAPK, GSK3, and CLKC kinases, expres	-2.62175352	0.0392966	Down
LOC_Os04g55110.1	expressed protein	-2.621639183	0.03923989	Down
LOC_Os01g13710.1	transposon protein, putative, unclassified, expressed	-2.618968833	0.04027988	Down
LOC_Os04g42210.1	GPI-anchored protein, putative, expressed	-2.618212497	0.03940366	Down

Supplementary table S3 continued...

Gene ID	Putative functions	Log_2 fold change	P-Value	Regulation
LOC_Os09g10740.1	mitochondrial import inner membrane translocase subunit Tim17, putative, expressed	-2.617234264	0.04126151	Down
LOC_Os01g47740.1	zinc finger, C3HC4 type domain containing protein, expressed	-2.616944348	0.03955658	Down
LOC_Os02g32600.1	expressed protein	-2.613531653	0.04168528	Down
LOC_Os03g18360.1	BTBT1 - Bric-a-Brac, Tramtrack, Broad Complex BTB domain with tetratricopeptide rep	-2.612514731	0.04006133	Down
LOC_Os03g28090.1	pectinesterase, putative, expressed	-2.611732924	0.04014687	Down
LOC_Os01g56230.2	transmembrane protein 56, putative, expressed	-2.608982952	0.04071611	Down
LOC_Os02g32730.1	neutral/alkaline invertase, putative, expressed	-2.60641646	0.04065474	Down
LOC_Os03g24510.1	glycosyl transferase 8 domain containing protein, putative, expressed	-2.606240887	0.04047631	Down
LOC_Os02g31940.1	potassium transporter, putative, expressed	-2.605562347	0.04951691	Down
LOC_Os04g17479.1	expressed protein	-2.604544546	0.04317787	Down
LOC_Os03g50280.1	GLTP domain containing protein, putative, expressed	-2.604462371	0.04058556	Down
LOC_Os02g55500.1	expressed protein	-2.602790034	0.04192891	Down
LOC_Os10g26470.1	sucrose transporter, putativ, expressed	-2.602598626	0.04068462	Down
LOC_Os06g39050.1	syntaxin, putative, expressed	-2.602560535	0.04220665	Down
LOC_Os08g40590.2	oxysterol-binding protein, putative, expressed	-2.599508378	0.04103666	Down
LOC_Os02g55820.1	expressed protein	-2.599293448	0.04384223	Down
LOC_Os04g59610.1	expressed protein	-2.595993156	0.04626218	Down
LOC_Os02g50740.1	WD domain, G-beta repeat domain containing protein, expressed	-2.59487047	0.04911054	Down
LOC_Os06g43000.1	nitrate-induced NOI protein, expressed	-2.591861788	0.04280706	Down
LOC_Os02g09450.1	glycerophosphoryl diester phosphodiesterase family protein, putative, expressed	-2.589952039	0.04137964	Down
LOC_Os01g13440.1	pollen allergen Cyn d 23, putative, expressed	-2.58949912	0.04143092	Down
LOC_Os06g46560.1	myb-like DNA-binding domain containing protein, expressed	-2.588557416	0.04162602	Down
LOC_Os03g60470.1	glycine-rich protein A3, putative, expressed	-2.588498272	0.04194663	Down
LOC_Os06g03980.1	expressed protein	-2.587795066	0.04254156	Down
LOC_Os04g57350.1	EH domain-containing protein 1, putative, expressed	-2.584144512	0.04175797	Down
LOC_Os08g42750.1	CAMK_CAMK_like.37 - CAMK includes calcium/calmodulin depedent protein kinases, e	-2.583981156	0.04215671	Down
LOC_Os03g24170.1	phosphatidylinositol-4-phosphate 5-kinase, putative, expressed	-2.583571951	0.0425999	Down
LOC_Os06g48980.1	protein kinase APK1B, chloroplast precursor, putative, expressed	-2.582257667	0.04201778	Down
LOC_Os03g04560.1	expressed protein	-2.57972104	0.04227628	Down
LOC_Os07g17310.1	B12D protein, putative, expressed	-2.577596553	0.04259815	Down
LOC_Os02g58800.1	expressed protein	-2.576164271	0.04248327	Down
LOC_Os02g03510.1	fibroin heavy chain precursor, putative, expressed	-2.573246968	0.04262836	Down

Supplementary table S3 continued...

Gene ID	Putative functions	Log_2 fold change	P-Value	Regulation
LOC_Os09g32230.1	vignain precursor, putative, expressed	-2.570542473	0.04398531	Down
LOC_Os04g32190.1	expressed protein	-2.569672654	0.04353211	Down
LOC_Os07g47120.1	beta-amylase, putative, expressed	-2.568482177	0.04330108	Down
LOC_Os08g34910.1	pectinesterase, putative, expressed	-2.567816984	0.04300059	Down
LOC_Os04g41200.1	lipase, putative, expressed	-2.567495435	0.04338876	Down
LOC_Os10g33640.1	AGC_AGC_other_NDRh_TRCd.3 - ACG kinases include homologs to PKA, PKG and PKC,	-2.565604541	0.04385574	Down
LOC_Os02g12660.1	protein kinase domain containing protein, putative, expressed	-2.565170951	0.04347128	Down
LOC_Os11g08400.1	expressed protein	-2.564580387	0.04606892	Down
LOC_Os02g55330.2	OsPOP6 - Putative Prolyl Oligopeptidase homologue, expressed	-2.564142249	0.04518832	Down
LOC_Os05g11330.1	RALFL19 - Rapid ALkalinization Factor RALF family protein precursor, expressed	-2.562285725	0.04393234	Down
LOC_Os01g71780.1	WD domain, G-beta repeat domain containing protein, expressed	-2.562112987	0.04571501	Down
LOC_Os01g42024.1	expressed protein	-2.561111968	0.04414883	Down
LOC_Os08g40990.1	receptor-like protein kinase 1, putative, expressed	-2.560869431	0.04376964	Down
LOC_Os06g40500.1	expressed protein	-2.559294607	0.04608681	Down
LOC_Os02g36950.1	uncharacterized Cys-rich domain containing protein, putative, expressed	-2.558371132	0.04373131	Down
LOC_Os01g21970.1	protein kinase, putative, expressed	-2.553377923	0.04402587	Down
LOC_Os03g62210.1	subtilisin N-terminal Region family protein, expressed	-2.553096112	0.0445935	Down
LOC_Os02g32060.2	hydrolase, NUDIX family, domain containing protein, expressed	-2.552686098	0.04444138	Down
LOC_Os06g21390.1	Zinc finger C-x8-C-x5-C-x3-H type domain containing protein, expressed	-2.550231459	0.04466188	Down
LOC_Os11g05520.1	bifunctional monodehydroascorbate reductase and carbonic anhydrasenectarin-3 pre	-2.549947738	0.04556812	Down
LOC_Os06g48300.1	protein phosphatase 2C, putative, expressed	-2.549619619	0.04441324	Down
LOC_Os08g37570.1	spotted leaf 11, putative, expressed	-2.543071536	0.04591137	Down
LOC_Os04g21710.1	expressed protein	-2.540987681	0.04507868	Down
LOC_Os11g05970.1	pyridine nucleotide-disulphide oxidoreductase family protein, putative, expressed	-2.540918077	0.04661072	Down
LOC_Os06g45050.1	clathrin assembly protein, putative, expressed	-2.539532752	0.04553761	Down
LOC_Os06g45230.1	pollen allergen, putative, expressed	-2.538562511	0.04526783	Down
LOC_Os01g66860.2	serine/threonine protein kinase, putative, expressed	-2.538392599	0.04540989	Down
LOC_Os04g44820.1	zinc finger, C3HC4 type domain containing protein, expressed	-2.538144985	0.04563933	Down
LOC_Os01g11350.1	bZIP transcription factor domain containing protein, expressed	-2.534743858	0.04630883	Down
LOC_Os06g49890.1	coiled-coil domain-containing protein 90A, mitochondrial precursor, putative, express	-2.534300434	0.04604039	Down
LOC_Os12g06780.1	expressed protein	-2.534293832	0.04671171	Down
LOC_Os08g34340.1	DUF593 domain containing protein, expressed	-2.531115365	0.04624049	Down

Supplementary table S3 continued...

Gene ID	Putative functions	Log_2 fold change	P-Value	Regulation
LOC_Os07g49100.1	pectinesterase, putative, expressed	-2.531090729	0.04581058	Down
LOC_Os09g33910.1	CAMK_CAMK_like.39 - CAMK includes calcium/calmodulin depedent protein kinases, e	-2.529728108	0.0460306	Down
LOC_Os02g09530.1	expressed protein	-2.529436561	0.04656801	Down
LOC_Os03g49270.1	THION36 - Plant thionin family protein precursor, expressed	-2.529054521	0.04871678	Down
LOC_Os02g35300.1	transposon protein, putative, unclassified, expressed	-2.527622926	0.04614449	Down
LOC_Os03g52450.1	GATA transcription factor 25, putative, expressed	-2.52636286	0.04772738	Down
LOC_Os10g25674.1	mps one binder kinase activator-like 1A, putative, expressed	-2.52588201	0.04668049	Down
LOC_Os02g03500.1	expressed protein	-2.525609062	0.04646803	Down
LOC_Os03g62410.1	phospholipase D, putative, expressed	-2.52462148	0.04697437	Down
LOC_Os06g45200.1	group 3 pollen allergen, putative, expressed	-2.521098459	0.04874961	Down
LOC_Os09g30040.1	expressed protein	-2.520154032	0.04804542	Down
LOC_Os03g59440.1	dirigent, putative, expressed	-2.518656621	0.04713915	Down
LOC_Os05g38960.1	expressed protein	-2.517275693	0.04884974	Down
LOC_Os03g21880.1	THION28 - Plant thionin family protein precursor, expressed	-2.513631493	0.04743108	Down
LOC_Os02g49550.1	zinc finger, C3HC4 type domain containing protein, expressed	-2.510884644	0.04872182	Down
LOC_Os05g38790.1	IQ calmodulin-binding motif domain containing protein, expressed	-2.510635583	0.04882067	Down
LOC_Os03g45930.1	expressed protein	-2.510449342	0.04814897	Down
LOC_Os02g37580.1	fimbrin-like protein 2, putative, expressed	-2.51015429	0.04758968	Down
LOC_Os12g27190.1	expressed protein	-2.509531606	0.04799008	Down
LOC_Os02g49260.1	transporter-related, putative, expressed	-2.507792698	0.04819314	Down
LOC_Os11g06690.1	serine esterase family protein, putative, expressed	-2.50705903	0.0481994	Down
LOC_Os07g31830.1	GTPase activating protein, putative, expressed	-2.504764249	0.04837036	Down
LOC_Os10g37740.1	CGMC_GSK.9 - CGMC includes CDA, MAPK, GSK3, and CLKC kinases, expressed	-2.504472352	0.04804271	Down
LOC_Os07g30210.1	integral membrane protein DUF6 domain containing protein, expressed	-2.503388905	0.04858599	Down
LOC_Os02g49340.1	nitrate-induced NOI protein, putative, expressed	-2.502824848	0.04820337	Down
LOC_Os03g64290.1	myosin, putative, expressed	-2.501018491	0.0486358	Down
LOC_Os02g51730.1	dnaJ homolog subfamily C member 7, putative, expressed	-2.499310906	0.04995999	Down
LOC_Os03g45280.1	dehydrin, putative, expressed	-2.496625986	0.04936715	Down
LOC_Os08g33860.1	zinc finger, C3HC4 type, putative, expressed	-2.496398362	0.04875639	Down
LOC_Os11g37280.1	LTPL68 - Protease inhibitor/seed storage/LTP family protein precursor, expressed	2.515329791	0.04846772	Up
LOC_Os01g09220.1	transposon protein, putative, CACTA, En/Spm sub-class, expressed	2.587387326	0.04849889	Up
LOC_Os04g44330.1	transposon protein, putative, unclassified, expressed	2.608951799	0.04902593	Up

Supplementary table S3 continued...

Gene ID	Putative functions	Log_2 fold change	P-Value	Regulation
LOC_Os05g02070.2	expressed protein	2.611572472	0.04729402	Up
LOC_Os11g02389.1	protease inhibitor/seed storage/LTP family, putative, expressed	2.632317824	0.04218856	Up
LOC_Os02g44230.1	CPuORF22 - conserved peptide uORF-containing transcript, expressed	2.703744658	0.04945582	Up
LOC_Os02g10490.1	expressed protein	2.714874006	0.04540848	Up
LOC_Os03g47620.1	ankyrin, putative, expressed	2.759581559	0.04802317	Up
LOC_Os12g03094.1	expressed protein	2.770694139	0.03442052	Up
LOC_Os12g44010.1	purple acid phosphatase precursor, putative, expressed	2.796456008	0.02941293	Up
LOC_Os12g02320.1	LTPL12 - Protease inhibitor/seed storage/LTP family protein precursor, expressed	2.798208868	0.03340959	Up
LOC_Os08g15505.1	expressed protein	2.799633211	0.03216558	Up
LOC_Os02g51110.1	aquaporin protein, putative, expressed	2.80420128	0.04354153	Up
LOC_Os03g03680.1	transporter family protein, putative, expressed	2.828036931	0.03008751	Up
LOC_Os12g37519.2	retrotransposon protein, putative, unclassified, expressed	2.838848545	0.03243563	Up
LOC_Os11g14160.1	transposon protein, putative, Pong sub-class, expressed	2.839123556	0.0458446	Up
LOC_Os10g42960.1	urea active transporter, putative, expressed	2.847442131	0.03643803	Up
LOC_Os06g48030.1	peroxidase precursor, putative, expressed	2.885730125	0.04139099	Up
LOC_Os04g35540.1	amino acid permease family protein, putative, expressed	2.88654195	0.03688795	Up
LOC_Os04g43910.1	auxin response factor, putative, expressed	2.905885083	0.03138178	Up
LOC_Os02g35660.1	PTF1, putative, expressed	2.965871322	0.03014628	Up
LOC_Os03g58900.1	galactosyltransferase family protein, putative, expressed	2.97735916	0.02580439	Up
LOC_Os04g43440.1	NB-ARC/LRR disease resistance protein, putative, expressed	2.987093249	0.04131071	Up
LOC_Os01g12190.1	expressed protein	3.020826668	0.03435116	Up
LOC_Os10g30280.1	OsFBX386 - F-box domain containing protein, expressed	3.024542184	0.04982437	Up
LOC_Os04g45450.1	expressed protein	3.055271975	0.04001005	Up
LOC_Os05g03100.1	HECT-domain domain containing protein, expressed	3.079187028	0.04447734	Up
LOC_Os08g14364.1	expressed protein	3.080791407	0.03858349	Up
LOC_Os03g30950.1	acyl-desaturase, chloroplast precursor, putative, expressed	3.103995322	0.02462459	Up
LOC_Os08g38330.1	OsFBX297 - F-box domain containing protein, expressed	3.151716361	0.048547	Up
LOC_Os11g44340.1	calmodulin binding protein, putative, expressed	3.159560445	0.0272771	Up
LOC_Os01g27160.1	cullin, putative, expressed	3.163736757	0.0186621	Up
LOC_Os06g46950.1	EF hand family protein, putative, expressed	3.173064709	0.02413444	Up
LOC_Os01g06140.1	expressed protein	3.176780945	0.03261368	Up
LOC_Os11g24070.1	LTPL10 - Protease inhibitor/seed storage/LTP family protein precursor, expressed	3.197498927	0.04024674	Up

Supplementary table S3 continued...

Gene ID	Putative functions	Log_2 fold change	P-Value	Regulation
LOC_Os05g14590.1	MCM6 - Putative minichromosome maintenance MCM complex subunit 6, expressed	3.202556006	0.02579886	Up
LOC_Os01g61610.2	flavonol synthase/flavanone 3-hydroxylase, putative, expressed	3.207157908	0.03044833	Up
LOC_Os08g29770.1	endoglucanase, putative, expressed	3.210242348	0.01344943	Up
LOC_Os02g52040.1	phosphate-induced protein 1 conserved region domain containing protein, expressed	3.217223175	0.02057633	Up
LOC_Os06g48160.1	glycosyl hydrolases family 16, putative, expressed	3.218142948	0.03387238	Up
LOC_Os08g03682.1	cytochrome P450, putative, expressed	3.221209157	0.03926715	Up
LOC_Os01g60420.1	expressed protein	3.257467079	0.04195586	Up
LOC_Os09g13890.1	calmodulin binding protein, putative, expressed	3.264881595	0.03418441	Up
LOC_Os09g22160.1	expressed protein	3.285468157	0.04197465	Up
LOC_Os03g13740.1	immediate-early fungal elicitor protein CMPG1, putative, expressed	3.286417333	0.02275546	Up
LOC_Os05g50640.1	transposon protein, putative, unclassified, expressed	3.288103217	0.02450448	Up
LOC_Os06g06890.1	expressed protein	3.29234617	0.01571137	Up
LOC_Os06g17730.1	plastocyanin-like domain containing protein, putative, expressed	3.307605193	0.01672004	Up
LOC_Os04g33660.2	bifunctional monodehydroascorbate reductase and carbonic anhydrasenectarin-3 pre	3.32091745	0.01493796	Up
LOC_Os06g12210.1	helix-loop-helix DNA-binding domain containing protein, expressed	3.32442195	0.03799272	Up
LOC_Os06g04990.1	early nodulin 93 ENOD93 protein, putative, expressed	3.347806727	0.04692838	Up
LOC_Os12g29400.1	GRAM domain containing protein, expressed	3.362970328	0.02456982	Up
LOC_Os12g38300.1	metallothionein, putative, expressed	3.363748271	0.03350885	Up
LOC_Os03g48220.1	expressed protein	3.424814397	0.01408065	Up
LOC_Os08g43020.1	transferase family protein, putative, expressed	3.46771619	0.03751833	Up
LOC_Os05g41880.1	mutS domain V family protein, expressed	3.478937208	0.04381786	Up
LOC_Os02g10520.1	OsSub12 - Putative Subtilisin homologue, expressed	3.48733633	0.02140082	Up
LOC_Os08g03470.1	MBTB15 - Bric-a-Brac, Tramtrack, Broad Complex BTB domain with Meprin and TRAF H	3.489722309	0.03017692	Up
LOC_Os09g32360.1	gp176, putative, expressed	3.490226425	0.01790112	Up
LOC_Os09g36900.1	WD domain, G-beta repeat domain containing protein, expressed	3.495538143	0.04048817	Up
LOC_Os01g07530.1	uncharacterized glycosyltransferase, putative, expressed	3.500502148	0.0367118	Up
LOC_Os07g06620.2	YABBY domain containing protein, putative, expressed	3.511623478	0.0251268	Up
LOC_Os03g60560.1	ZOS3-21 - C2H2 zinc finger protein, expressed	3.513688898	0.03737838	Up
LOC_Os01g51620.1	KRR1 small subunit processome component, putative, expressed	3.516781493	0.01950854	Up
LOC_Os10g18400.1	beta-galactosidase precursor, putative, expressed	3.519783777	0.00770168	Up
LOC_Os04g43800.1	phenylalanine ammonia-lyase, putative, expressed	3.532145802	0.01130177	Up
LOC_Os03g11600.1	YABBY domain containing protein, putative, expressed	3.536418649	0.02178463	Up

Supplementary table S3 continued...

Gene ID	Putative functions	Log_2 fold change	P-Value	Regulation
LOC_Os03g31560.1	retrotransposon protein, putative, unclassified, expressed	3.539905226	0.03907456	Up
LOC_Os08g14660.1	SET domain containing protein, expressed	3.540626033	0.03716993	Up
LOC_Os11g41360.1	expressed protein	3.559263115	0.00856375	Up
LOC_Os09g37850.1	retrotransposon protein, putative, unclassified, expressed	3.569217389	0.02857356	Up
LOC_Os02g33820.1	abscisic stress-ripening, putative, expressed	3.573466862	0.03860747	Up
LOC_Os04g28580.1	transposon protein, putative, unclassified, expressed	3.573466862	0.01939202	Up
LOC_Os06g14780.1	expressed protein	3.576857451	0.03835905	Up
LOC_Os11g44310.1	calmodulin binding protein, putative, expressed	3.607572072	0.01483962	Up
LOC_Os03g61850.1	transposon protein, putative, unclassified, expressed	3.610841133	0.02842969	Up
LOC_Os04g37700.1	expressed protein	3.612976877	0.03784773	Up
LOC_Os12g27102.1	glycerophosphoryl diester phosphodiesterase family protein, putative, expressed	3.62364069	0.00778117	Up
LOC_Os03g38010.1	nuf2 family protein, expressed	3.662965013	0.04859888	Up
LOC_Os03g51230.2	SNF2 family N-terminal domain containing protein, expressed	3.664453094	0.01520015	Up
LOC_Os08g40010.1	expressed protein	3.666817338	0.03057552	Up
LOC_Os10g05720.1	LTPL37 - Protease inhibitor/seed storage/LTP family protein precursor, expressed	3.676535712	0.01714409	Up
LOC_Os01g18870.1	helix-loop-helix DNA-binding domain containing protein, expressed	3.691042875	0.00568018	Up
LOC_Os04g33720.1	glycosyl hydrolases, putative, expressed	3.711058835	0.00555006	Up
LOC_Os03g47530.1	glycosyl transferase 8 domain containing protein, putative, expressed	3.7206496	0.03735411	Up
LOC_Os01g03390.1	BBTI7 - Bowman-Birk type bran trypsin inhibitor precursor, expressed	3.727879162	0.00558714	Up
LOC_Os01g55820.1	X8 domain containing protein, expressed	3.738809294	0.02518725	Up
LOC_Os11g38640.1	expressed protein	3.752040915	0.02723808	Up
LOC_Os01g47930.1	hypothetical protein	3.798549025	0.00946853	Up
LOC_Os04g32620.1	ethylene-responsive transcription factor ERF114, putative, expressed	3.810149941	0.04436554	Up
LOC_Os10g17260.1	cytochrome P450, putative, expressed	3.821169088	0.0080511	Up
LOC_Os03g05610.1	inorganic phosphate transporter, putative, expressed	3.829013803	0.00989855	Up
LOC_Os05g12710.1	expressed protein	3.833233554	0.04247432	Up
LOC_Os03g53790.1	periplasmic beta-glucosidase precursor, putative, expressed	3.840125397	0.00400392	Up
LOC_Os10g13960.1	retrotransposon protein, putative, unclassified, expressed	3.851438149	0.0410299	Up
LOC_Os03g30810.1	retrotransposon protein, putative, unclassified, expressed	3.857980995	0.02329557	Up
LOC_Os10g13370.1	retrotransposon protein, putative, unclassified, expressed	3.862850035	0.03320377	Up
LOC_Os09g35700.1	LTPL45 - Protease inhibitor/seed storage/LTP family protein precursor, expressed	3.876878027	0.01075114	Up
LOC_Os04g51460.1	glycosyl hydrolases family 16, putative, expressed	3.908328259	0.00740448	Up

Supplementary table S3 continued...

Gene ID	Putative functions	Log_2 fold change	P-Value	Regulation
LOC_Os12g41890.1	amino acid permease family protein, putative, expressed	3.920356663	0.00555983	Up
LOC_Os12g42200.1	ATCHX, putative, expressed	3.922521247	0.02326093	Up
LOC_Os08g04560.1	decarboxylase, putative, expressed	3.926660978	0.00758001	Up
LOC_Os01g43750.1	cytochrome P450 72A1, putative, expressed	3.930630317	0.03520987	Up
LOC_Os07g35140.1	receptor-like serine-threonine protein kinase, putative, expressed	3.994202155	0.03928954	Up
LOC_Os05g15300.1	expressed protein	3.995117779	0.00435171	Up
LOC_Os09g25290.1	methyladenine glycosylase, putative, expressed	4.024542184	0.01880542	Up
LOC_Os03g63540.1	lysine-rich arabinogalactan protein 19 precursor, putative, expressed	4.038381092	0.0232927	Up
LOC_Os03g07250.1	cytochrome P450, putative, expressed	4.066588081	0.00853503	Up
LOC_Os01g38670.1	transporter family protein, putative, expressed	4.07862638	0.00313868	Up
LOC_Os03g26430.1	aldose 1-epimerase, putative, expressed	4.081613766	0.00265343	Up
LOC_Os05g02760.1	expressed protein	4.093602718	0.00630261	Up
LOC_Os04g12830.1	expressed protein	4.114883638	0.03098536	Up
LOC_Os03g06000.1	expansin precursor, putative, expressed	4.119435231	0.00607332	Up
LOC_Os11g05760.1	OsProCP5 - Putative Lysosomal Pro-x Carboxypeptidase homologue, expressed	4.119667717	0.00235802	Up
LOC_Os06g05470.1	expressed protein	4.121417002	0.01649731	Up
LOC_Os07g37400.1	OsFBX257 - F-box domain containing protein, expressed	4.126482573	0.00963337	Up
LOC_Os09g32840.1	nucleotide pyrophosphatase/phosphodiesterase, putative, expressed	4.144267445	0.00718715	Up
LOC_Os08g43240.1	LTPL97 - Protease inhibitor/seed storage/LTP family protein precursor, expressed	4.152306149	0.00666458	Up
LOC_Os04g45420.1	retrotransposon protein, putative, unclassified, expressed	4.153328491	0.0167251	Up
LOC_Os08g10500.1	expressed protein	4.157297393	0.01160209	Up
LOC_Os03g45120.1	ribosome inactivating protein, putative, expressed	4.198689024	0.00570074	Up
LOC_Os04g52504.1	adhesive/proline-rich protein, putative, expressed	4.204649599	0.00406806	Up
LOC_Os02g02640.1	NBS-LRR disease resistance protein, putative, expressed	4.211745177	0.04149153	Up
LOC_Os01g66290.1	OsMADS21 - MADS-box family gene with MIKCc type-box, expressed	4.234465254	0.04890332	Up
LOC_Os12g23980.1	OsSub63 - Putative Subtilisin homologue, expressed	4.260000346	0.0466209	Up
LOC_Os03g50670.1	retrotransposon protein, putative, Ty3-gypsy subclass, expressed	4.26611088	0.00582495	Up
LOC_Os09g32370.1	gp176, putative, expressed	4.271123724	0.01189404	Up
LOC_Os09g29600.1	OsWAK85 - OsWAK receptor-like cytoplasmic kinase OsWAK-RLCK, expressed	4.295161185	0.03526409	Up
LOC_Os09g06560.1	WD domain, G-beta repeat domain containing protein, expressed	4.299015569	0.00602342	Up
LOC_Os03g22470.1	desiccation-related protein PCC13-62 precursor, putative, expressed	4.339999668	0.04001641	Up
LOC_Os02g45530.1	HOTHEAD precursor, putative, expressed	4.357847669	0.03865161	Up

Supplementary table S3 continued...

Gene ID	Putative functions	Log_2 fold change	P-Value	Regulation
LOC_Os04g32080.1	11-beta-hydroxysteroid dehydrogenase, putative, expressed	4.363748271	0.01611344	Up
LOC_Os07g11739.1	cytochrome P450, putative, expressed	4.375477563	0.03734095	Up
LOC_Os03g04060.1	CHIT16 - Chitinase family protein precursor, expressed	4.384212373	0.04722873	Up
LOC_Os11g12590.1	expressed protein	4.39937218	0.02858859	Up
LOC_Os01g45250.1	DUF1645 domain containing protein, putative, expressed	4.423499078	0.00514487	Up
LOC_Os01g52690.1	retrotransposon protein, putative, unclassified, expressed	4.429925347	0.01100356	Up
LOC_Os02g01190.1	POEI25 - Pollen Ole e I allergen and extensin family protein precursor, expressed	4.431870992	0.0122535	Up
LOC_Os05g10780.1	aminotransferase, classes I and II, domain containing protein, expressed	4.457225662	0.0033337	Up
LOC_Os03g11540.1	RPA1B - Putative single-stranded DNA binding complex subunit 1, expressed	4.469681715	0.00571674	Up
LOC_Os03g35750.1	expressed protein	4.482392767	0.03913377	Up
LOC_Os04g28570.1	fatty acyl coA reductase, putative, expressed	4.491127577	0.0107178	Up
LOC_Os07g34520.1	isocitrate lyase, putative, expressed	4.49777793	0.00396563	Up
LOC_Os03g06180.1	expressed protein	4.505928282	0.02289517	Up
LOC_Os01g67820.1	exo70 exocyst complex subunit domain containing protein, expressed	4.512450001	0.00822439	Up
LOC_Os11g08460.1	DnaK family protein, putative, expressed	4.540852498	0.00484073	Up
LOC_Os01g08800.1	cytochrome P450, putative, expressed	4.556393349	0.00377304	Up
LOC_Os08g13250.1	speckle-type POZ protein, putative, expressed	4.556393349	0.04626074	Up
LOC_Os08g27870.1	EARLY flowering protein, putative, expressed	4.559668481	0.01195446	Up
LOC_Os08g43290.1	LTPL44 - Protease inhibitor/seed storage/LTP family protein precursor, expressed	4.569449501	0.01169189	Up
LOC_Os03g14630.1	LTPL106 - Protease inhibitor/seed storage/LTP family protein precursor, expressed	4.571500241	0.00513407	Up
LOC_Os07g48550.1	no apical meristem protein, putative, expressed	4.589307971	0.0085528	Up
LOC_Os01g72900.1	abscisic stress-ripening, putative, expressed	4.592017258	0.00331628	Up
LOC_Os09g24840.1	GASR10 - Gibberellin-regulated GASA/GAST/Snakin family protein precursor, expresse	4.626782676	0.01201951	Up
LOC_Os03g04070.1	no apical meristem protein, putative, expressed	4.626782676	0.01025438	Up
LOC_Os03g25150.1	transposon protein, putative, unclassified, expressed	4.633636347	0.03988656	Up
LOC_Os10g42210.1	enoyl-CoA-hydratase, putative, expressed	4.646381854	0.00167139	Up
LOC_Os05g46480.1	late embryogenesis abundant protein, group 3, putative, expressed	4.655127302	0.02764397	Up
LOC_Os10g34360.1	stilbene synthase, putative, expressed	4.666503105	0.00246633	Up
LOC_Os04g44600.1	CRP1 - Cysteine-rich family protein precursor, expressed	4.667776152	0.00408564	Up
LOC_Os08g20410.1	expressed protein	4.678129556	0.00312154	Up
LOC_Os11g40210.1	remorin C-terminal domain containing protein, putative, expressed	4.684498174	0.01970082	Up
LOC_Os03g60570.1	ZOS3-22 - C2H2 zinc finger protein, expressed	4.687324218	0.00545508	Up

Supplementary table S3 continued...

Gene ID	Putative functions	Log_2 fold change	P-Value	Regulation
LOC_Os05g39250.1	phosphatidylethanolamine-binding protein, putative, expressed	4.699351302	0.0252051	Up
LOC_Os06g40520.1	TNP1, putative, expressed	4.72631835	0.01797962	Up
LOC_Os01g19770.1	mitochondrial import inner membrane translocase subunit Tim17, putative, expressed	4.762500686	0.01659733	Up
LOC_Os04g22430.1	retrotransposon protein, putative, unclassified, expressed	4.768443826	0.02175965	Up
LOC_Os02g11859.1	expressed protein	4.772711255	0.00370183	Up
LOC_Os11g14150.1	transposon protein, putative, Pong sub-class, expressed	4.773624065	0.0026208	Up
LOC_Os06g37740.1	expressed protein	4.781359714	0.04463103	Up
LOC_Os01g62740.1	expressed protein	4.802149763	0.01518994	Up
LOC_Os10g12500.1	integral membrane protein DUF6 containing protein, expressed	4.802149763	0.01518994	Up
LOC_Os04g58080.1	polygalacturonase inhibitor 3 precursor, putative, expressed	4.810814624	0.01489669	Up
LOC_Os07g40740.1	heparanase-like protein precursor, putative, expressed	4.81407452	0.00116748	Up
LOC_Os03g12730.1	receptor protein kinase CLAVATA1 precursor, putative, expressed	4.826922291	0.00136296	Up
LOC_Os09g21240.1	expressed protein	4.85622411	0.00184222	Up
LOC_Os01g48800.1	purine permease, putative, expressed	4.858956119	0.01786124	Up
LOC_Os03g56480.1	expressed protein	4.913945353	0.00210193	Up
LOC_Os03g08284.3	expressed protein	4.918161708	0.02225193	Up
LOC_Os01g72910.1	abscisic stress-ripening, putative, expressed	4.985531045	0.0099583	Up
LOC_Os04g59430.1	auxin response factor, putative, expressed	5.021061616	0.00471031	Up
LOC_Os04g54450.1	retrotransposon protein, putative, unclassified, expressed	5.031478231	0.01735865	Up
LOC_Os01g53550.1	expressed protein	5.035365154	0.02683074	Up
LOC_Os06g09980.1	expressed protein	5.092446249	0.00224489	Up
LOC_Os10g42220.1	enoyl-CoA hydratase/isomerase family protein, putative, expressed	5.107762877	0.0012227	Up
LOC_Os10g28240.1	calcium-transporting ATPase, plasma membrane-type, putative, expressed	5.11263156	0.00038827	Up
LOC_Os11g44030.1	expressed protein	5.123208503	0.02225295	Up
LOC_Os06g44240.1	gp176, putative, expressed	5.126248957	0.00168582	Up
LOC_Os10g32760.1	zinc finger family protein, putative, expressed	5.157297393	0.03699324	Up
LOC_Os09g20250.1	retrotransposon protein, putative, Ty1-copia subclass, expressed	5.159937275	0.00131187	Up
LOC_Os02g40840.1	alcohol oxidase, putative, expressed	5.180884213	0.00383079	Up
LOC_Os08g02996.1	receptor-like kinase, putative, expressed	5.211745177	0.01832608	Up
LOC_Os04g55660.1	GDSL-like lipase/acylhydrolase, putative, expressed	5.224375298	0.00023156	Up
LOC_Os01g66930.2	transposon protein, putative, CACTA, En/Spm sub-class, expressed	5.224771857	0.00557979	Up
LOC_Os06g36010.1	plastocyanin-like domain containing protein, putative, expressed	5.296789848	0.00030221	Up

Supplementary table S3 continued...

Gene ID	Putative functions	Log_2 fold change	P-Value	Regulation
LOC_Os09g25430.1	ZOS9-07 - C2H2 zinc finger protein, expressed	5.321928095	0.02640597	Up
LOC_Os07g46210.1	LTPL2 - Protease inhibitor/seed storage/LTP family protein precursor, expressed	5.34298971	0.01360543	Up
LOC_Os03g21960.1	aminotransferase, putative, expressed	5.385904684	0.00035361	Up
LOC_Os08g23210.1	retrotransposon protein, putative, unclassified, expressed	5.463283944	0.04612053	Up
LOC_Os03g06100.1	retrotransposon protein, putative, unclassified, expressed	5.510589659	0.04212375	Up
LOC_Os04g55380.1	expressed protein	5.527886921	0.00038195	Up
LOC_Os10g24800.1	hypothetical protein	5.609504685	0.0346521	Up
LOC_Os01g72530.1	OsCML31 - Calmodulin-related calcium sensor protein, expressed	5.609504685	0.00158346	Up
LOC_Os07g43560.1	TKL_IRAK_DUF26-lc.24 - DUF26 kinases have homology to DUF26 containing loci, expr	5.626782676	0.01341052	Up
LOC_Os05g19050.1	retrotransposon protein, putative, unclassified, expressed	5.660730008	0.00631384	Up
LOC_Os01g12070.1	endoglucanase precursor, putative, expressed	5.677408749	0.01190666	Up
LOC_Os07g22850.1	chalcone and stilbene synthases, putative, expressed	5.710198685	0.0033188	Up
LOC_Os02g04130.1	DUF1645 domain containing protein, putative, expressed	5.717129179	0.00155865	Up
LOC_Os07g39020.1	OsSub53 - Putative Subtilisin homologue, expressed	5.718281031	0.02770907	Up
LOC_Os04g13000.1	retrotransposon protein, putative, LINE subclass, expressed	5.734311141	0.02678997	Up
LOC_Os04g43870.1	transposon protein, putative, CACTA, En/Spm sub-class, expressed	5.734311141	0.02678997	Up
LOC_Os11g02290.1	expressed protein	5.79160981	0.00177518	Up
LOC_Os02g39470.1	cyclin, N-terminal domain containing protein, expressed	5.885516945	0.01929983	Up
LOC_Os04g52190.1	vacuolar-sorting receptor precursor, putative, expressed	5.902168185	0.00687816	Up
LOC_Os04g26550.1	expressed protein	5.909608048	0.00012843	Up
LOC_Os02g18690.1	BURP domain containing protein, expressed	5.9395216	0.00625902	Up
LOC_Os01g15740.1	expressed protein	6.002649578	0.01479298	Up
LOC_Os01g06680.1	retrotransposon protein, putative, unclassified, expressed	6.108934372	0.00403818	Up
LOC_Os02g34120.1	expressed protein	6.159277757	0.01020451	Up
LOC_Os04g43840.1	possible lysine decarboxylase domain containing protein, expressed	6.159277757	0.01020451	Up
LOC_Os03g27830.1	expressed protein	6.166515923	0.00027764	Up
LOC_Os05g11110.1	retrotransposon protein, putative, unclassified, expressed	6.188661564	0.00032642	Up
LOC_Os01g58970.1	cytochrome P450, putative, expressed	6.196948175	0.00041742	Up
LOC_Os08g41570.1	expressed protein	6.22314994	0.00872699	Up
LOC_Os01g55160.1	expressed protein	6.240089803	0.00836844	Up
LOC_Os01g05050.1	mitochondrial glycoprotein, putative, expressed	6.256833067	0.04511379	Up
LOC_Os11g18730.1	glycosyl hydrolase family 3 protein, putative, expressed	6.289747689	0.04235869	Up

Supplementary table S3 continued...

Gene ID	Putative functions	Log_2 fold change	P-Value	Regulation
LOC_Os12g19580.1	photosynthetic reaction center protein, putative, expressed	6.289747689	0.00248597	Up
LOC_Os03g39040.1	zinc knuckle domain containing protein, expressed	6.289747689	0.04235869	Up
LOC_Os08g26350.1	expressed protein	6.31483867	0.0023211	Up
LOC_Os06g46799.1	peroxidase precursor, putative, expressed	6.327222395	0.00040267	Up
LOC_Os06g15430.1	expressed protein	6.374016606	0.0359044	Up
LOC_Os02g31850.1	transposon protein, putative, unclassified, expressed	6.374016606	0.00197193	Up
LOC_Os03g49360.1	expressed protein	6.414374344	0.03310421	Up
LOC_Os01g43980.1	retrotransposon protein, putative, unclassified, expressed	6.414374344	0.03310421	Up
LOC_Os12g16720.1	cytochrome P450 71A1, putative, expressed	6.441479574	0.0007651	Up
LOC_Os10g34130.1	nodulin, putative, expressed	6.463283944	0.02994906	Up
LOC_Os07g05430.1	retrotransposon protein, putative, unclassified, expressed	6.538246002	0.0255914	Up
LOC_Os02g34030.1	retrotransposon protein, putative, unclassified, expressed	6.592017258	0.00049631	Up
LOC_Os04g24530.1	AMP-binding domain containing protein, expressed	6.677408749	8.323E-05	Up
LOC_Os05g25380.1	retrotransposon, putative, centromere-specific	6.804320862	0.01412868	Up
LOC_Os01g04160.1	expressed protein	6.856517073	0.01249372	Up
LOC_Os06g42420.1	transposon protein, putative, unclassified, expressed	6.856517073	0.01249372	Up
LOC_Os11g43790.1	DUF581 domain containing protein, expressed	6.885516945	0.01165817	Up
LOC_Os03g37830.1	potassium transporter, putative, expressed	6.922365776	0.00010975	Up
LOC_Os01g07660.1	expressed protein	6.982658103	0.00920339	Up
LOC_Os05g05920.1	desiccation-related protein PCC13-62 precursor, putative, expressed	7.015824967	0.00847617	Up
LOC_Os11g10090.1	transposon protein, putative, CACTA, En/Spm sub-class, expressed	7.045778189	1.4409E-05	Up
LOC_Os01g01660.1	isoflavone reductase, putative, expressed	7.113027126	1.0218E-05	Up
LOC_Os11g26570.1	dehydrin, putative, expressed	7.234465254	0.00483174	Up
LOC_Os10g30670.1	transposon protein, putative, CACTA, En/Spm sub-class, expressed	7.247555253	0.00015445	Up
LOC_Os03g59330.1	polygalacturonase, putative, expressed	7.337753062	0.00366381	Up
LOC_Os11g31190.1	nodulin MtN3 family protein, putative, expressed	7.424289813	0.00289071	Up
LOC_Os08g27170.1	calmodulin binding protein, putative, expressed	7.426758068	4.2282E-05	Up
LOC_Os06g47730.1	retrotransposon protein, putative, Ty1-copia subclass, expressed	7.542804284	0.00207446	Up
LOC_Os01g54430.1	plastocyanin-like domain containing protein, putative, expressed	7.565382132	0.00194563	Up
LOC_Os11g37960.1	WIP4 - Wound-induced protein precursor, expressed	7.583193408	0.00184931	Up
LOC_Os03g02470.1	expressed protein	7.58981635	0.00018215	Up
LOC_Os03g63870.1	expressed protein	7.608055468	5.1862E-05	Up

Supplementary table S3 continued...

Gene ID	Putative functions	Log_2 fold change	P-Value	Regulation
LOC_Os01g65300.1	retrotransposon protein, putative, unclassified, expressed	7.68154848	0.00139293	Up
LOC_Os01g33670.1	expressed protein	7.871995805	1.1327E-05	Up
LOC_Os10g02500.1	serine/threonine-protein kinase BRI1-like 2 precursor, putative, expressed	7.906890596	0.00071499	Up
LOC_Os01g65290.1	retrotransposon protein, putative, unclassified, expressed	8.220307191	0.00027355	Up
LOC_Os10g25850.1	nuclear transcription factor Y subunit, putative, expressed	8.256833067	0.00024409	Up
LOC_Os11g34110.1	heparan-alpha-glucosaminide N-acetyltransferase, putative, expressed	8.567620604	8.8007E-06	Up
LOC_Os09g37780.1	serine/threonine-protein kinase receptor precursor, putative, expressed	8.68051466	6.2203E-06	Up
LOC_Os01g64470.1	harpin-induced protein 1 domain containing protein, expressed	8.687735888	6.2344E-05	Up
LOC_Os09g17000.1	glycerophosphoryl diester phosphodiesterase family protein, putative, expressed	8.789054105	4.5074E-05	Up
LOC_Os07g28160.1	cytochrome P450 51, putative, expressed	9.317944581	8.2848E-06	Up
LOC_Os02g55649.1	CXXXC8 - Cysteine-rich protein with paired CXXXC motifs precursor, putative, expresse	inf	5.2196E-09	Down
LOC_Os02g09200.1	cytochrome P450 71D10, putative, expressed	inf	0.02596598	Down
LOC_Os02g55698.1	expressed protein	inf	2.3318E-13	Down
LOC_Os05g32420.1	expressed protein	inf	0.0106614	Down
LOC_Os12g27810.1	OsFBX444 - F-box domain containing protein, expressed	inf	0.04834693	Down
LOC_Os05g49700.1	AP2 domain containing protein, expressed	Inf	0.01525115	Up
LOC_Os03g42230.1	B3 DNA binding domain containing protein, expressed	Inf	0.00221245	Up
LOC_Os02g27940.1	calcineurin B, putative	Inf	0.03708999	Up
LOC_Os07g41060.1	dihydroflavonol-4-reductase, putative, expressed	Inf	0.04945693	Up
LOC_Os04g46650.1	expansin precursor, putative, expressed	Inf	0.02106148	Up
LOC_Os07g05910.1	expressed protein	Inf	0.03805486	Up
LOC_Os09g33540.1	expressed protein	Inf	0.02813193	Up
LOC_Os11g44000.1	expressed protein	Inf	0.00754456	Up
LOC_Os02g25640.1	glutelin, putative, expressed	Inf	0.03266585	Up
LOC_Os08g03410.1	glutelin, putative, expressed	Inf	0.01120964	Up
LOC_Os01g50910.1	late embryogenesis abundant protein, group 3, putative, expressed	Inf	0.01559894	Up
LOC_Os05g30580.1	OsSub46 - Putative Subtilisin homologue, expressed	Inf	0.02955806	Up
LOC_Os07g10580.1	PROLM26 - Prolamin precursor, expressed	Inf	0.04689497	Up
LOC_Os01g37580.1	retrotransposon protein, putative, unclassified, expressed	Inf	0.03266585	Up
LOC_Os07g12260.1	retrotransposon protein, putative, unclassified, expressed	Inf	1.5078E-05	Up
LOC_Os08g03080.1	retrotransposon protein, putative, unclassified, expressed	Inf	0.0274485	Up
LOC_Os05g43630.1	transposon protein, putative, Pong sub-class, expressed	Inf	0.01007661	Up

Supplementary table S3 continued...

Gene ID	Putative functions	Log_2 fold change	P-Value	Regulation
LOC_Os06g42520.1	transposon protein, putative, unclassified, expressed	Inf	0.03266585	Up
LOC_Os01g46270.1	wax synthase isoform 3, putative, expressed	Inf	0.01222257	Up
LOC_Os02g44090.1	zinc finger protein, putative, expressed	Inf	0.04333054	Up

Supplementary table S4: Carbohydrate metabolism related genes differentially expressed in *bHLH142* ^{OE} compared to wild type.

Carbohydrate Metabolism_MP				
**Gene ID**	**Putative functions**	**Log_2 fold change**	**P-Value**	**Regulation**
LOC_Os03g26430.1	aldose 1-epimerase, putative, expressed	4.081613766	0.00265343	Up
LOC_Os07g47120.1	beta-amylase, putative, expressed	-2.568482177	0.04330108	Down
LOC_Os10g18400.1	beta-galactosidase precursor, putative, expressed	3.519783777	0.00770168	Up
LOC_Os02g44230.1	CPuORF22 - conserved peptide uORF-containing transcript, expressed	2.703744658	0.04945582	Up
LOC_Os01g12070.1	endoglucanase precursor, putative, expressed	5.677408749	0.01190666	Up
LOC_Os08g29770.1	endoglucanase, putative, expressed	3.210242348	0.01344943	Up
LOC_Os09g36060.1	endoglucanase, putative, expressed	-3.133574766	0.0318164	Down
LOC_Os06g28194.1	expressed protein	-3.046902478	0.04085012	Down
LOC_Os02g55820.1	expressed protein	-2.599293448	0.04384223	Down
LOC_Os10g08022.1	fructose-bisphospate aldolase isozyme, putative, expressed	-2.646373677	0.03754915	Down
LOC_Os03g58900.1	galactosyltransferase family protein, putative, expressed	2.97735916	0.02580439	Up
LOC_Os07g13580.1	glucan endo-1,3-beta-glucosidase precursor, putative, expressed	-2.670295436	0.03623573	Down
LOC_Os05g50380.1	glucose-1-phosphate adenylyltransferase large subunit, chloroplast precursor	-2.679884012	0.03556687	Down
LOC_Os09g17000.1	glycerophosphoryl diester phosphodiesterase family protein, putative, expres	8.789054105	4.5074E-05	Up
LOC_Os12g27102.1	glycerophosphoryl diester phosphodiesterase family protein, putative, expres	3.62364069	0.00778117	Up
LOC_Os08g42390.1	glycerophosphoryl diester phosphodiesterase family protein, putative, expres	-2.711949175	0.03546068	Down
LOC_Os02g09450.1	glycerophosphoryl diester phosphodiesterase family protein, putative, expres	-2.589952039	0.04137964	Down
LOC_Os11g18730.1	glycosyl hydrolase family 3 protein, putative, expressed	6.289747689	0.04235869	Up
LOC_Os04g33720.1	glycosyl hydrolases, putative, expressed	3.711058835	0.00555006	Up
LOC_Os03g47530.1	glycosyl transferase 8 domain containing protein, putative, expressed	3.7206496	0.03735411	Up
LOC_Os07g45260.1	glycosyl transferase 8 domain containing protein, putative, expressed	-3.124674768	0.0159386	Down
LOC_Os03g24510.1	glycosyl transferase 8 domain containing protein, putative, expressed	-2.606240887	0.04047631	Down
LOC_Os04g40150.1	glycosyl transferase family 17 protein, putative, expressed	-2.807915706	0.02828452	Down
LOC_Os02g32730.1	neutral/alkaline invertase, putative, expressed	-2.60641646	0.04065474	Down
LOC_Os04g43410.1	Os4bglu18 - monolignol beta-glucoside homologue, expressed	-6.325230488	0.00115341	Down
LOC_Os09g29600.1	OsWAK85 - OsWAK receptor-like cytoplasmic kinase OsWAK-RLCK, expressed	4.295161185	0.03526409	Up
LOC_Os03g53790.1	periplasmic beta-glucosidase precursor, putative, expressed	3.840125397	0.00400392	Up

Supplementary table S4 continued...

Gene ID	Putative functions	Log_2 fold change	P-Value	Regulation
LOC_Os02g03870.1	periplasmic beta-glucosidase precursor, putative, expressed	-5.02211413	0.00147213	Down
LOC_Os03g59330.1	polygalacturonase, putative, expressed	7.337753062	0.00366381	Up
LOC_Os06g35320.1	polygalacturonase, putative, expressed	-3.627510926	0.00647368	Down
LOC_Os06g35370.1	polygalacturonase, putative, expressed	-2.755123446	0.03139421	Down
LOC_Os06g40890.1	polygalacturonase, putative, expressed	-2.739956505	0.03182862	Down
LOC_Os01g33300.1	polygalacturonase, putative, expressed	-2.655246829	0.03708023	Down
LOC_Os01g07530.1	uncharacterized glycosyltransferase, putative, expressed	3.500502148	0.0367118	Up
LOC_Os03g59430.2	uncharacterized glycosyltransferase, putative, expressed	-3.337076731	0.01658141	Down
LOC_Os12g36870.1	uncharacterized glycosyltransferase, putative, expressed	-3.101716825	0.01958538	Down
LOC_Os12g02450.1	WRKY64, expressed	-6.032321287	0.03883352	Down
LOC_Os06g10970.1	xyloglucan fucosyltransferase, putative, expressed	-2.927415353	0.02336718	Down
**Carbohydrate_Tetrad**				
LOC_Os10g35070.1	alpha-galactosidase precursor, putative, expressed	-2.470219626	0.01181937	Down
LOC_Os03g04770.1	beta-amylase, putative, expressed	3.379330273	3.7142E-11	Up
LOC_Os10g32810.1	beta-amylase, putative, expressed	6.15189322	1.3636E-26	Up
LOC_Os01g34920.1	beta-galactosidase precursor, putative, expressed	#NAME?	7.25E-05	Down
LOC_Os10g19960.1	beta-galactosidase, putative, expressed	-1.323102996	0.0054408	Down
LOC_Os10g32980.1	CESA7 - cellulose synthase, expressed	-1.264451443	0.00430002	Down
LOC_Os09g25490.1	CESA9 - cellulose synthase, expressed	-1.03575222	0.01653698	Down
LOC_Os02g44230.1	CPuORF22 - conserved peptide uORF-containing transcript, expressed	-1.660215296	1.08E-08	Down
LOC_Os09g30120.1	CSLE1 - cellulose synthase-like family E, expressed	3.321570333	7.2672E-16	Up
LOC_Os02g44235.1	expressed protein	-1.707044152	0.03826314	Down
LOC_Os10g08022.1	fructose-bisphospate aldolase isozyme, putative, expressed	5.825836212	0.00083558	Up
LOC_Os06g39060.1	glucan endo-1,3-beta-glucosidase precursor, putative, expressed	Inf	0.00043168	Up
LOC_Os07g35480.2	glucan endo-1,3-beta-glucosidase precursor, putative, expressed	-2.767379815	0.00233064	Down
LOC_Os07g35510.1	glucan endo-1,3-beta-glucosidase precursor, putative, expressed	-4.152669971	1.60E-05	Down
LOC_Os08g14700.1	glucan endo-1,3-beta-glucosidase precursor, putative, expressed	-4.085555775	0.00784121	Down
LOC_Os02g09450.1	glycerophosphoryl diester phosphodiesterase family protein, putative, expres	5.720993847	3.5517E-06	Up
LOC_Os10g21110.1	glycosyl hydrolase family 10 protein, putative, expressed	Inf	0.00030363	Up
LOC_Os03g10478.1	glycosyl hydrolase family 10 protein, putative, expressed	-3.521351835	6.77E-05	Down

Supplementary table S4 continued...

Gene ID	Putative functions	Log_2 fold change	P-Value	Regulation
LOC_Os06g48180.1	glycosyl hydrolases family 16, putative, expressed	-2.856468702	6.89E-11	Down
LOC_Os04g33640.1	glycosyl hydrolases family 17, putative, expressed	-1.883596946	0.0003694	Down
LOC_Os02g50600.1	glycosyl transferase 8 domain containing protein, putative, expressed	-1.209827624	5.31E-06	Down
LOC_Os05g09500.1	hexokinase, putative, expressed	-3.339515934	4.7508E-16	Down
LOC_Os04g40990.1	malate synthase, glyoxysomal, putative, expressed	-1.720439014	0.0192721	Down
LOC_Os12g23170.1	Os12bglu38 - beta-glucosidase/beta-mannosidase/exoglucanase homologue,	Inf	0.00015031	Up
LOC_Os05g30350.1	Os5bglu22 - beta-glucosidase homologue, similar to G. max isohydroxyurate h	-2.019060364	0.04792078	Down
LOC_Os07g41650.1	pectinesterase, putative, expressed	-2.103257777	0.00018695	Down
LOC_Os01g33300.1	polygalacturonase, putative, expressed	Inf	0.00148135	Up
LOC_Os02g10300.1	polygalacturonase, putative, expressed	2.173269731	0.00631385	Up
LOC_Os06g40890.1	polygalacturonase, putative, expressed	Inf	2.6827E-06	Up
LOC_Os05g33840.1	transketolase, putative, expressed	-1.096269215	0.00068759	Down
LOC_Os06g11840.1	trehalose phosphatase, putative, expressed	-4.728699347	4.7956E-06	Down
LOC_Os02g54820.1	trehalose-6-phosphate synthase, putative, expressed	-1.002526986	2.37E-06	Down
LOC_Os09g20390.1	uncharacterized glycosyl hydrolase Rv2006/MT2062, putative, expressed	-2.34222123	3.58E-14	Down

Supplementary table S5: Lipid metaboism and Lipid Transfer Proteins (LTPs) genes differentially expressed in *bHLH142* ^{OE} compared to wild type.

**Lipid metabolism_MP**				
**Gene ID**	**Putative functions**	**Log_2 fold change**	**P-Value**	**Regulation**
LOC_Os04g32080.1	11-beta-hydroxysteroid dehydrogenase, putative, expressed	4.363748271	0.016113	Up
LOC_Os07g06800.1	3-oxo-5-alpha-steroid 4-dehydrogenase, putative, expressed	-2.916249144	0.029243	Down
LOC_Os03g30950.1	acyl-desaturase, chloroplast precursor, putative, expressed	3.103995322	0.024625	Up
LOC_Os07g28160.1	cytochrome P450 51, putative, expressed	9.317944581	8.28E-06	Up
LOC_Os08g03682.1	cytochrome P450, putative, expressed	3.221209157	0.039267	Up
LOC_Os07g41060.1	dihydroflavonol-4-reductase, putative, expressed	Inf	0.049457	Up
LOC_Os10g42220.1	enoyl-CoA hydratase/isomerase family protein, putative, expressed	5.107762877	0.001223	Up
LOC_Os10g42210.1	enoyl-CoA-hydratase, putative, expressed	4.646381854	0.001671	Up
LOC_Os06g40500.1	expressed protein	-2.559294607	0.046087	Down
LOC_Os04g28570.1	fatty acyl coA reductase, putative, expressed	4.491127577	0.010718	Up
LOC_Os04g55660.1	GDSL-like lipase/acylhydrolase, putative, expressed	5.224375298	0.000232	Up
LOC_Os07g39740.1	GDSL-like lipase/acylhydrolase, putative, expressed	-3.323530199	0.039541	Down
LOC_Os05g11810.1	gibberellin 2-beta-dioxygenase 1, putative, expressed	-2.645552827	0.03956	Down
LOC_Os09g17000.1	glycerophosphoryl diester phosphodiesterase family protein, putative, expressed	8.789054105	4.51E-05	Up
LOC_Os08g42390.1	glycerophosphoryl diester phosphodiesterase family protein, putative, expressed	-2.711949175	0.035461	Down
LOC_Os02g09450.1	glycerophosphoryl diester phosphodiesterase family protein, putative, expressed	-2.589952039	0.04138	Down
LOC_Os09g36520.1	ki1 protein, putative, expressed	-2.765834777	0.033059	Down
LOC_Os01g71800.1	lecithin cholesterol acyltransferase, putative, expressed	-3.472403916	0.014149	Down
LOC_Os04g41200.1	lipase, putative, expressed	-2.567495435	0.043389	Down
LOC_Os02g55910.1	monogalactosyldiacylglycerol synthase, putative, expressed	-2.73313331	0.032379	Down
LOC_Os01g12160.1	OsGH3.3 - Probable indole-3-acetic acid-amido synthetase, expressed	-2.883417258	0.030445	Down
LOC_Os08g40590.2	oxysterol-binding protein, putative, expressed	-2.599508378	0.041037	Down
LOC_Os05g21180.2	phosphatidic acid phosphatase-related, putative, expressed	-2.692176708	0.034728	Down
LOC_Os03g24160.1	phosphatidylinositol-4-phosphate 5-kinase, putative, expressed	-2.737543066	0.032442	Down
LOC_Os03g24170.1	phosphatidylinositol-4-phosphate 5-kinase, putative, expressed	-2.583571951	0.0426	Down
LOC_Os03g62410.1	phospholipase D, putative, expressed	-2.52462148	0.046974	Down
LOC_Os01g46270.1	wax synthase isoform 3, putative, expressed	Inf	0.012223	Up

Supplementary table S5 continued...

Gene ID	Putative functions	Log_2 fold change	P-Value	Regulation
**Lipid transfer protein_MP**				
LOC_Os11g24070.1	LTPL10 - Protease inhibitor/seed storage/LTP family protein precursor, expressed	3.197498927	0.040247	Up
LOC_Os03g14630.1	LTPL106 - Protease inhibitor/seed storage/LTP family protein precursor, expressed	4.571500241	0.005134	Up
LOC_Os12g02320.1	LTPL12 - Protease inhibitor/seed storage/LTP family protein precursor, expressed	2.798208868	0.03341	Up
LOC_Os07g46210.1	LTPL2 - Protease inhibitor/seed storage/LTP family protein precursor, expressed	5.34298971	0.013605	Up
LOC_Os10g05720.1	LTPL37 - Protease inhibitor/seed storage/LTP family protein precursor, expressed	3.676535712	0.017144	Up
LOC_Os08g43290.1	LTPL44 - Protease inhibitor/seed storage/LTP family protein precursor, expressed	4.569449501	0.011692	Up
LOC_Os09g35700.1	LTPL45 - Protease inhibitor/seed storage/LTP family protein precursor, expressed	3.876878027	0.010751	Up
LOC_Os11g37280.1	LTPL68 - Protease inhibitor/seed storage/LTP family protein precursor, expressed	2.515329791	0.048468	Up
LOC_Os10g11750.1	LTPL89 - Protease inhibitor/seed storage/LTP family protein precursor, expressed	-3.080263493	0.017895	Down
LOC_Os08g43240.1	LTPL97 - Protease inhibitor/seed storage/LTP family protein precursor, expressed	4.152306149	0.006665	Up
**Lipid metabolism_Tetrad**				
LOC_Os04g32080.1	11-beta-hydroxysteroid dehydrogenase, putative, expressed	Inf	0.007249	Up
LOC_Os12g13930.1	3-oxoacyl-reductase, chloroplast precursor, putative, expressed	-7.082212332	1.97E-53	Down
LOC_Os12g42280.1	9-cis-epoxycarotenoid dioxygenase 1, chloroplast precursor, putative, expressed	-2.910999325	0.000241	Down
LOC_Os05g07090.1	acyl-coenzyme A dehydrogenase, mitochondrial precursor, putative, expressed	-2.476942007	5.21E-05	Down
LOC_Os12g26290.2	alpha-DOX2, putative, expressed	-3.55233685	9.94E-15	Down
LOC_Os10g35070.1	alpha-galactosidase precursor, putative, expressed	-2.470219626	0.011819	Down
LOC_Os04g43200.1	caleosin related protein, putative, expressed	-3.323715513	0.008045	Down
LOC_Os02g47470.1	cytochrome P450, putative, expressed	-5.773611769	0.026085	Down
LOC_Os10g42220.1	enoyl-CoA hydratase/isomerase family protein, putative, expressed	9.417524576	4.23E-23	Up
LOC_Os10g42210.1	enoyl-CoA-hydratase, putative, expressed	9.800331216	1.07E-29	Up
LOC_Os01g11650.1	GDSL-like lipase/acylhydrolase, putative, expressed	-2.735237957	1.63E-05	Down
LOC_Os05g11910.1	GDSL-like lipase/acylhydrolase, putative, expressed	-1.049360047	1.19E-07	Down
LOC_Os05g39220.1	GDSL-like lipase/acylhydrolase, putative, expressed	-6.32502171	0.003623	Down
LOC_Os06g50950.1	GDSL-like lipase/acylhydrolase, putative, expressed	-1.150017958	0.001713	Down
LOC_Os08g45150.1	GDSL-like lipase/acylhydrolase, putative, expressed	-1.569270464	2.71E-11	Down
LOC_Os07g07420.1	gibberellin 20 oxidase 1-B, putative, expressed	1.221127363	0.034302	Up

Supplementary table S5 continued...

Gene ID	Putative functions	Log_2 fold change	P-Value	Regulation
LOC_Os02g09450.1	glycerophosphoryl diester phosphodiesterase family protein, putative, expressed	5.720993847	3.55E-06	Up
LOC_Os05g49830.1	lipase class 3 family protein, putative, expressed	1.569796053	0.000501	Up
LOC_Os02g54010.1	lipase class 3 family protein, putative, expressed	-1.155642763	4.90E-07	Down
LOC_Os04g59540.1	phosphatidylinositol-4-phosphate 5-Kinase, putative, expressed	-1.547387486	0.032045	Down
LOC_Os09g38320.1	phytoene synthase, chloroplast precursor, putative, expressed	-1.088823489	0.006265	Down
LOC_Os05g33840.1	transketolase, putative, expressed	-1.096269215	0.000688	Down
LOC_Os01g46270.1	wax synthase isoform 3, putative, expressed	#NAME?	0.000246	Down
**Lipid transfer protein_Tetrad**				
LOC_Os03g14654.1	LTPL108 - Protease inhibitor/seed storage/LTP family protein precursor, expressed	inf	0.030634	Down
LOC_Os03g50960.1	LTPL118 - Protease inhibitor/seed storage/LTP family protein precursor, expressed	-2.603686768	0.042639	Down
LOC_Os04g52260.1	LTPL124 - Protease inhibitor/seed storage/LTP family protein precursor, expressed	-1.612896244	0.046781	Down
LOC_Os05g40010.1	LTPL17 - Protease inhibitor/seed storage/LTP family protein precursor, expressed	-1.690071074	0.013478	Down
LOC_Os07g46210.1	LTPL2 - Protease inhibitor/seed storage/LTP family protein precursor, expressed	-2.0663783	5.45E-25	Down
LOC_Os10g05720.1	LTPL37 - Protease inhibitor/seed storage/LTP family protein precursor, expressed	-3.219411522	0.000584	Down
LOC_Os04g09520.1	LTPL59 - Protease inhibitor/seed storage/LTP family protein precursor, putative, expressed	inf	5.24E-05	Down
LOC_Os07g39640.1	LTPL64 - Protease inhibitor/seed storage/LTP family protein precursor, expressed	-5.549836794	1.42E-17	Down
LOC_Os11g37280.1	LTPL68 - Protease inhibitor/seed storage/LTP family protein precursor, expressed	-10.0801786	1.6E-166	Down
LOC_Os04g38840.1	LTPL81 - Protease inhibitor/seed storage/LTP family protein precursor, expressed	1.005218279	0.001275	Up
LOC_Os03g45150.1	LTPL93 - Protease inhibitor/seed storage/LTP family protein precursor, expressed	1.822214244	2.01E-06	Up

Supplementary table S6: Different classes of cell wall modification related genes differentially expressed in *bHLH142*^{OE} compared to wild type .

**Cell wall modification_Tetrad**				
**Gene ID**	**Putative functions**	**log_2 fold change**	**p-value**	**Regulation**
LOC_Os03g01640.1	expansin precursor, putative, expressed	6.713725846	0.000712278	Up
LOC_Os08g44790.1	expansin precursor, putative, expressed	3.958838344	0.015925517	Up
LOC_Os10g40090.1	expansin precursor, putative, expressed	7.447773957	2.31763E-30	Up
LOC_Os01g14940.1	invertase/pectin methylesterase inhibitor family protein, putative, expressed	Inf	0.00047139	Up
LOC_Os01g20970.1	invertase/pectin methylesterase inhibitor family protein, putative, expressed	Inf	0.000395322	Up
LOC_Os01g50810.1	invertase/pectin methylesterase inhibitor family protein, putative, expressed	7.322773965	1.02726E-05	Up
LOC_Os02g01310.1	invertase/pectin methylesterase inhibitor family protein, putative, expressed	3.069262334	0.034196203	Up
LOC_Os05g20570.1	invertase/pectin methylesterase inhibitor family protein, putative, expressed	Inf	9.25412E-18	Up
LOC_Os05g46530.1	invertase/pectin methylesterase inhibitor family protein, putative, expressed	8.985236034	2.84287E-46	Up
LOC_Os06g05209.1	pectate lyase precursor, putative, expressed	6.592163866	0.001357631	Up
LOC_Os06g05260.1	pectate lyase precursor, putative, expressed	6.369771445	1.91889E-05	Up
LOC_Os06g38510.2	pectate lyase precursor, putative, expressed	3.325955213	0.000379083	Up
LOC_Os08g04650.1	pectinesterase inhibitor domain containing protein, expressed	Inf	0.03876563	Up
LOC_Os03g19610.1	pectinesterase, putative, expressed	3.352858062	0.016748464	Up
LOC_Os04g38560.1	pectinesterase, putative, expressed	Inf	8.68687E-13	Up
LOC_Os04g54850.1	pectinesterase, putative, expressed	Inf	0.035494736	Up
LOC_Os07g49100.1	pectinesterase, putative, expressed	7.777856183	1.02653E-07	Up
LOC_Os08g34910.1	pectinesterase, putative, expressed	3.096341868	0.000482145	Up
LOC_Os09g26360.1	pectinesterase, putative, expressed	5.234116345	0.012200308	Up
LOC_Os11g45720.1	pectinesterase, putative, expressed	6.85695873	8.24471E-11	Up
LOC_Os11g45730.1	pectinesterase, putative, expressed	6.502221741	5.94286E-06	Up
LOC_Os12g37660.1	pectinesterase, putative, expressed	5.654224834	0.002066158	Up
LOC_Os07g41650.1	pectinesterase, putative, expressed	-2.103257777	0.000186946	Down
**Cell wall modification_MP**				
**Gene ID**	**Putative functions**	**log_2 fold change**	**P-value**	**Regulation**
LOC_Os04g46650.1	expansin precursor, putative, expressed	Inf	0.021061483	Up

Supplementary table S6 continued...

Gene ID	Putative functions	log_2 fold change	p-value	Regulation
LOC_Os03g06000.1	expansin precursor, putative, expressed	4.119435231	0.006073319	Up
LOC_Os10g40710.1	expansin precursor, putative, expressed	-5.799958577	0.000366716	Down
LOC_Os03g01610.1	expansin precursor, putative, expressed	-4.415581095	0.001276605	Down
LOC_Os03g01630.1	expansin precursor, putative, expressed	-4.268726	0.001728098	Down
LOC_Os08g44790.1	expansin precursor, putative, expressed	-2.904572503	0.023738946	Down
LOC_Os03g01640.1	expansin precursor, putative, expressed	-2.839292793	0.026681231	Down
LOC_Os06g05260.1	pectate lyase precursor, putative, expressed	-3.290744825	0.011528707	Down
LOC_Os06g05272.1	pectate lyase precursor, putative, expressed	-3.147475661	0.015579222	Down
LOC_Os06g05209.1	pectate lyase precursor, putative, expressed	-2.98372548	0.020539069	Down
LOC_Os02g12300.1	pectate lyase precursor, putative, expressed	-2.749170016	0.031417123	Down
LOC_Os11g03740.1	pectinesterase inhibitor domain containing protein, expressed	-3.085060351	0.019999649	Down
LOC_Os08g04650.1	pectinesterase inhibitor domain containing protein, expressed	-2.701103614	0.03421241	Down
LOC_Os03g01020.1	pectinesterase inhibitor domain containing protein, putative, expressed	-2.686207276	0.035263952	Down
LOC_Os12g37660.1	pectinesterase, putative, expressed	-2.750176126	0.031291752	Down
LOC_Os11g45720.1	pectinesterase, putative, expressed	-2.702421284	0.033975465	Down
LOC_Os03g28090.1	pectinesterase, putative, expressed	-2.611732924	0.040146866	Down
LOC_Os08g34910.1	pectinesterase, putative, expressed	-2.567816984	0.043000594	Down
LOC_Os07g49100.1	pectinesterase, putative, expressed	-2.531090729	0.045810575	Down
LOC_Os04g58080.1	polygalacturonase inhibitor 3 precursor, putative, expressed	4.810814624	0.014896686	Up
LOC_Os03g59330.1	polygalacturonase, putative, expressed	7.337753062	0.003663812	Up
LOC_Os06g35320.1	polygalacturonase, putative, expressed	-3.627510926	0.006473676	Down
LOC_Os06g35370.1	polygalacturonase, putative, expressed	-2.755123446	0.031394207	Down
LOC_Os06g40890.1	polygalacturonase, putative, expressed	-2.739956505	0.031828616	Down
LOC_Os05g29740.1	invertase/pectin methylesterase inhibitor family protein, putative, expressed	-3.403937767	0.009369706	Down
LOC_Os02g01300.1	invertase/pectin methylesterase inhibitor family protein, putative, expressed	-3.325413136	0.011653318	Down
LOC_Os01g20970.1	invertase/pectin methylesterase inhibitor family protein, putative, expressed	-2.95777164	0.02153571	Down
LOC_Os10g10700.1	invertase/pectin methylesterase inhibitor family protein, putative, expressed	-2.949245109	0.022243192	Down
LOC_Os03g61530.1	invertase/pectin methylesterase inhibitor family protein, putative, expressed	-2.916086562	0.023879682	Down
LOC_Os12g37480.1	invertase/pectin methylesterase inhibitor family protein, putative, expressed	-2.671561306	0.037333888	Down

Supplementary table S7: List of ROS and cell death related genes differentially expressed in *bHLH142* ^{OE} compared to wild type.				
**ROS_Tetrad**				
**Gene ID**	**Putative functions**	**Log_2 fold change**	**P-Value**	**Regulation**
LOC_Os02g50770.1	peroxidase precursor, putative, expressed	Inf	0.005563	Up
LOC_Os03g05770.1	peroxidase precursor, putative, expressed	6.904548188	0.000229	Up
LOC_Os08g20730.1	peroxidase precursor, putative, expressed	1.471669657	4.59E-18	Down
LOC_Os01g57730.1	peroxidase precursor, putative, expressed	-1.224873609	0.005311	Down
LOC_Os03g55410.1	peroxidase precursor, putative, expressed	-4.546605673	2.84E-05	Down
LOC_Os04g59190.1	peroxidase precursor, putative, expressed	-1.348744159	0.000751	Down
LOC_Os04g59200.1	peroxidase precursor, putative, expressed	-3.430203947	0.045083	Down
LOC_Os05g04500.1	peroxidase precursor, putative, expressed	-2.09107278	0.001321	Down
LOC_Os06g35520.1	peroxidase precursor, putative, expressed	-5.632164468	1.97E-15	Down
LOC_Os06g48020.1	peroxidase precursor, putative, expressed	-2.14736106	0.040663	Down
LOC_Os12g02080.1	peroxidase precursor, putative, expressed	-2.179843371	0.008742	Down
LOC_Os10g38470.1	glutathione S-transferase, putative, expressed	-5.058588728	0.002867	Down
LOC_Os03g17870.1	metallothionein, putative, expressed	-4.253500413	0.000303	Down
LOC_Os11g47809.1	metallothionein, putative, expressed	-6.312235555	4.60E-18	Down
**Cell Death_Tetrad**				
LOC_Os12g26290.2	alpha-DOX2, putative, expressed	-3.55233685	9.94E-15	Down
LOC_Os09g11480.2	AP2 domain containing protein, expressed	-5.12508414	0.014653	Down
LOC_Os04g41620.1	CHIT2 - Chitinase family protein precursor, expressed	inf	0.046106	Down
LOC_Os04g41680.1	CHIT3 - Chitinase family protein precursor, expressed	-5.892910698	0.018175	Down
LOC_Os08g16910.1	dehydrogenase, putative, expressed	inf	0.000604	Down
LOC_Os04g14710.1	flavin-containing monooxygenase family protein, putative, expressed	1.164336054	2.34E-08	Up
LOC_Os05g09500.1	hexokinase, putative, expressed	-3.339515934	4.75E-16	Down
LOC_Os03g03700.1	MLO domain containing protein, putative, expressed	-1.422059872	0.014056	Down
LOC_Os06g46560.1	myb-like DNA-binding domain containing protein, expressed	5.891724148	0.021844	Up
LOC_Os02g34970.1	no apical meristem protein, putative, expressed	-1.588347717	1.30E-05	Down

Supplementary table S7

Gene ID	Putative functions	Log_2 fold change	P-Value	Regulation
**ROS_MP**				
**Gene ID**	**Putative functions**	**Log_2 fold change**	**P-value**	**Regulation**
LOC_Os06g46799.1	peroxidase precursor, putative, expressed	6.327222395	0.000403	Up
LOC_Os06g48030.1	peroxidase precursor, putative, expressed	2.885730125	0.041391	Up
LOC_Os06g27850.1	peroxidase precursor, putative, expressed	-2.716351038	0.033243	Down
LOC_Os05g40190.1	thioredoxin, putative, expressed	-3.035232882	0.044984	Down
LOC_Os01g48540.1	glyoxal oxidase-related, putative, expressed	-3.181895262	0.014565	Down
LOC_Os04g33660.2	bifunctional monodehydroascorbate reductase and carbonic anhydrasenectarin-3 precursor, putative, expressed	3.32091745	0.014938	Up
LOC_Os09g28150.1	bifunctional monodehydroascorbate reductase and carbonic anhydrasenectarin-3 precursor, putative, expressed	-2.936565685	0.023196	Down
LOC_Os11g05520.1	bifunctional monodehydroascorbate reductase and carbonic anhydrasenectarin-3 precursor, putative, expressed	-2.549947738	0.045568	Down
**Cell Death_MP**				
LOC_Os06g46560.1	myb-like DNA-binding domain containing protein, expressed	-2.588557416	0.041626	Down
LOC_Os04g43440.1	NB-ARC/LRR disease resistance protein, putative, expressed	2.987093249	0.041311	Up
LOC_Os06g43000.1	nitrate-induced NOI protein, expressed	-2.591861788	0.042807	Down
LOC_Os03g62410.1	phospholipase D, putative, expressed	-2.52462148	0.046974	Down
LOC_Os07g35140.1	receptor-like serine-threonine protein kinase, putative, expressed	3.994202155	0.03929	Up
LOC_Os11g29210.1	resistance-gene-interacting protein, putative, expressed	-3.295849851	0.021196	Down

Supplementary table S8: Lignin biosynthesis and water transport related genes differentially expressed in *bHLH142* ^{OE} compared to wild type.

Lignin biosynthesis_tetrad				
**Gene ID**	**Putative functions**	**Log_2 fold change**	**P-value**	**Regulation**
LOC_Os01g18110.1	cinnamoyl CoA reductase, putative, expressed	-4.17866518	0.011572	Down
LOC_Os09g08720.1	cinnamoyl CoA reductase, putative, expressed	-1.179630516	0.000842	Down
LOC_Os01g62490.1	laccase precursor protein, putative, expressed	-2.212023777	0.004564	Down
LOC_Os03g16610.1	laccase precursor protein, putative, expressed	-1.863163354	0.002925	Down
LOC_Os05g35290.1	phenylalanine ammonia-lyase, putative, expressed	-1.867190419	0.033822	Down
**Water transport_MP**				
**Gene ID**	**Putative functions**	**Log_2 fold change**	**P-value**	**Regulation**
LOC_Os02g51110.1	aquaporin protein, putative, expressed	2.80420128	0.043542	Up
LOC_Os04g44060.1	aquaporin protein, putative, expressed	-4.799804648	0.002237	Down
LOC_Os02g44080.1	aquaporin protein, putative, expressed	-3.082498487	0.020567	Down
LOC_Os03g20410.1	aquaporin SIP2-1, putative, expressed	-3.148948498	0.015376	Down
LOC_Os10g26470.1	sucrose transporter, putativ, expressed	-2.602598626	0.040685	Down
